# The Influence of Regional Geophysical Resource Variability on the Value of Single- and Multistorage Technology Portfolios

**DOI:** 10.1021/acs.est.3c10188

**Published:** 2024-07-15

**Authors:** Anna X. Li, Edgar Virgüez, Jacqueline A. Dowling, Alicia Wongel, Dominic Covelli, Tyler H. Ruggles, Natasha Reich, Nathan S. Lewis, Ken Caldeira

**Affiliations:** † Division of Chemistry and Chemical Engineering, California Institute of Technology, Pasadena, California 91125, United States; ‡ Department of Global Ecology, Carnegie Institution for Science, Stanford, California 94305, United States; § Beckman Institute, California Institute of Technology, Pasadena, California 91125, United States; ∥ Gates Ventures LLC, Kirkland, Washington 98033, United States

**Keywords:** Least-cost electricity systems, energy storage technologies, wind generation, solar generation, decarbonized electricity systems

## Abstract

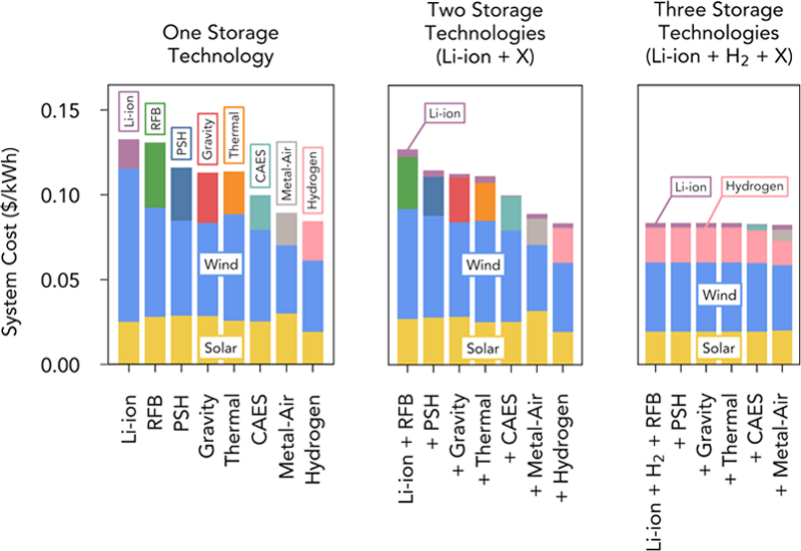

A stylized macro-scale energy model of least-cost electricity systems relying only on wind and solar generation was used to assess the value of different storage technologies, individually and combined, for the contiguous U.S. as well as for four geographically diverse U.S. load-balancing regions. For the contiguous U.S. system, at current costs, when only one storage technology was deployed, hydrogen energy storage produced the lowest system costs, due to its energy-capacity costs being the lowest of all storage technologies modeled. Additional hypothetical storage technologies were more cost-competitive than hydrogen (long-duration storage) only at very low energy-capacity costs, but they were more cost-competitive than Li-ion batteries (short-duration storage) at relatively high energy- and power-capacity costs. In all load-balancing regions investigated, the least-cost systems that included long-duration storage had sufficient energy and power capacity to also meet short-duration energy and power storage needs, so that the addition of short-duration storage as a second storage technology did not markedly reduce total system costs. Thus, in electricity systems that rely on wind and solar generation, contingent on social and geographic constraints, long-duration storage may cost-effectively provide the services that would otherwise be provided by shorter-duration storage technologies.

## Abstract 2

This study provides insight into the value of different storage technology portfolios in least-cost electricity systems that rely on only wind and solar generation.

## Introduction

Energy storage is an important component of reliable, cost-effective, deeply decarbonized electricity systems that rely on substantial generation from variable renewable energy resources, such as wind and solar energy.^1^ Energy storage technologies differ in their siting and supply chain constraints, sociopolitical challenges, round-trip efficiency, energy-capacity cost, power-capacity cost, and storage duration.^2,3^ Consequently, many modeled least-cost, deeply decarbonized electricity systems contain multiple storage technologies.^3,4^

Short-duration energy storage technologies have relatively low power-capacity costs and thus are cost-effective for frequent (hourly) charging and discharging to smooth sharp peaks in electricity generation or demand.^5,6^ Currently, lithium-ion (Li-ion) batteries with 1 to 4 h durations are the most widely deployed short-duration storage technology.^7,8^

In contrast, long-duration (>100 h) storage technologies such as pumped-storage hydropower (PSH), compressed air energy storage, and electrolytic hydrogen have relatively high power-capacity costs and relatively low energy-capacity costs, as compared to other commercialized storage technologies on the market.^9^ Herein, energy-capacity costs refer to overnight capital costs for energy storage in $/kW h, and power-capacity costs refer to overnight capital costs for power capacity in $/kW, for a given storage technology. Due to these low energy-capacity costs, long-duration energy storage can compensate for sustained weather-related events that last days or weeks and can cost-effectively buffer seasonal or interannual variability in renewable resource availability, even if depleted relatively infrequently in any year.^10−13^

**Figure 1:**
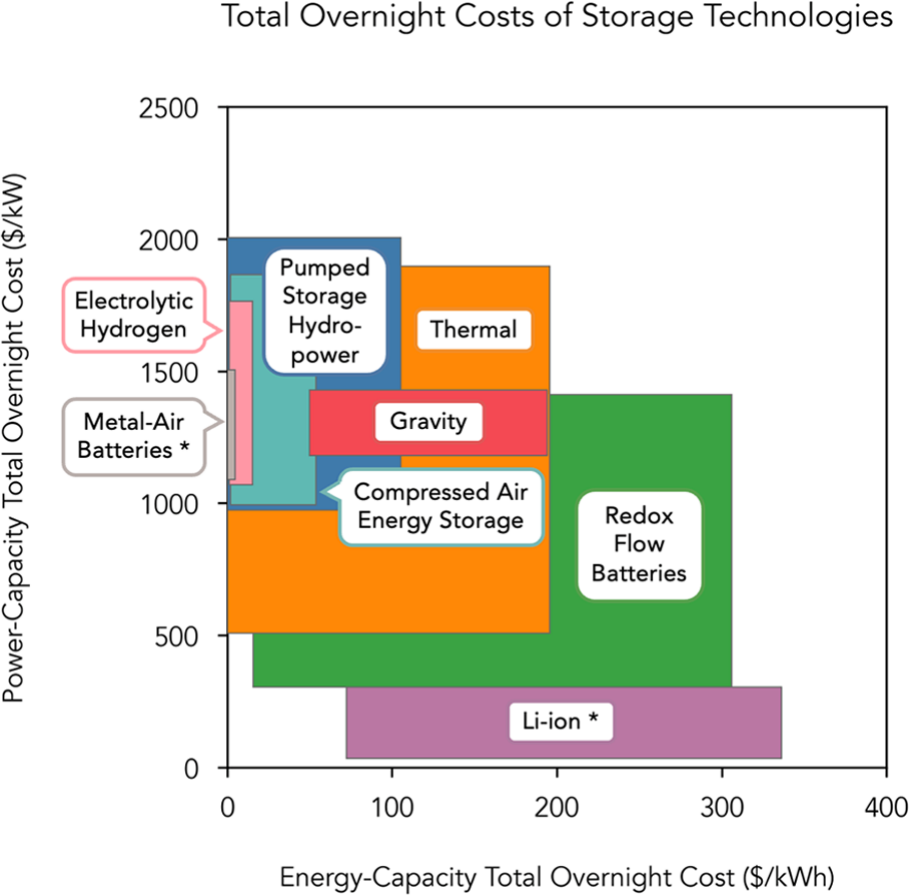
Energy-capacity costs and power-capacity costs of energy storage technologies. Ranges of total installed energy- and power-capacity costs of different storage technologies. Numerical values and sources are provided in Table S1. *Energy-capacity and power-capacity costs were combined to obtain the total cost of Li-ion battery and metal–air battery storage.

Another group of demonstrated storage technologies can potentially provide mid-duration storage, i.e., storage for durations of days to weeks. This group is characterized by intermediate energy- and power-capacity costs (Figure 1, Table S1). For example, deployed redox-flow batteries have durations up to 10 h and can theoretically be designed to provide storage for even longer durations.^14^ Thermal energy storage can reportedly provide storage durations from 8 to 192 h (8 days), and commercial iron–air batteries are projected to provide durations from 100 to 150 h at a combined energy- and power-capacity cost of <$20/kW h.^15−17^ Gravity-based energy storage has the potential to store energy for >12 h.^18^

When long-duration storage is used in addition to short-duration storage, total system costs are reduced for wind- and solar-based electricity systems that meet hourly averaged demand in full for over a year of resource variability.^10−13^ However, the value and role of deploying two or more storage technologies are controversial. For a United Kingdom (U.K.) electricity system modeled with mainly wind and solar generation along with existing nuclear resources, in conjunction with demand flexibility, almost all optimal storage portfolios in least-cost reliable systems used only Li-ion batteries and electrolytic hydrogen, with compressed air energy storage deployed only in scenarios with electricity oversupply within a specific range.^19^ However, when projected 2050 costs were assumed for seven independent United States (U.S.) electricity system load-balancing regions with 100% renewable or carbon-free resources (wind, solar, nuclear, hydro, biomass, and geothermal), the optimal storage portfolio contained 4 types of storage technologies with mutually different durations.^20^ When 2050 costs were assumed for storage technologies in three different U.S. load-balancing regions that rely primarily on wind and solar generation, with constrained amounts of natural gas generation, only low-cost Li-ion and redox-flow batteries were used for storage, obviating a need for longer-duration storage technologies including electrolytic hydrogen, thermal energy storage, or metal–air batteries.^20^ In scenarios in which Li-ion batteries, redox-flow batteries, and a single long-duration storage technology (thermal, metal–air, or hydrogen) were available, the optimal storage portfolio partially substituted deployment of Li-ion batteries with redox-flow batteries and the long-duration storage technology.^21^ Here, we aim to identify generalizable findings for least-cost energy storage portfolios, based on the fundamental geophysical variability of the resources available in different load-balancing regions over the time scales required to meet hourly averaged demand in full over a year.

The value of a storage technology was measured by the technology's impact on total costs of a least-cost electricity system based solely on wind and/or solar generation that met hourly averaged demand in full for an entire year. To assess the value of different storage technology portfolios in a simple, transparent fashion, we used a stylized macro-scale energy model^10,22−24^ to obtain asset capacities and dispatch schedules in a least-cost stylized electricity system that relies only on wind and solar generation, assuming no previously existing grid technologies. The stylized electricity system relied solely on wind and/or solar generation, with no transmission constraints, no reserve margins, and no firm dispatchable fossil generation such as natural gas, to transparently reveal the fundamental geophysical dynamical relationships between energy storage and wind and/or solar resource variability over a variety of geographically distributed regions in the U.S. Hourly averaged resource availability data and concurrent hourly demand data were obtained for one year from a weather reanalysis data set for the contiguous U.S. (CONUS) as well as for four independent system operator (ISO) regions within CONUS that were characterized by very different qualities and quantities of wind and solar resources (Table S2). The modeling was subject to the strict constraint that 100% of hourly averaged demand was met for every hour in the simulated year. Each region was represented by a single node, which reduces generation variability and thus decreases the value of storage technologies compared to more realistic representations of the grid.

The modeled energy storage technologies were divided qualitatively into three categories: short-, mid-, and long-duration storage. Li-ion batteries were used to represent a short-duration storage technology, whereas electrolytic hydrogen represented a long-duration storage technology. The electrolyzers used electricity to produce hydrogen, which was stored in underground salt caverns and subsequently utilized in fuel cells. Various technologies represented potential mid-duration storage systems: redox-flow batteries (RFB), compressed air energy storage (CAES), pumped-storage hydropower (PSH), thermal energy storage, gravity energy storage, and metal–air battery storage. In the modeled systems, the energy and power capacities of these mid-duration storage technologies were independently sizable, potentially allowing them to be optimized to also provide short- or long-term storage.

Figure S1 shows the electricity sources, storage, and sinks (electricity demand or curtailed power) in the model architecture. The modeled electricity systems contained portfolios of 1–3 storage technologies that comprised various combinations of the defined short-, mid-, and long-duration storage technologies. The robustness and generality of the findings were evaluated by parameterizing the energy- and power-capacity costs of a hypothetical storage technology (*Storage X*) across wide ranges for these geographically diverse U.S. load-balancing regions.

## Methods

### Wind and Solar Generation Data

The regions considered in this analysis were the contiguous U.S. (CONUS) and four subnational independent system operator (ISO) geographic regions (CAISO, ERCOT, ISO-NE, and MISO). Hourly capacity factors for solar and wind data for each region during 2018 were generated using reanalysis data with a grid-cell resolution of 0.5° latitude by 0.625° longitude from the Modern-Era Retrospective analysis for Research and Applications, Version 2 (MERRA-2).^25^ Solar capacities of utility-scale photovoltaics were calculated for a single-axis tracking system with 0–45° of tilt. Wind capacity factors for geographic regions with the top 25% generation potential of land-based wind turbines were calculated assuming a General Electric 1.6–100 turbine with a 1.6 MW nameplate capacity.^26−28^Table S2 presents calculated average wind and solar capacity factors for CONUS, CAISO, ERCOT, ISO-NE, and MISO.

### Electricity Demand Data

Electricity demand data for the CONUS and ISO regions were obtained from hourly data for 2018 from the U.S. Energy Information Administration (EIA).^29^ The EIA data was cleaned, and missing values were replaced using the multiple imputation by chained equations (MICE) method.^30^

### Cost and Technological Assumptions

A complete description of the model formulation is included in the Supporting Information. Base case costs for solar and wind generation were taken from the National Renewable Energy Laboratory Annual Technology Baseline (NREL ATB)report (Table S3).^31^Tables S3 and S4 present the base case costs, efficiencies, and other characteristics for storage technologies used in the model. Parameters for Li-ion batteries, hydrogen storage, RFB, CAES, PSH, and thermal energy storage were taken from a 2021 NREL analysis of long-duration energy storage technologies.^10^ Gravity energy storage parameters were taken from the Pacific Northwest National Laboratory’s 2020 Grid energy Storage Technology Cost and Performance Assessment, with energy- and power-capacity costs separated by linear regression, using cost estimates for 1000 MW storage systems at various durations.^14^ The total overnight cost for metal–air batteries was taken from press releases by Form Energy, and the O&M costs and round-trip efficiency were taken from the 2022 MIT Future of Energy Storage Report.^17,32^

Li-ion batteries and metal–air batteries were each modeled using one total cost because the energy and power components of these batteries are nonseparable. Li-ion batteries were modeled with a duration of 4 h, due to technological constraints.^8^ Metal–air batteries were assumed to be iron–air batteries with a duration of 100 h, matching the duration claimed by Form Energy projects.^17^

RFB, PSH, thermal energy storage, and gravity energy storage were modeled with separate energy- and power-capacity components. Charging and discharging these technologies depend on the same physical asset, so only one power-capacity cost was used for each system. RFB costs were based on a vanadium-based redox-flow battery. PSH was assumed to be a closed-loop pumped hydroelectric storage system using upper and lower water reservoirs. Thermal energy storage was modeled after a pumped-thermal energy storage system, utilizing molten-salt technology for heat storage. Gravity energy storage was assumed to be a system using cranes to lift heavy bricks.

CAES and hydrogen storage were modeled with separate energy- and power-capacity components, but charging processes were assigned different power-capacity costs than the ones assigned to discharging. An adiabatic CAES (A-CAES) system was assumed, with air compressed into a salt dome cavern, the heat of compression stored in thermal energy storage, and power generated by reheating air with stored thermal energy. For hydrogen storage, proton-exchange membrane (PEM) electrolyzers were assumed to split water; hydrogen was assumed to be stored underground in salt caverns; and hydrogen was combined with O_2_*(g)* in PEM fuel cells to generate power. Hydrogen storage was conservatively described using the leakage rate characteristic of hydrogen stored in pipelines, as opposed to the lower leakage rate that is likely characteristic of hydrogen stored in salt caverns.

Figure S2 shows the base case costs assumed for the short-, mid-, and long-duration storage technologies considered in this study (Table S4).^9,14,17,32^ Li-ion batteries use the same technological component for energy and power capacities, so their energy and power characteristics are not mutually separable. The capital costs of such batteries therefore depend on whether the batteries are sized to meet power demand or energy demand (Figure S3). Li-ion batteries were modeled with a fixed duration of 4 h, being sized to meet short-term power demands, because they are not competitive with other storage technologies on energy-capacity costs, especially if used relatively infrequently.^8^ In accord with currently proposed iron–air battery projects, metal–air batteries were constrained to a fixed duration of 100 h, and thus were sized primarily to meet energy demand over their storage duration.^17^ The durations of the other storage technologies were not specifically constrained in the modeling. Costs of a hypothetical *Storage X* technology were parameterized over the entire range of energy- and power-capacity costs shown in Figure S2, to address the uncertainty of storage costs on the market and assess the generalizability of the findings regarding the value of different storage technology portfolios in these stylized electricity systems.

## Results

### CONUS Storage Portfolios

**Figure 2:**
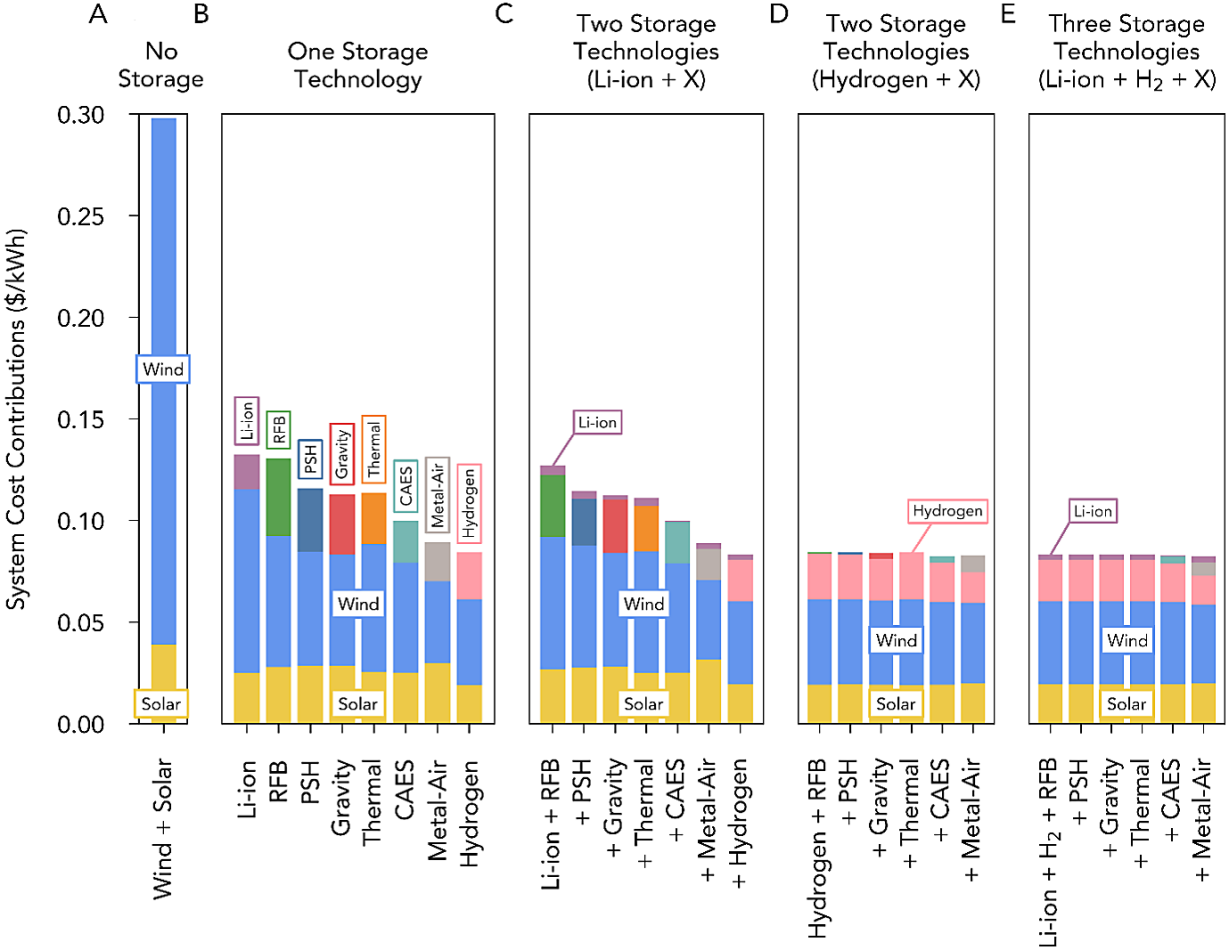
System costs for combinations of short-, mid-, and long-duration storage for the contiguous U.S. Cost contributions of technologies in wind and solar generation-based systems with one, two, and three storage technologies. Tables S7–S10 support this figure. System costs when: (A) no storage technologies were deployed and the least-cost 100% reliable system relied only on wind and solar generation. (B) Only one storage technology was available: Li-ion batteries, redox-flow batteries (RFB), pumped-storage hydropower (PSH), gravity energy storage, thermal energy storage, compressed air energy storage (CAES), metal–air battery storage, or hydrogen energy storage. (C) Two storage technologies were available: Li-ion batteries with the second storage technology consisting of either a mid-duration storage technology or hydrogen energy storage. (D) Two storage technologies were available: hydrogen energy storage with the second storage technology consisting of a mid-duration storage technology. (E) Three storage technologies were available: Li-ion batteries and hydrogen energy storage, with the third storage technology consisting of a mid-duration storage technology.

Figure 2A shows the cost contributions of generation assets in a least-cost system that relies solely on wind and solar generation, with no storage technologies included. In this system, total system costs are dominated by costs attributed to wind generation capacity. Figure 2B–E shows the cost contributions of generation and storage assets of least-cost systems optimized *de novo* in each case. Figure 2B shows scenarios in which one storage technology (short-, mid-, or long-duration) was deployed. Figure 2C,D shows scenarios in which mid-duration storage was deployed as well as either short- or long-duration storage, respectively. Figure 2E shows scenarios in which short-, mid-, and long-duration storage were deployed. Figure S3 shows analogous results for regional ISOs.

Using base-case cost assumptions, the least-cost system that used only short-duration storage (i.e., Li-ion and/or RFB) had the highest total system costs (Figure 2) of all scenarios with storage technologies evaluated, representing a ∼55% reduction in total CONUS system costs as compared to the least-cost 100% reliable system that had only wind and solar generation without storage. The high total system costs resulted primarily from the large wind generation capacity that was still required to meet demand in full, given the seasonal and weather-related variability of the wind resource over CONUS.^33^ System costs were reduced when any other type of storage was used, either instead of or in combination with short-duration storage (Figure 2), to obtain reliable, least-cost systems. The observed cost reductions between these various least-cost systems were dominated by a decrease in the installed wind capacity.

At current costs, least-cost CONUS systems that used hydrogen energy storage alone or in combination with other storage technologies resulted in the lowest total system costs and constituted a ∼72% reduction in total system costs as compared to the least-cost 100% reliable system that had only wind and solar generation without storage (Figure 2).

### Regional Storage Portfolios

**Figure 3:**
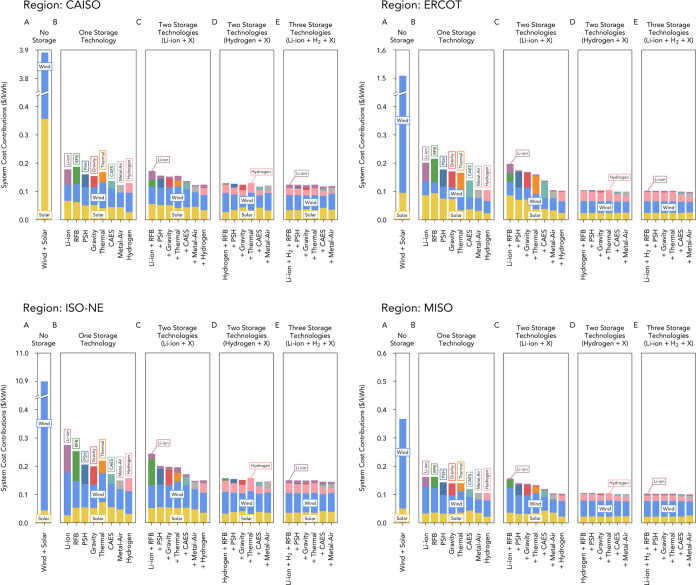
System costs for combinations of short-, mid-, and long-duration storage for four subnational independent system operator (ISO) geographic regions (CAISO, ERCOT, ISO-NE, and MISO). Tables S7–S10 support this figure. System costs when: (A) no storage technologies were deployed, (B) only one storage technology was available: Li-ion batteries, redox-flow batteries (RFB), pumped-storage hydropower (PSH), gravity energy storage, thermal energy storage, compressed air energy storage (CAES), metal–air battery storage, or hydrogen energy storage. (C) Two storage technologies were available: Li-ion batteries, with the second storage technology being a mid-duration storage technology or hydrogen energy storage. (D) Two storage technologies were available: hydrogen energy storage, with the second storage technology being a mid-duration storage technology. (E) Three storage technologies were available: Li-ion batteries and hydrogen energy storage, with the third storage technology being a mid-duration storage technology.

In ISO regions with high wind energy potential (e.g., MISO), long-duration energy storage resulted in the lowest system costs and thus had a higher value than short-duration storage. These trends were also observed in regions with high solar resources (e.g., CAISO), although the difference in added value between the two storage types was less pronounced than in regions with high wind resources (Figure 3, Tables S2 and S7). The cost reductions in all regions considered were associated with a substantial decrease in the wind generation capacity, as well as with a comparatively smaller reduction in the solar generation capacity. Long-duration storage compensated effectively for the seasonal variability and discharge needs associated with wind and solar generation in CONUS and regional ISOs and thus produced the lowest system costs and highest value of any storage technology.

Furthermore, at current technology costs, for all regions analyzed, the costs of systems that used long-duration storage were not affected substantially by additionally including in the system a short-duration storage technology (rightmost bar of Figure 2B vs rightmost bar of Figure 2C), a mid-duration storage technology (Figure 2D), or by including both mid-duration and short-duration storage technologies (Figure 2E). When both short- and long-duration storage (Figure 2E) technologies were available, only a very modest additional reduction in system costs (∼1%) was observed when CAES or metal–air batteries were used as the third storage technology (in all other cases in Figure 2E, a third storage technology was not deployed alongside short- and long-duration storage). These trends persisted even when using lower costs for wind and solar generation as predicted for the year 2050 by the NREL ATB report and substantially lower Li-ion battery costs (Figures S4 and S5). Hydrogen energy storage produced the lowest system cost in regions with high wind resources (MISO, ERCOT, and CONUS), whereas metal–air batteries produced the lowest system cost in regions with low wind resources (CAISO and ISO-NE) (Figure 3B, Tables S2 and S7).

### Time Scale over Which Storage Technologies Store Energy

Figures 4 and S6–S35 show that different storage technologies optimally stored energy on different time scales, and these time scales depended on which other storage technologies were also available for use in the electricity system, as well as regional geophysical resource variability. The time scale over which storage technologies stored energy was quantified by their “optimized discharge times” and “equivalent annual discharge cycles”. The “optimized discharge time (hours)” of each mid-duration storage technology was defined by its ratio of energy to power capacity in the least-cost system (Table S5). Furthermore, the “equivalent annual discharge cycles (cycles/year)” of each mid-duration storage technology was calculated by its total annual storage discharge divided by the deployed usable energy capacity of that type of storage in the least-cost system (Table S6).

**Figure 4:**
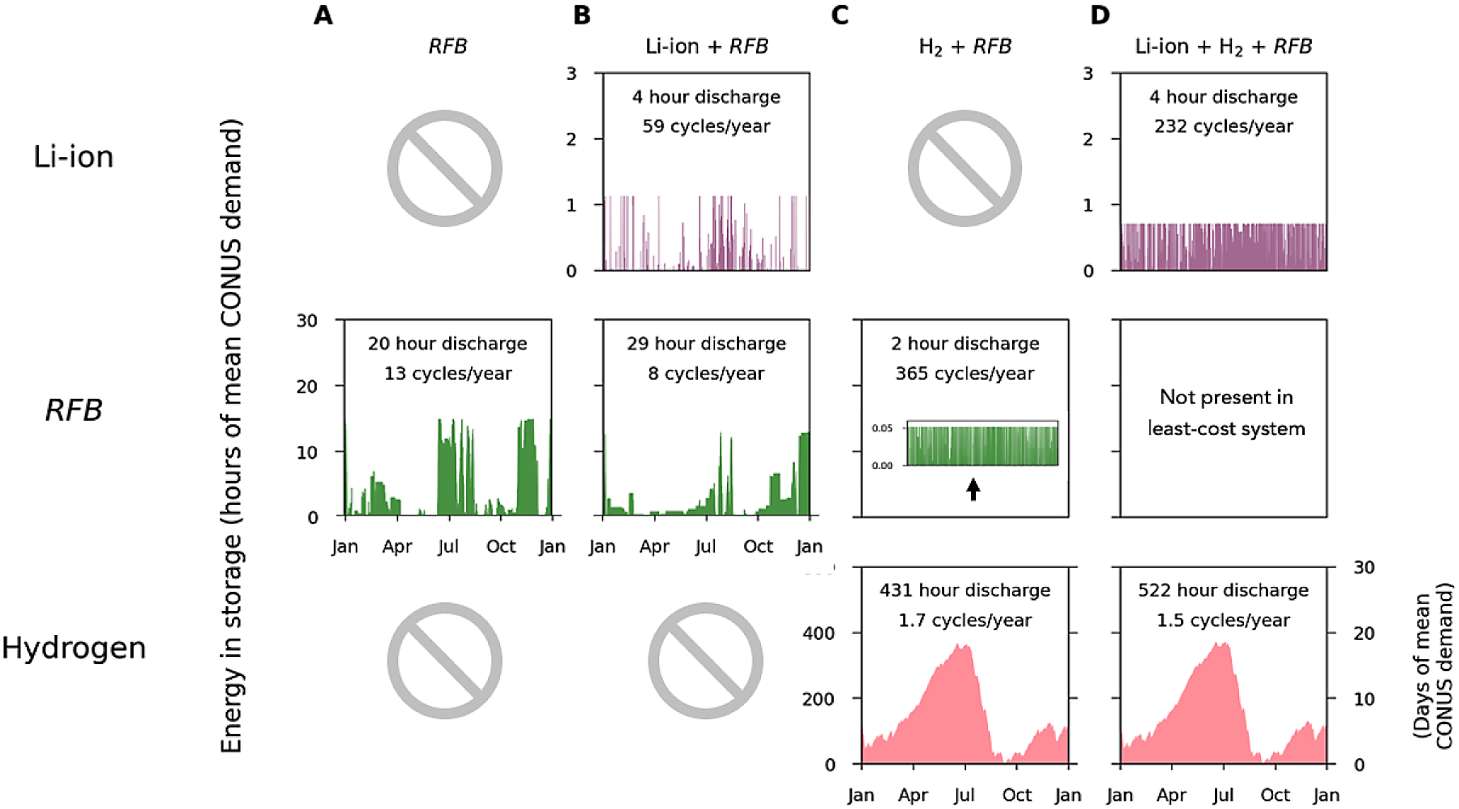
Energy in storage over one year for combinations of short-, mid-, and long-duration storage. The role (optimized discharge time) of mid-duration storage technologies (here represented by redox-flow batteries, RFB) depended on the availability of short- and long-duration storage. Figures S9–S38 show analogous results for regional indepedent system operators and other mid-duration energy storage technologies. Energy in storage over one year when: (A) RFB was the only storage technology. (B) RFB had lower power costs than Li-ion batteries and thus acted as short-duration storage. (C) RFB had lower energy costs than electrolytic hydrogen and thus acted as long-duration storage. (D) RFB was not present in the least-cost system, because less expensive short- and long-duration storage technologies were available.

When only Li-ion batteries and a mid-duration storage option were available, mid-duration storage options with unconstrained durations had optimal discharge times from 29 to 74 h. These discharge times emphasize the value of longer-duration energy storage to compensate for the seasonal variability of wind and solar resources, and thereby minimize the need for wind and/or solar generation capacity.

However, when only hydrogen energy storage and a mid-duration storage option were available, mid-duration storage options with unconstrained durations instead had optimal discharge times of under 11 h. In such least-cost systems, the mid-duration storage assets acted as a shorter-duration storage technology to compensate for the short-term variability of solar and wind resources, in conjunction with the electrolytic hydrogen that provided long-duration storage.

For example, when used in conjunction with short-duration storage, RFB had a discharge time of 29 h (Figure 4B), filled a longer-duration storage role, and provided value by cost-effectively compensating for the seasonal variability of wind resources. However, when used in conjunction with long-duration storage, RFB had a discharge time of 1.8 h (Figure 4C) and thus filled a shorter-duration storage role. At current costs, when both short- and long-duration storage options were installed, the least-cost system did not deploy RFB because demand was more cost-effectively met by the use of Li-ion batteries to compensate for short-term variability and by hydrogen energy storage to compensate for long-term weather and seasonal variability (Figure 4D). Analogous results for the energy in storage over one year of the remaining mid-duration storage technologies are presented in Figures S6–S35.

Although metal–air batteries were constrained to a duration of 100 h in all simulations, the energy dispatch from metal–air batteries reflects behavior similar to that described above for other mid-duration and long-duration storage technologies. Metal–air batteries cycled 7 times per year when used in addition to Li-ion batteries, and cycled 14 times per year when used in addition to hydrogen energy storage.

### Hypothetical *Storage X*: Parameterized Energy- and Power-Capacity Costs

**Figure 5:**
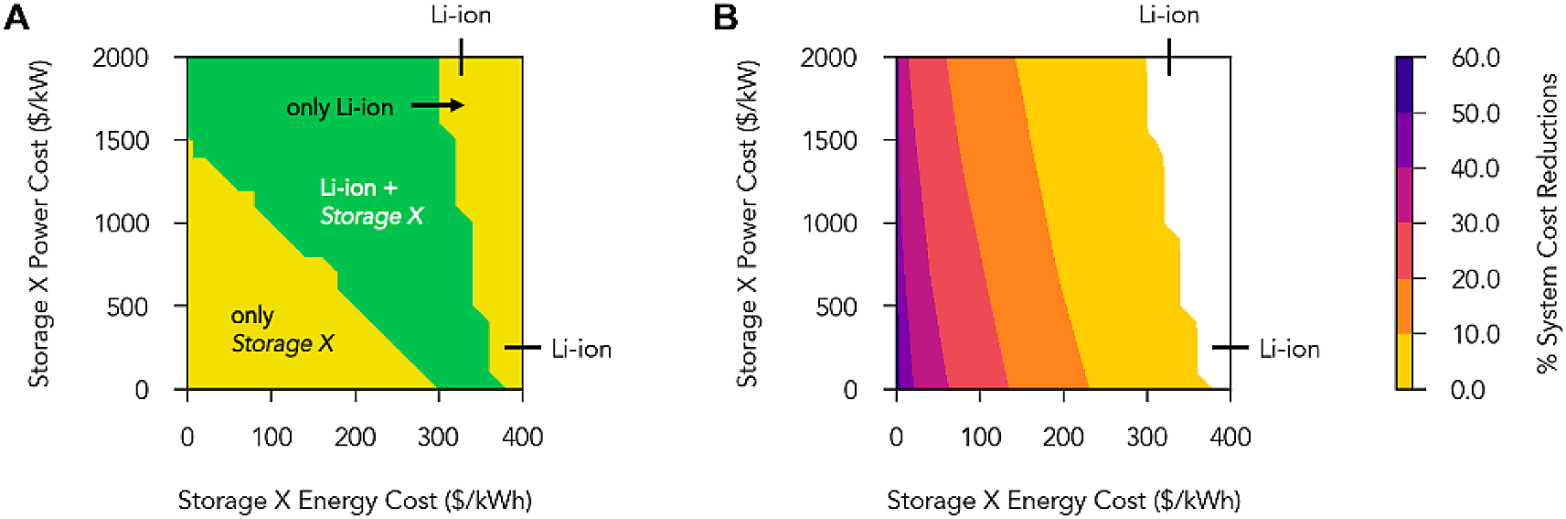
Storage technologies present and system cost reductions in scenarios with up to two storage options available: short-duration storage (Li-ion) and a hypothetical *Storage X* technology with energy- and power-capacity costs parameterized across wide ranges. Modeling parameters for Li-ion batteries were kept constant at base-case values, with Li-ion battery energy- and power-capacity costs marked on the top and right sides of the plot and numerical values in Table S4. Note that the energy- and power-capacity ratio (duration) of Li-ion batteries was fixed at 4 h. The round-trip efficiency of *Storage X* was fixed at 86%, to match the round-trip efficiency of Li-ion batteries. (A) Types of storage technologies used in 100% reliable least-cost systems in which *Storage X* energy- and power-capacity costs were varied across wide ranges. The technologies that were present in each parameter range are written in black and white fonts. (B) Percent reductions in total system cost as compared to a least-cost system with only Li-ion battery storage at base case costs. When *Storage X* energy-capacity costs were high, Li-ion batteries were the only storage technology deployed. When *Storage X* energy-capacity costs decreased, *Storage X* was deployed with Li-ion batteries. *Storage X* was deployed instead of Li-ion batteries when *Storage X* costs decreased below the diagonal line that connects a power-capacity cost of ∼1500 $/kW on the *y*-axis, and an energy-capacity cost of ∼300 $/kW h on the *x*-axis, reflective of the true cost of Li-ion batteries (Figure S1).

**Figure 6:**
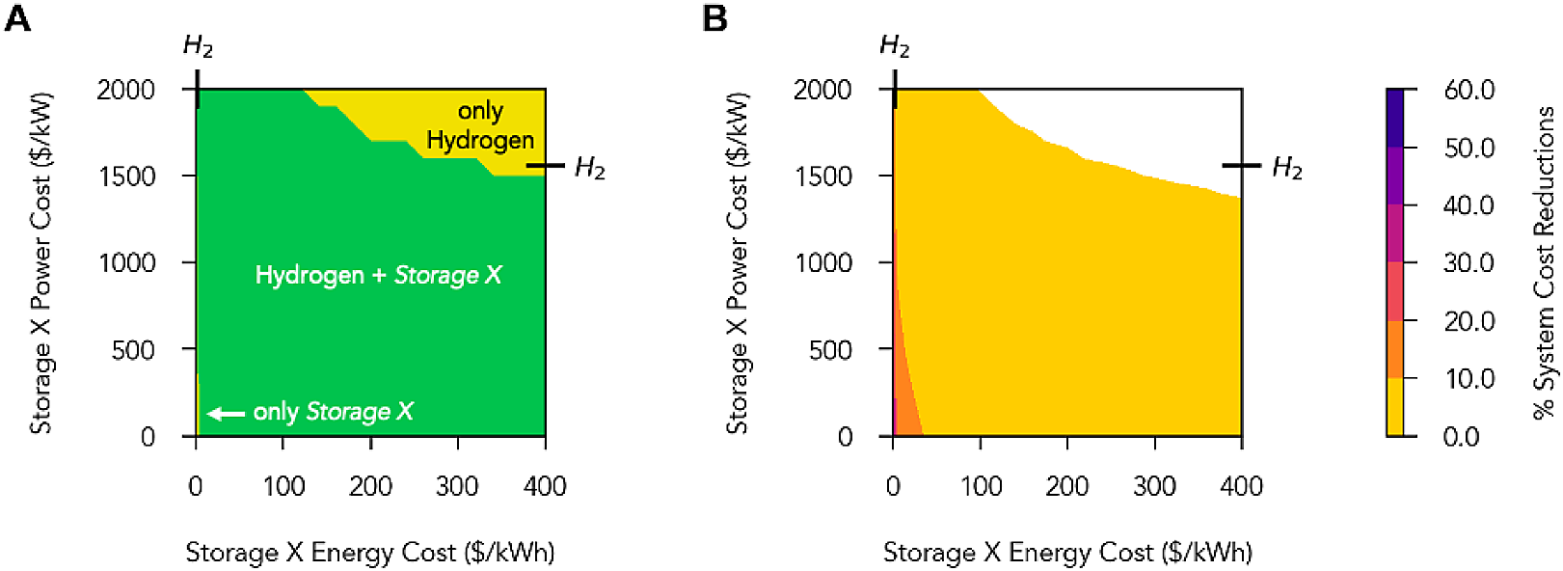
Storage technologies present and system cost reductions in scenarios with up to two storage options available: long-duration storage (hydrogen) and a hypothetical *Storage X* technology with energy- and power-capacity costs parameterized across wide ranges. Modeling parameters for hydrogen storage were fixed at base-case values, with hydrogen storage energy- and power-capacity costs marked on the top and right sides of the plot, with exact numerical values presented in Table S4. The round-trip efficiency of *Storage X* was fixed at 86%, to match the round-trip efficiency of Li-ion batteries. (A) Types of storage technologies used in least-cost 100% reliable systems in which *Storage X* energy- and power-capacity costs are parameterized across wide ranges. The technologies that were present in each parameter range are written in black and white fonts. (B) Percent reductions in total system cost as compared to a least-cost system with only hydrogen storage at base case costs.

Figure 5 explores a wide expanse of least-cost systems with storage portfolios of 2 technologies: Li-ion batteries and a hypothetical *Storage X* technology. Figure 6 is analogous to Figure 5 but instead shows least-cost systems that contain both hydrogen energy storage at current costs and a hypothetical *Storage X* technology. The energy- and power-capacity costs of *Storage X* were parameterized across wide ranges, and the round-trip efficiency of *Storage X* was fixed at 86% (the same round-trip efficiency as the modeled Li-ion technology). Li-ion batteries and hydrogen energy storage were assumed to have base-case costs (top and right labels of plots in Figures 5 and 6, respectively).

Due to the interrelated energy and power capacities of Li-ion batteries, the inclusion of *Storage X* in addition to Li-ion batteries reduces system costs at much higher energy-/power-capacity costs than when *Storage X* is included in addition to hydrogen energy storage (Figure 5 vs Figure 6). Furthermore, the inclusion of *Storage X* in addition to Li-ion batteries leads to larger system cost reductions than when *Storage X* is included in addition to hydrogen energy storage because *Storage X* fulfills long-term storage needs that Li-ion batteries cannot cost-effectively provide.

In Figure 5, substantial system cost reductions were obtained as the energy-capacity costs of *Storage X* decreased below ∼300 $/kW h (comparable to the energy-capacity cost of Li-ion batteries). Compared to a system that used only Li-ion battery storage, system costs were reduced by >10% when *Storage X* energy-capacity costs were <∼200 $/kW h and were reduced by >50% when the energy-capacity costs of *Storage X* were <∼20 $/kW h. When the energy- and power-capacity costs of the *Storage X* technology decreased below the diagonal border between the green and lower yellow regions in Figure 5A, *Storage X* completely displaced Li-ion by providing a more cost-effective storage solution for short-term storage needs than Li-ion batteries at current costs (Figure S36). This diagonal border connects a power-capacity cost of ∼1500 $/kW on the *y*-axis and an energy-capacity cost of ∼300 $/kW h on the *x*-axis. These costs are aligned with the true energy-capacity and power-capacity costs of Li-ion batteries, which are a consequence of Li-ion batteries’ nonseparable energy and power capacities (described in Figure S3).

In Figure 6, for the vast majority of the parameter space explored, system costs were reduced by <10% relative to a least-cost system that used only hydrogen energy storage. System cost reductions exceeded 10% only when the energy-capacity costs of *Storage X* were <∼30 $/kW h (Figure S37 shows a zoomed-in version of Figure 6). At *Storage X* energy-capacity costs <∼5 $/kW h and *Storage X* power-capacity costs <∼1500 $/kW (costs comparable with hydrogen energy storage), total system cost reductions ranged from 30% to 60%, and *Storage X* provided cost-effective long-duration storage relative to hydrogen energy storage at current costs (Figure S38).

Figures S39 and S40 show results analogous to Figures 5 and 6 for regional ISO systems. In all load-balancing regions investigated, the addition of a hypothetical storage technology (*Storage X)* that could provide over 100 h of energy storage with a high round-trip efficiency did not lower system costs over a wide range of parameterized energy-capacity and power-capacity costs, except when *Storage X* provided lower energy costs than hydrogen storage and served as long-duration storage, or provided lower power-capacity costs than Li-ion batteries and served as short-duration storage (Figures S39 and S40).

## Discussion

The idealized least-cost electricity system models considered herein have generation provided solely by wind and solar energy along with various types of energy storage technologies. Furthermore, the simulations performed here are for greenfield systems optimized de novo to minimize cost, as opposed to a capacity expansion model with legacy assets in place and costs evolving during deployment due to learning and economies of scale. In these models, demand can be met in full either by increasing the capacity of solar and/or wind generation (and incurring curtailment as a consequence) and/or by deploying the appropriate storage capacity. In this study, we define the value of a storage technology as its ability to reduce total system costs as compared to wind- and solar-based systems that do not include storage technologies. By modeling a stylized energy system, we aim to provide intuition for understanding complex system dynamics.

### Value of Deploying Different Individual Storage Technologies

The value of different storage technologies depends on their ability to cost-effectively compensate for solar and wind resource gaps and thus reduce the quantity of solar and wind generation capacity needed to reliably meet electricity demand. Different storage technologies are advantageous for filling in resource gaps on different time scales, so the optimal storage portfolio depends on the geophysical variability of solar and wind resources as well as the variability of electricity demand in a given region.

In the stylized system evaluated herein, electricity generation is provided solely by available solar and wind resources across CONUS, with no constraints on the amount of generation capacity that can be deployed. In the absence of sufficient energy storage in solar- and wind-based systems, electricity demand at night must be met by an appropriate amount of wind generation capacity, even in periods of low wind resources. Thus, deployment of storage primarily reduced total system costs by decreasing the capacity of wind generation in reliable least-cost systems (Figures 2 and 3). In the stylized CONUS electricity system, as the wind generation capacity decrease, the gap between electricity generation and demand was primarily seasonal, because the decrease in wind resource availability across CONUS during the summer was accompanied by a rise in electricity demand associated with cooling needs. Thus, storage technologies with lower energy-capacity costs and unconstrained durations (i.e., independently adjustable energy and power capacities) are better suited to compensate for this long-term resource gap, and thus allow larger reductions in wind capacity that lead to lower total system costs.

In the cases we have considered, the representative short-duration storage technology (Li-ion batteries) provided the smallest reductions in wind generation capacity and thus produced the lowest total system cost reduction. The impact of short-duration storage technologies on reducing total system costs is primarily due to their competitive power-conversion costs and high round-trip efficiency (Figures 2 and 3). Due to their 4 h duration, Li-ion batteries provide cost-effective short-duration storage for resource variability on the time scale of a few hours, but are less well-suited for cost-effectively addressing generation variability on longer time scales.

All mid- and long-duration storage technologies modeled had lower energy-capacity costs than Li-ion batteries and unconstrained durations (except for metal–air batteries, which were constrained to a 100 h duration). Consequently, these storage technologies cost-effectively provided long-duration storage (large energy-to-power ratios) to compensate for long-term wind and solar resource gaps. The storage technology with the lowest energy-capacity cost, hydrogen energy storage (at current costs), produced the largest reduction in system costs as a consequence of facilitating the highest decrease in wind capacity in reliable, least-cost electricity systems over CONUS. Similarly, other storage technologies decreased system costs primarily in accord with the trend in energy-capacity costs (Figures 2B and 3B), with some deviations from this pattern due to different power-capacity costs or round-trip efficiencies. Although metal–air batteries were constrained to a duration of 100 h, their total capacity costs are reported to be as low as 20 $/kW h and modeled as such in this study (Table S4).^34^ Consequently, metal–air batteries may be able to cost-effectively compensate for resource variability on time scales of up to 100 h (Figures S31–S35).

### Value of Simultaneously Deploying Multiple Storage Technologies

To realize further cost reductions in least-cost greenfield systems that use multiple storage technologies, additional storage technologies must have an advantage in some performance characteristic that allows for more cost-effective compensation for solar and wind resource gaps than other storage technologies that could be deployed in the system.

In the specific system explored in this paper, the inclusion of mid- or long-duration storage as a second storage technology in conjunction with short-duration Li-ion battery storage led to substantial system cost reductions relative to a system that only used Li-ion battery storage (Figure 2). Thus, when used in addition to Li-ion batteries, mid- and long-duration storage technologies substantially reduced system costs by providing long-term storage that was not satisfied cost-effectively by Li-ion battery storage.

Conversely, total system costs were not reduced substantially when Li-ion batteries were included as a second or third storage technology in addition to mid- and/or long-duration storage assets (Figure 2). Similarly, total costs were also not reduced substantially when mid-duration storage technologies were included in addition to long-duration storage assets (Figure 2). This behavior occurred because over CONUS, the resource supply vs demand gap is primarily long-term in nature. Once these long-term storage needs are addressed by a storage technology with the lowest energy-capacity costs in the system, shorter-term storage provided by other storage technologies with higher energy-capacity costs has a comparatively small impact on electricity system costs.

Furthermore, longer-duration storage technologies may serve a dual role by providing short-term storage with their existing power capacity, and make it more difficult for short-duration storage technologies to add value to the system (Figure S41). Although the modeled long-duration energy storage charge capacity is ∼20% of mean U.S. power demand, the long-duration energy storage discharge capacity is ∼90% of mean U.S. demand, allowing the long-duration storage assets also to provide peak demand requirements over a short time span (Figure S41, Table S7). Long-duration energy storage can cost-effectively be charged slowly over a long time period, but discharge power spikes needed to meet demand during weather events are large enough that the long-duration storage discharge capacity also fulfills short-term discharge requirements. This finding was general for CONUS as well as for the four U.S. load-balancing regions, regardless of whether the generation in each region was predominantly derived from wind or solar resources (Tables S7–S10).

For the stylized CONUS electricity system, a hypothetical second storage technology (*Storage X*) included in addition to hydrogen storage thus provides substantial system cost reductions only if the second technology has energy-capacity costs close to or lower than those of hydrogen energy storage. If this second storage technology has lower energy-capacity costs than hydrogen energy storage, it will replace hydrogen energy storage as the most cost-effective long-duration storage technology (Figures 6 and S37). This relationship was also observed when a third storage technology was included in addition to both hydrogen and Li-ion battery storage (Figures S42 and S43). However, when the round-trip efficiency of this hypothetical storage technology is low (36%), the storage technology must have even lower energy- and power-capacity costs to decrease total system costs (Figure S44).

In a parameterized analysis, we model hydrogen energy storage with 36% round-trip efficiency and *Li-ion battery storage* with 86% round-trip efficiency (Figures 5, 6, S39, S40, S42, and S43). The addition of a third storage technology such as mid-duration storage that could provide over 100 h of energy storage with a high round-trip efficiency did not lower system costs over a wide range of parameterized energy-capacity and power-capacity costs, except when the third storage technology provided lower energy costs than hydrogen storage and served as long-duration storage, or provided lower power-capacity costs than Li-ion batteries and served as short-duration storage (Figures S42 and S43). This behavior reflects the high value of long-duration storage with low energy-capacity costs to meet demand in full over a year of seasonal and weather-related resource variability, thereby minimizing curtailed generation, despite the low round-trip efficiency of hydrogen storage relative to batteries and *Storage X*.

### Influence of Regional Geophysical Resource Variability on the Value of Storage Technologies

The value of different storage technologies in a given electricity system directly results from the temporal relationships between wind and solar availability and/or electricity demand over the region of interest. Areas with different availability and variability of wind and solar resources and a different profile of electricity demand will thus have characteristic storage requirements on different time scales than those intrinsic to CONUS as a whole (Table S2). The value of long-duration storage relative to short-duration storage was higher in regions that were more dependent on wind generation (e.g., MISO relative to CAISO) (Figure 3, Table S2, Table S7).

When ample wind capacity is available, the relationship between wind generation and electricity demand is especially influential in determining the most cost-effective storage technology portfolios in electricity systems based on solar and wind generation. Without energy storage or firm dispatchable energy, total system costs are primarily driven by the needed wind generation capacity to compensate for a lack of solar generation at night (Figures 2 and 3). Storage technologies that most cost-effectively reduce the needed wind generation capacity by compensating for gaps between wind generation and electricity demand are thus the most advantageous for reducing total system costs. Hence, the shape of the resource gap between wind generation and electricity demand determines whether short- or long-duration storage is more cost-effective in the load-balancing region of concern.

In the four load-balancing regions, when only one storage technology was considered, deployment of long-duration energy storage produced the lowest electricity system costs due to their low energy-capacity costs (∼2 $/kW h for hydrogen energy storage and a total cost of 20 $/kW h for metal–air batteries, Table S4). Additionally, this conclusion was generally valid for all of the different load-balancing regions, despite the least-cost system being dominated by wind generation in MISO and ERCOT and by solar generation in CAISO (Table S2). Moreover, in all load-balancing regions investigated, the least-cost systems that included hydrogen energy storage had sufficient energy and power capacity to also meet short-duration energy and power storage needs, so the addition of short-duration storage as a second storage technology did not markedly reduce total system costs when long-duration storage was available.

### Model Architecture Changes

Long-duration energy storage satisfied short-term storage needs in four regional ISO systems. Here, we discuss specific scenarios and model architecture changes that would change our key findings. For example, a region near the equator powered by solar generation with low seasonal variability and limited wind capacity would have storage needs that are primarily short-term and thus benefit far more from using a short-duration storage technology as opposed to a long-duration storage technology. Figure S45 presents CONUS systems with only solar generation.

When firm dispatchable generators are available, the storage capacity required in 100% reliable, least-cost systems is decreased substantially (Figure S46), compared to systems based on solar and wind generation with storage technologies.^33^ When firm inflexible generators are available for base-load power, our main findings still pertain to the amount of energy provided by the variable wind/solar generation that remains. Furthermore, smaller geographic regions within CONUS required a larger storage capacity per unit demand compared to CONUS itself, due to the larger variability of wind and solar resources in smaller regions (Figures 2 and 3, Tables S7–S10).

We consider only the role of storage technologies in grid-scale bulk storage services for electricity sector balancing, and not in other energy storage services such as ancillary, or transmission and distribution infrastructure services. Furthermore, our electricity system model considers a specified electricity demand time series and does not explicitly model end uses or demand flexibility. Demand flexibility is a “fast-burst” balancing resource and is expected to lessen the need for short-duration energy storage relative to the need for long-duration energy storage.^13^ Including demand management in this model (a strategy for intraday weather events, but not seasonal storage) would consequently reduce the value of short-duration energy storage and reinforce our findings regarding the value of long-duration energy storage.

Our model assumes cost-optimal allocation of technology assets and lossless transmission of electricity across CONUS and subnational ISO regions. In real-world scenarios with congested or geographically constrained transmission lines, these limitations imply the need for greater energy storage capacities than those obtained from this stylized macro-energy model (Figures 2 and 3, Tables S7–S10). Similarly, we assume that least-cost greenfield systems are built *de novo* and are not along a transition path or a capacity expansion approach. When legacy assets are in place or a transition capacity expansion path approach is used, legacy solar generation may allow for more value from short- and even mid-duration storage than is obtained from the set of assumptions in our model.

The actual deployed capacity of storage technologies will be constrained by geographic, legal, material, political, and social considerations that have not been included in our model. For example, PSH requires geographic areas with elevated locations for water reservoirs and water access that may be difficult to secure due to competing demands for freshwater from agricultural, industrial, and household interests. Similarly, both CAES and hydrogen storage can advantageously use underground salt caverns for energy storage.^35,36^ Hydrogen fuel has a higher energy density and lower energy-capacity costs than compressed air, but the choice between CAES and hydrogen storage for geologically constrained space in underground storage reservoirs is site-specific.^37^ Other energy storage technologies, such as Li-ion batteries, redox-flow batteries, gravitational energy storage, thermal energy storage, and metal–air battery storage, require space for building facilities that may be scarce in urban environments. Deployment of storage technologies also faces legal constraints, including permitting by local, state, and federal agencies.

Furthermore, fabrication of energy storage technologies requires materials that may involve potential supply chain constraints or other sociopolitical challenges. For example, PEM electrolyzers for hydrogen production currently require catalysts that contain platinum and iridium, among the scarcest nonradioactive elements on Earth.^38^ Lithium-ion batteries require cobalt (Co), which involves human rights concerns related to its extraction, as well as potential supply chain issues. These concerns and issues are due in large part to the geographical concentration of Co supply in the Democratic Republic of the Congo and Co refining in China.^35,36^ Vanadium (V) redox-flow batteries and iron–air batteries require V and iron, and increased mining of these minerals may lead to environmental, social, and supply chain concerns.

### Long-Duration Storage May Satisfy Short-Term Storage Needs

We find generalizable results that may advise the assembly of optimal energy storage portfolios in systems that rely on wind and solar generation. We find that the optimal storage portfolio depends on the time scales of storage needs for a given wind/solar-based system. Additional hypothetical storage technologies can compete with Li-ion batteries over a wide range of energy- and power-capacity costs, but can compete with hydrogen storage only at very low energy-capacity costs. Least-cost systems contained sufficient power capacities of long-duration storage also to meet short-term power needs, so that the addition of short-duration storage did not markedly reduce total system costs.

## Data Availability

In the interest of transparency and reproducibility, all model code, input data, and plotting code are publicly available at: https://zenodo.org/doi/10.5281/zenodo.10689478.^39^

## Supporting Information Available

Describing the model parametrization, costs, and presenting additional results; electricity sources, sinks, and storage technologies within the macroscale energy model (Figure S1); base case costs and efficiencies assumed for the short-, mid-, and long-duration storage technologies considered in this study (Figure S2); base case energy- and power-capacity total overnight costs of energy storage technologies modeled, where Li-ion total costs are shown as a dashed line (Figure S3); CONUS system costs for combinations of short-, mid-, and long-duration storage using predicted wind and solar costs for the year 2050 (Figure S4); if the cost of Li-ion batteries were much lower than current costs, deployment of Li-ion batteries would be much more effective for reducing total system costs, and would replace utilization of two mid-duration storage technologies (gravity energy storage and PSH) as compared to the base case (Figure S5); role of redox-flow battery (RFB) energy storage in CONUS (Figure S6), CAISO (Figure S7), ERCOT (Figure S8), ISO-NE (Figure S9), MISO (Figure S10) systems, role of pumped-storage hydropower (PSH) energy storage in CONUS (Figure S11), CAISO (Figure S12), ERCOT (Figure S13), ISO-NE (Figure S14), MISO (Figure S15) systems, role of gravity energy storage in CONUS (Figure S16), CAISO (Figure S17), ERCOT (Figure S18), ISO-NE (Figure S19), MISO (Figure S20) systems, role of thermal energy storage in CONUS (Figure S21), CAISO (Figure S22), ERCOT (Figure S23), ISO-NE (Figure S24), MISO (Figure S25) systems, role of compressed-air energy storage (CAES) in CONUS (Figure S26), CAISO (Figure S27), ERCOT (Figure S28), ISO-NE (Figure S29), MISO (Figure S30) systems, and role of metal–air battery energy storage in CONUS (Figure S31), CAISO (Figure S32), ERCOT (Figure S33), ISO-NE (Figure S34), MISO (Figure S35) systems with different combinations of short-, mid-, and long-duration storage; roles of storage technologies in least-cost CONUS systems with up to two storage options available (Figures S36 and S38); Figure 6 with the *x*-axes zoomed in to energy-capacity costs (Figure S37); storage technologies present in least-cost regional ISO systems and system cost reductions in systems with up to two storage options available (Figures S39 and S40); energy in storage over one year when hydrogen energy storage is the only storage technology available in a least-cost CONUS electricity system that relies on wind and solar generation (Figure S41); storage technologies present in least-cost CONUS systems and system cost reductions in systems with up to three storage options available (Figures S42 and S43); analogous plot to Figure S42 (Figure S44); the value of short-duration storage (Figure S45); when firm generators were available, the storage capacity required in least-cost systems decreased substantially (Figure S46); energy-capacity costs and power-capacity costs of energy storage technologies (Table S1); regional average wind and solar capacity factors for 2018 (Table S2); costs and assumptions for generation technologies (Table S3); base case costs and assumptions for storage technologies (Table S4); optimized discharge times (hours) (Table S5); equivalent annual discharge cycles (Table S6); least-cost system results when various single storage technologies are available (Table S7); least-cost system results when two storage technologies were available (Tables S8 and S9); least-cost system results when three storage technologies were available (Table S10); model nomenclature (Table S11) (PDF)
