# Supplementary material for: The Influence of Regional Geophysical Resource Variability on the Value of Single- and Multistorage Technology Portfolios
Source: Environ Sci Technol. 2024 Jul 15;58(30):13251–62. doi: 10.1021/acs.est.3c10188 (PMC11295120; doi:10.1021/acs.est.3c10188)
Supplement: Supplementary file 1 — es3c10188_si_001.pdf [file es3c10188_si_001.pdf]

The influence of regional geophysical resource variability on the value of single- and multi-storage technology portfolios

Anna X. Li [1+], Edgar Virgüez [2+*], Jacqueline A. Dowling [2+], Alicia Wongel [2], Dominic Covelli [1], Tyler H. Ruggles [2], Natasha Reich [1], Nathan S. Lewis [1,3*], Ken Caldeira [2,4*]*

[1] Division of Chemistry and Chemical Engineering, California Institute of Technology, Pasadena, California, 91125, United States.
[2] Department of Global Ecology, Carnegie Institution for Science, Stanford, California, 94305, United States.
[3] Beckman Institute, California Institute of Technology, Pasadena, California, 91125, United States.
[4] Gates Ventures LLC, Kirkland, Washington, 98033, United States.

+ A.X.L., E.V., and J.A.D. contributed equally to this paper (equally contributing first authors)

** Corresponding authors:*

- Anna Li (li.anna6389@gmail.com)
- Edgar Virgüez (evirguez@carnegiescience.edu)
- Nate Lewis (nslewis@caltech.edu)
- Ken Caldeira (kcaldeira@carnegiescience.edu)

The supplementary material for this study includes:

- Figures S1-S46 (pages S2 to S35)
- Tables S1-S11 (pages S36 to S76)

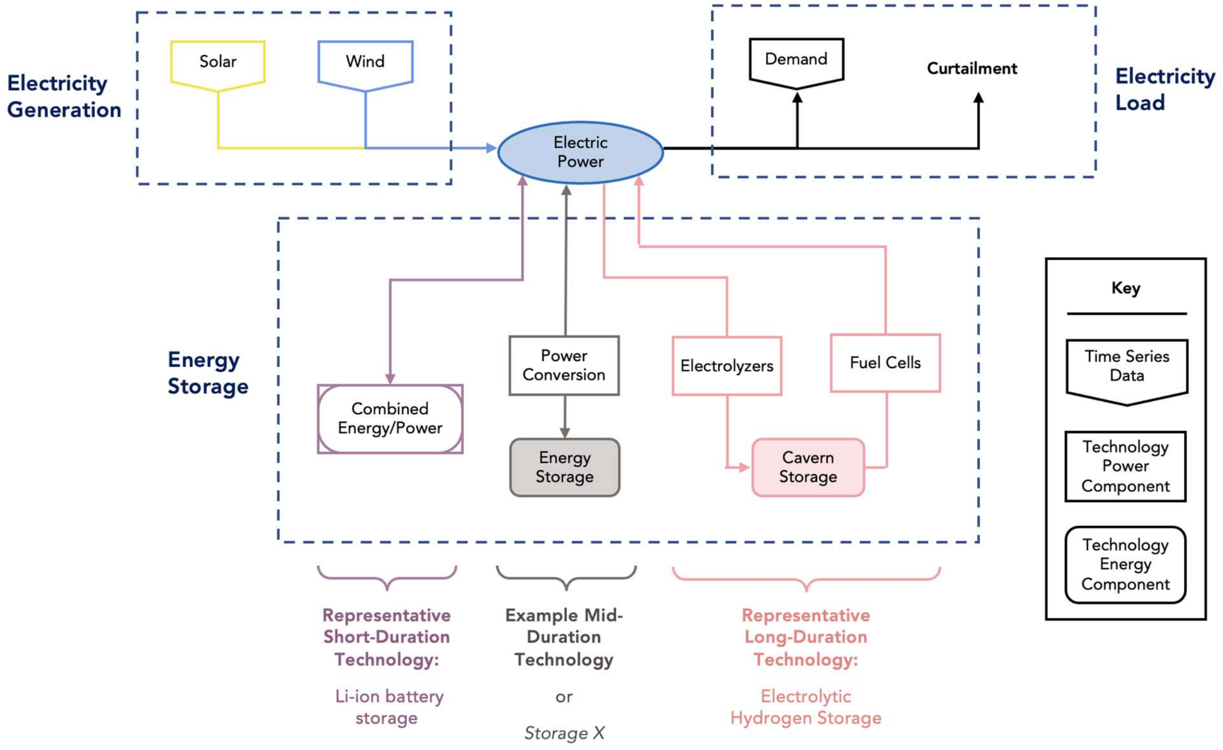

Figure S1. Electricity sources, sinks, and storage technologies within the macroscale energy model.

Arrows indicate the direction of electricity flow, and shaded shapes represent nodes in which electricity can be stored. Conventional Li-ion batteries (Li-ion) were modeled with combined energy and power components and were fixed to a duration of 4 h. *Storage X* was modeled with separate power- and energy-storage components. Electrolytic hydrogen storage was modeled with separate power- and energy-storage components, in which charging and discharging components could have different capacities.

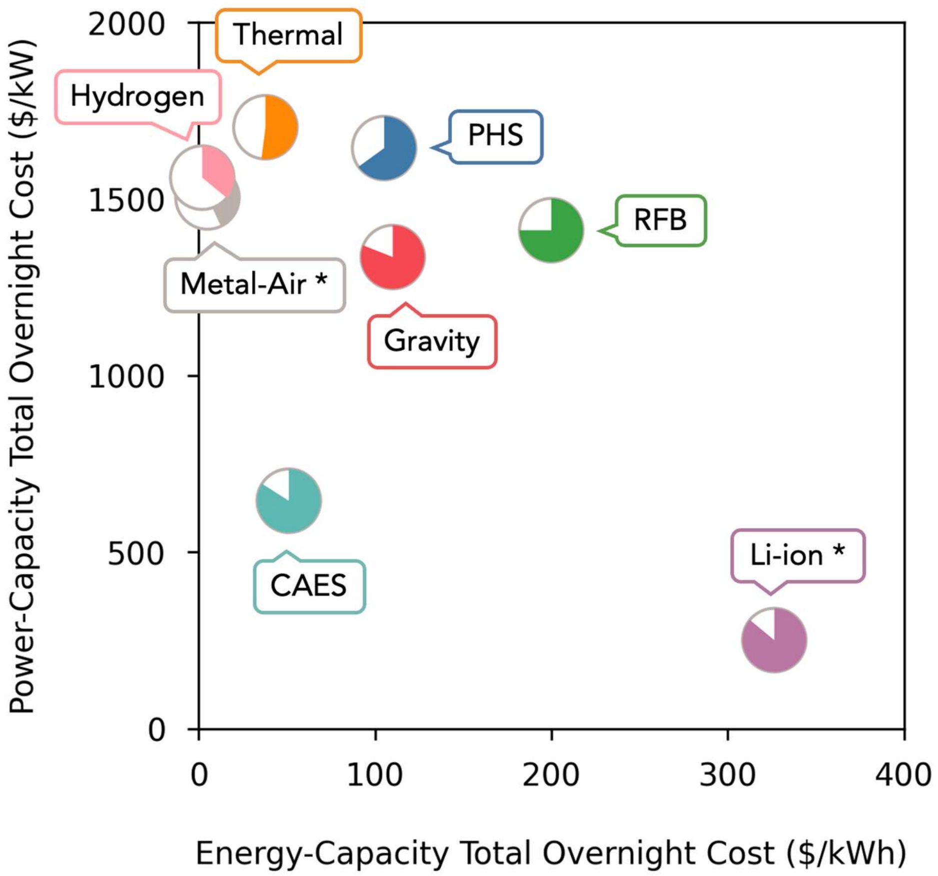

Figure S2. Base case costs and efficiencies assumed for the short-, mid-, and long-duration storage technologies considered in this study.

Base case energy-capacity costs, power-capacity costs, and round-trip efficiencies (designated by the colored area of pie charts) of different storage technologies modeled herein. In this work, electrolytic hydrogen was considered as a prototypical long-duration storage technology, Li-ion batteries provided a prototypical short-duration storage technology, and the other technologies were labeled as mid-duration storage technologies. Table S4 supports this figure. See the Methods section for additional base case cost assumption details.

*Individual Li-ion and metal-air batteries have energy- and power-capacities that cannot be independently sized. Thus, based on their fixed durations (energy- to power-capacity ratio) of 4 h and 100 h, respectively, their total costs are best described by the diagonal lines illustrated in Figure 1. For metal-air batteries, the energy- and power-capacity costs shown in this plot were calculated by splitting the total overnight cost used for modeling through the method described in Table S4.

† Because electrolytic hydrogen energy storage has separate technologies for charging and discharging, the power-capacity cost was calculated by assuming that 0.25 kW of charging capacity was installed for every 1 kW of discharging capacity, as was observed in solar- and wind-based least-cost systems with energy storage provided by both Li-ion batteries and hydrogen energy storage. CAES also has separate technologies for charging and discharging, and its power-capacity cost was calculated with the same ratio as that used for hydrogen energy storage.

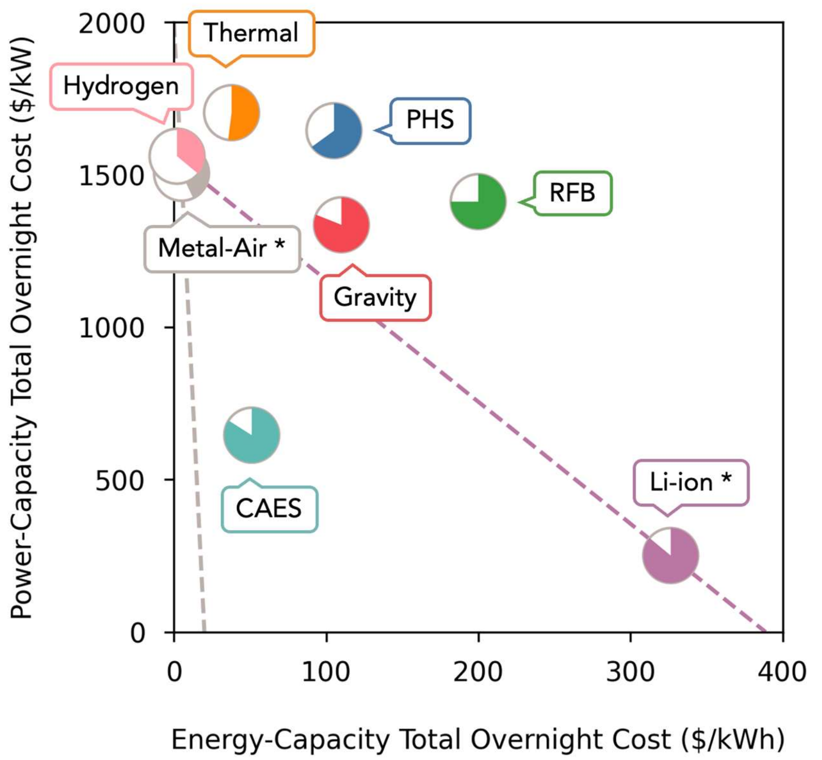

Figure S3. Base case energy- and power-capacity total overnight costs of energy storage technologies modeled, where Li-ion total costs are shown as a dashed line.

Although Li-ion costs have been divided by Hunter et al. into an energy-capacity cost of 326 $/kWh and power-capacity cost of 251 $/kW, individual Li-ion batteries have energy- and power- capacities that cannot be independently sized. Thus, for the 4-hour Li-ion battery considered here, if the battery is sized based on energy capacity, then the energy-capacity cost is effectively 326 $/kWh+251 $/kW4 h=388.75 $/kWh, and the power-capacity cost is effectively 0 $/kW. In contrast, if the battery is sized based on power capacity, then the energy-capacity cost is effectively 0 $/kW and the power-capacity cost is effectively 388.75 $/kWh4h=1555 $/kW. Thus, Li-ion battery costs are most accurately described by the dashed (purple) line connecting 388.75 $/kWh on the x-axis and 1555 $/kW on the y-axis.

Similarly, individual metal-air batteries have energy- and power-capacities that cannot be independently sized. We represent metal-air batteries based on Form Energy's claimed 100 h iron-air batteries that have a total cost of 20 $/kWh. Because we know only the total cost of these iron-air batteries, their energy and power-capacity costs are visualized by splitting this total cost based on the ratio of energy-capacity cost to power-capacity cost used for metal-air batteries.

For the 100h metal-air battery considered here, if the battery is sized based on energy capacity, then the energy-capacity cost is effectively 20 $/kWh, and the power-capacity cost is effectively 0 $/kW. In contrast, if the battery is sized based on power capacity, then the energy-capacity cost is effectively 0 $/kW and the power-capacity cost is effectively 20 $/kWh100 h=2000 $/kW. Thus, metal-air battery costs are most accurately described by the dashed (gray) line that connects 20 $/kWh on the x-axis and 2000 $/kW on the y-axis.

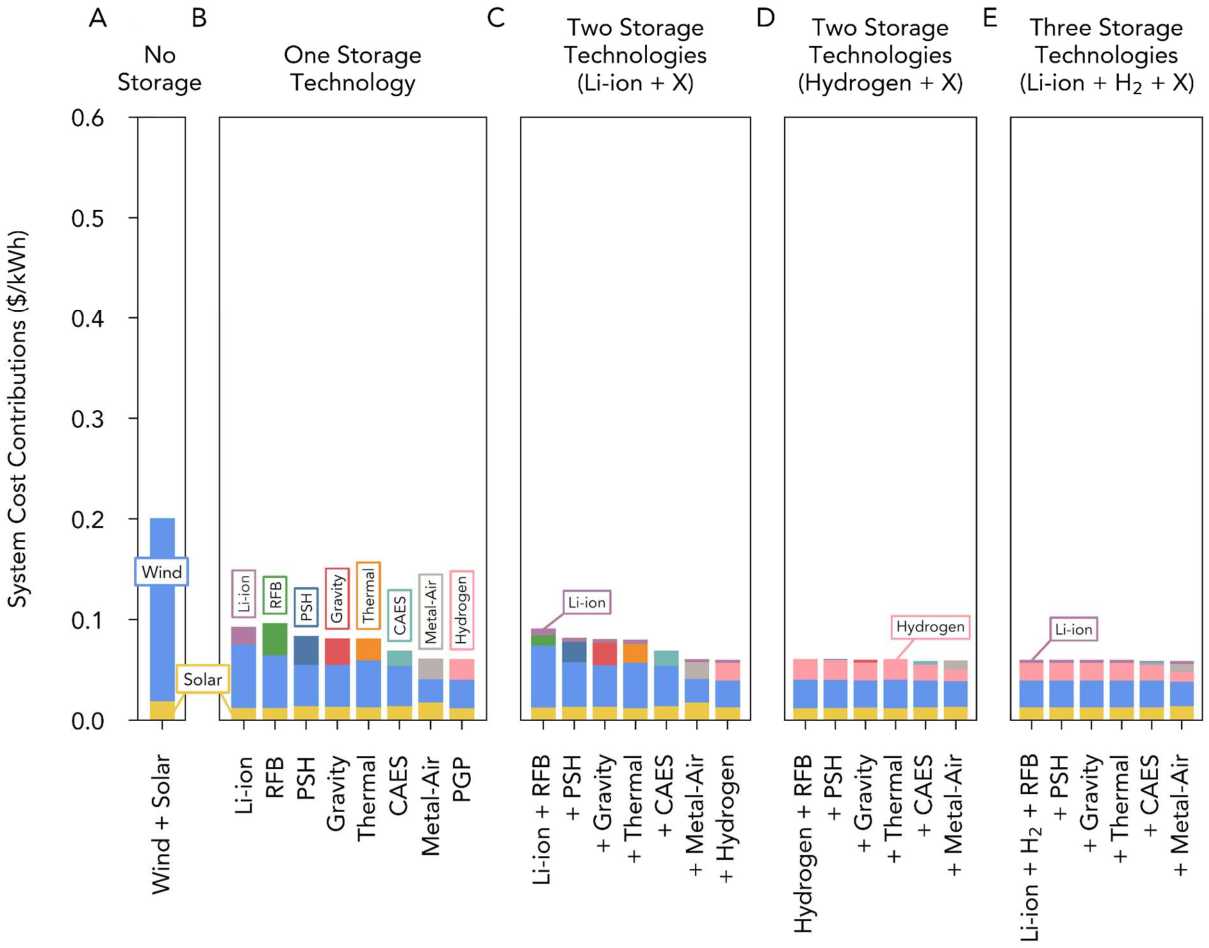

Figure S4. CONUS system costs for combinations of short-, mid-, and long-duration storage using wind and solar costs predicted for the year 2050.

Cost contributions of technologies in wind and solar generation-based systems with one, two, and three storage technologies. Wind and solar generation costs are the lower bound values for the "Moderate" scenario in the National Renewable Energy Laboratory (NREL)'s Annual Technology Baseline (ATB) report for the year 2050. System costs when:

(A) Only one storage technology was available: Li-ion batteries, *RFB* (redox-flow batteries), *Gravity* energy storage, *PSH* (Pumped-Storage Hydropower), *CAES* (Compressed Air Energy Storage), *Thermal* energy storage, *Metal-Air* battery storage, or hydrogen energy storage.

(B) Two storage technologies were available: Li-ion batteries, with the second storage technology being a mid-duration storage technology or hydrogen energy storage.

(C) Two storage technologies were available: Hydrogen energy storage, with the second storage technology being a mid-duration storage technology.

(D) Three storage technologies were available: Li-ion batteries and hydrogen energy storage, with the third storage technology being a mid-duration storage technology.

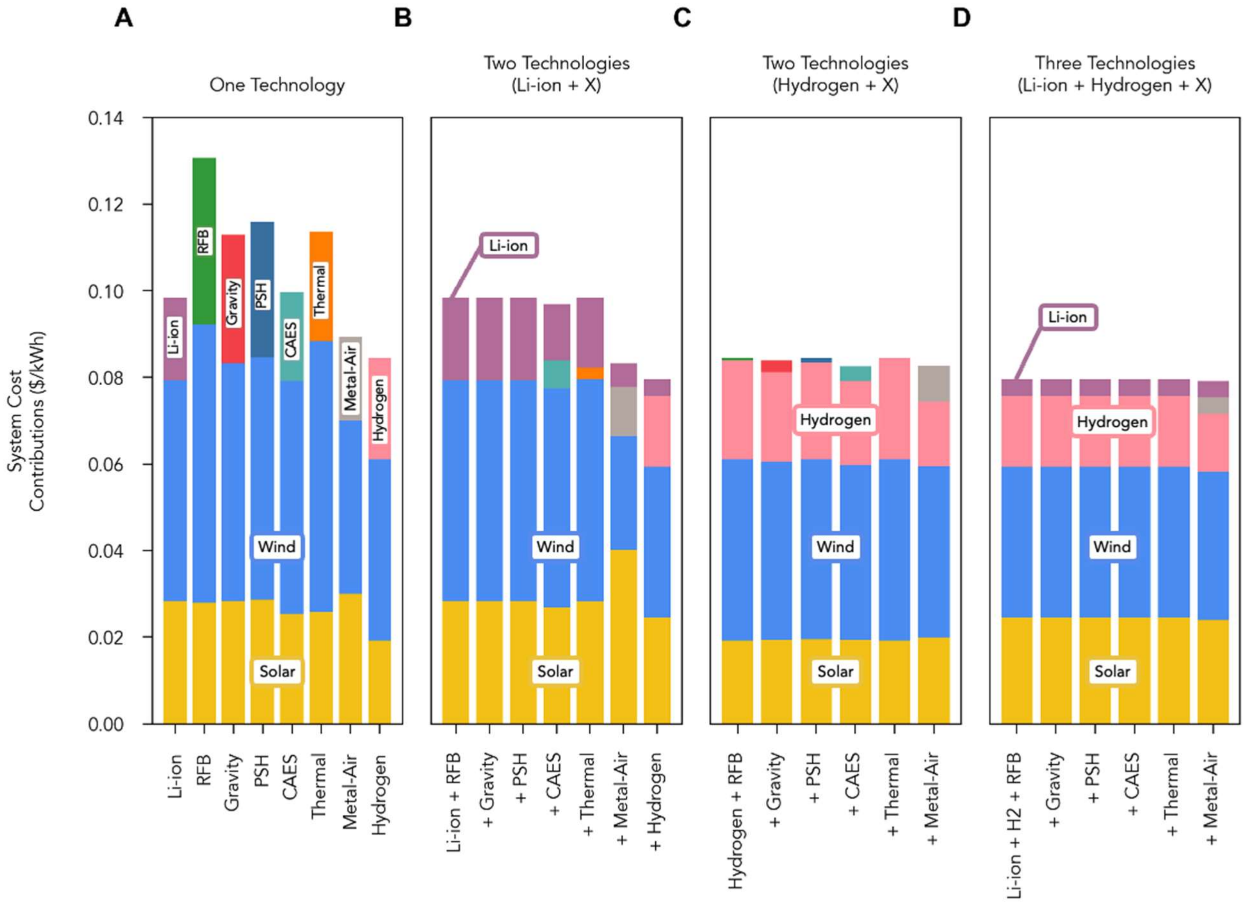

Figure S5. If the cost of Li-ion batteries were much lower than current costs, deployment of Li-ion batteries would be much more effective for reducing total system costs and would replace utilization of two mid-duration storage technologies (gravity energy storage and PSH) as compared to the base case.

Cost contributions of technologies in a wind and solar generation-based CONUS system, with one, two, and three storage technologies. The total cost of Li-ion batteries was 100 $/kWh, a four-fold decrease from the base case. Costs of all other technologies were kept at the base case values. System costs when:

(A) Only one storage technology was available: *Li-ion* batteries, *RFB* (redox-flow batteries), *Gravity* energy storage, *PSH* (Pumped-Storage Hydropower), *CAES* (Compressed Air Energy Storage), *Thermal* energy storage, *Metal-Air* battery storage, or *Hydrogen* energy storage.

(B) Two storage technologies were available: Li-ion batteries, with the second storage technology being a mid-duration storage technology or hydrogen energy storage.

(C) Two storage technologies were available: Hydrogen energy storage, with the second storage technology being a mid-duration storage technology.

(D) Three storage technologies were available: Li-ion batteries and hydrogen energy storage, with the third storage technology being a mid-duration storage technology.

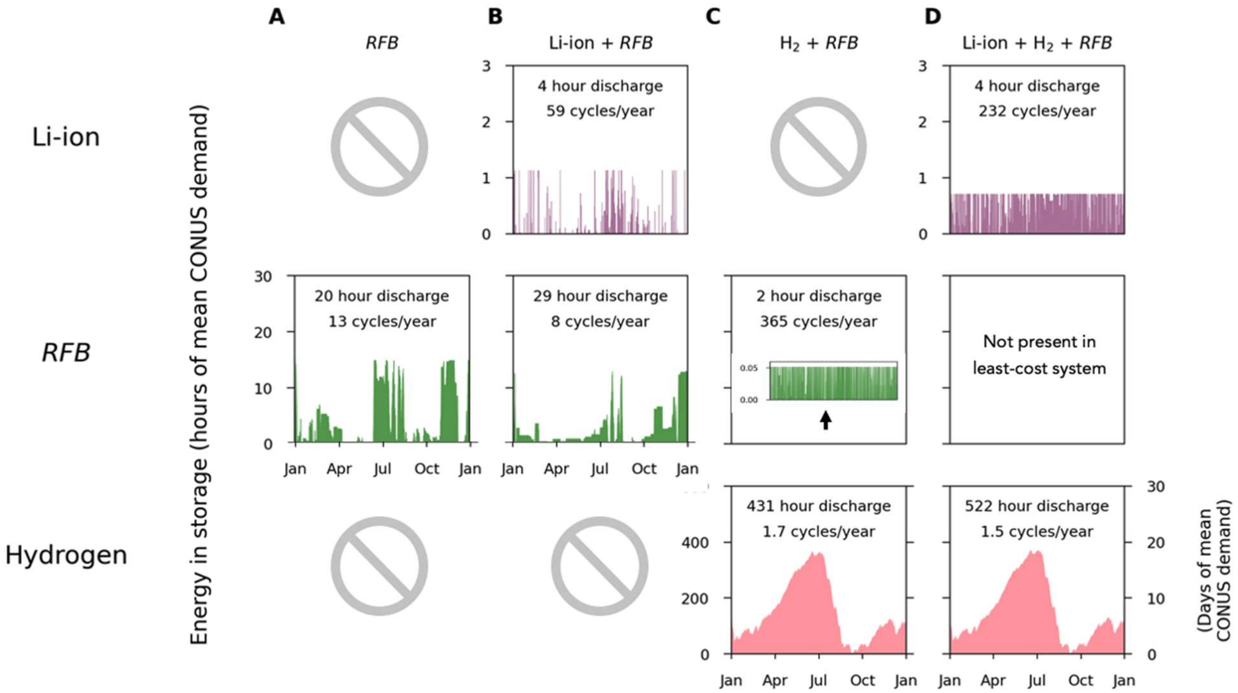

Figure S6. Role of redox-flow battery (RFB) energy storage in CONUS systems with different combinations of short-, mid-, and long-duration storage.

The role (optimized discharge time) of mid-duration storage technologies (here represented by redox-flow batteries, RFB) depended on the availability of short- and long-duration storage. Energy in storage over one year when:

(A) RFB was the only storage technology.
(B) RFB had lower power costs than Li-ion batteries and thus acted as short-duration storage.
(C) RFB had lower energy costs than electrolytic hydrogen and thus acted as long-duration storage.
(D) RFB was not present in the least-cost system, because less expensive short- and long-duration storage technologies were available.

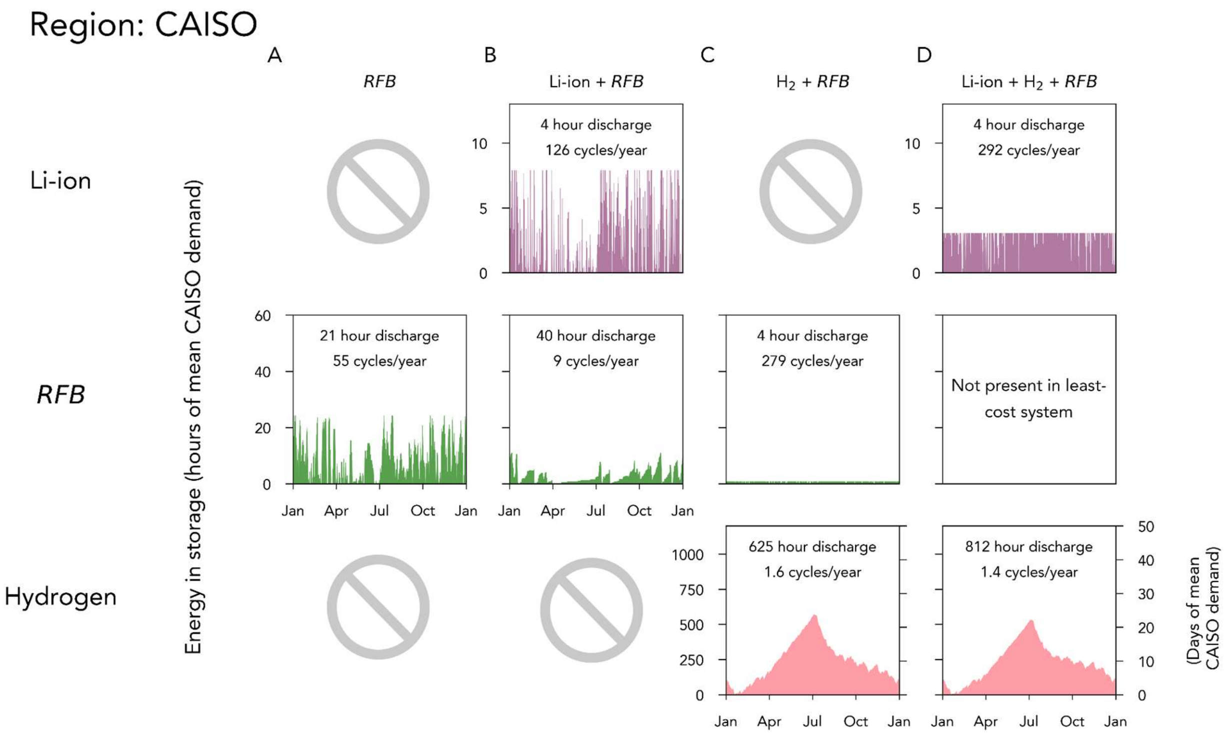

Figure S7. Role of redox-flow battery (RFB) energy storage in CAISO systems with different combinations of short-, mid-, and long-duration storage.

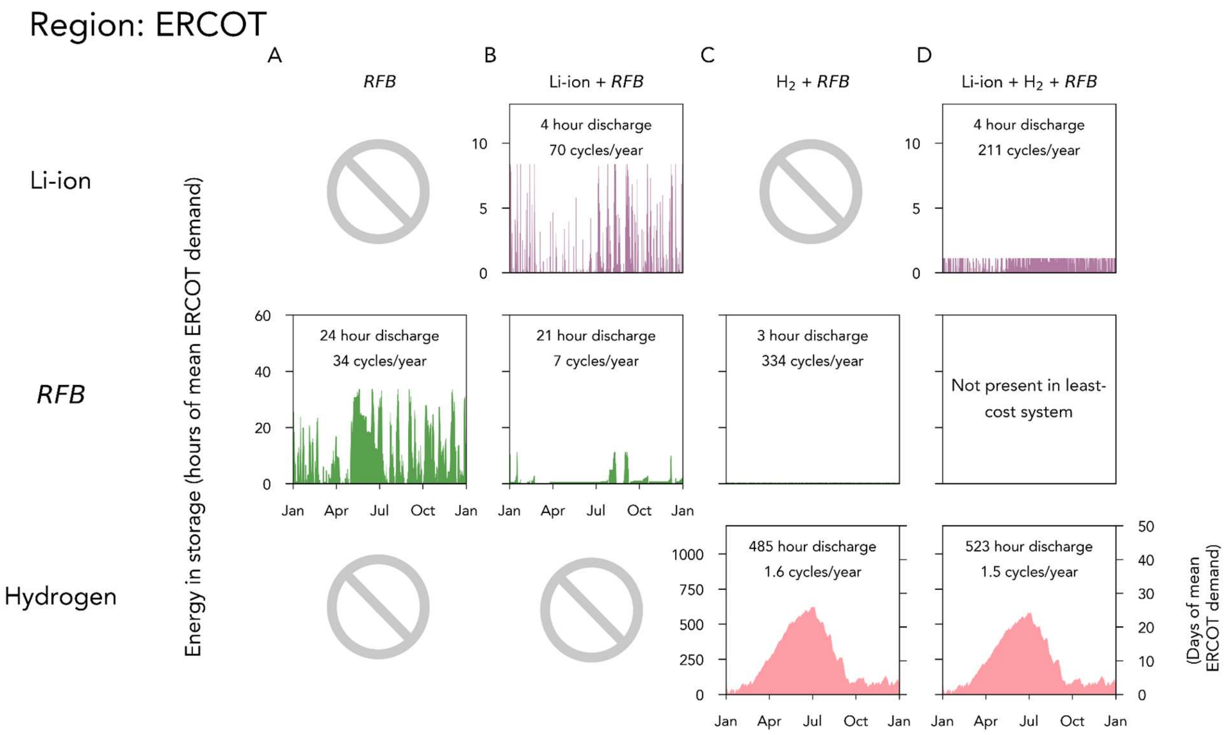

Figure S8. Role of redox-flow battery (RFB) energy storage in ERCOT systems with different combinations of short-, mid-, and long-duration storage.

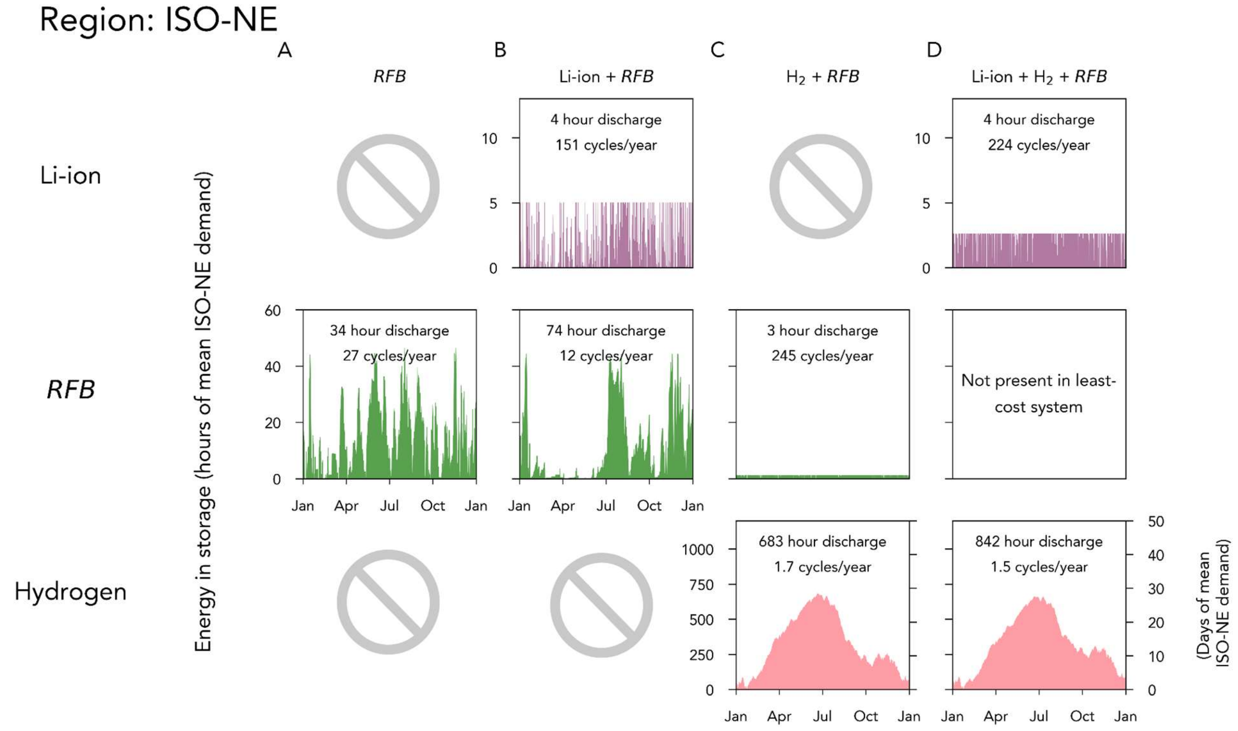

Figure S9. Role of redox-flow battery (RFB) energy storage in ISO-NE systems with different combinations of short-, mid-, and long-duration storage.

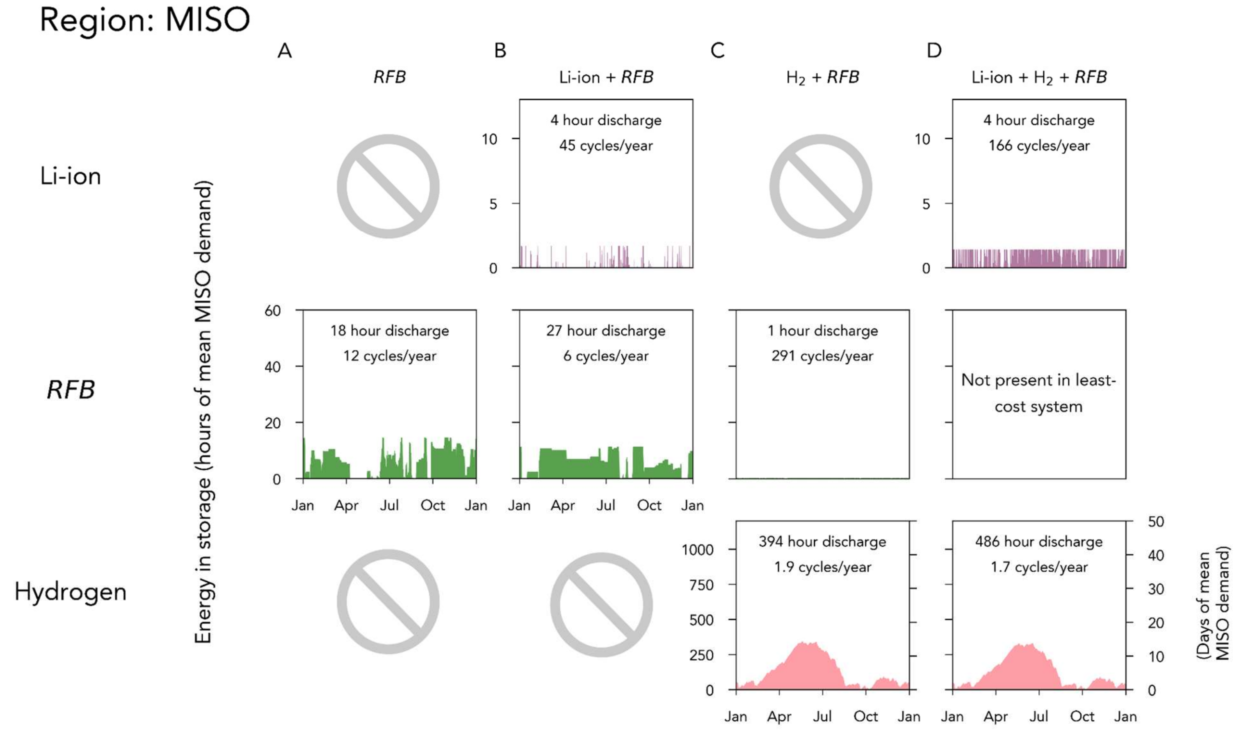

Figure S10. Role of redox-flow battery (RFB) energy storage in MISO systems with different combinations of short-, mid-, and long-duration storage.

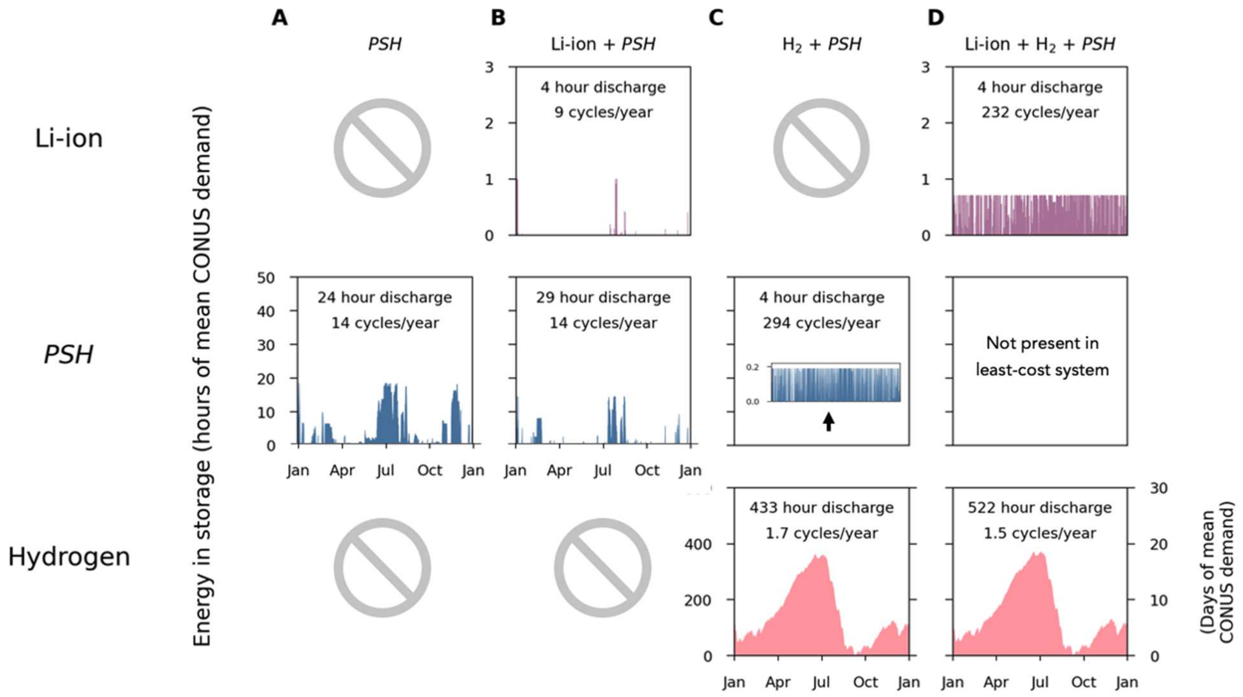

Figure S11. Role of pumped-storage hydropower (PSH) energy storage in CONUS systems with different combinations of short-, mid-, and long-duration storage.

The role (optimized discharge time) of mid-duration storage technologies (here represented by pumped-storage hydropower, PSH) depended on the availability of short- and long-duration storage. Energy in storage over one year when:

(A) PSH was the only storage technology.
(B) PSH competed with short-duration storage (Li-ion batteries).
(C) PSH competed with long-duration storage (electrolytic hydrogen).
(D) PSH was not present in the least-cost system when both short- and long-duration storage were available.

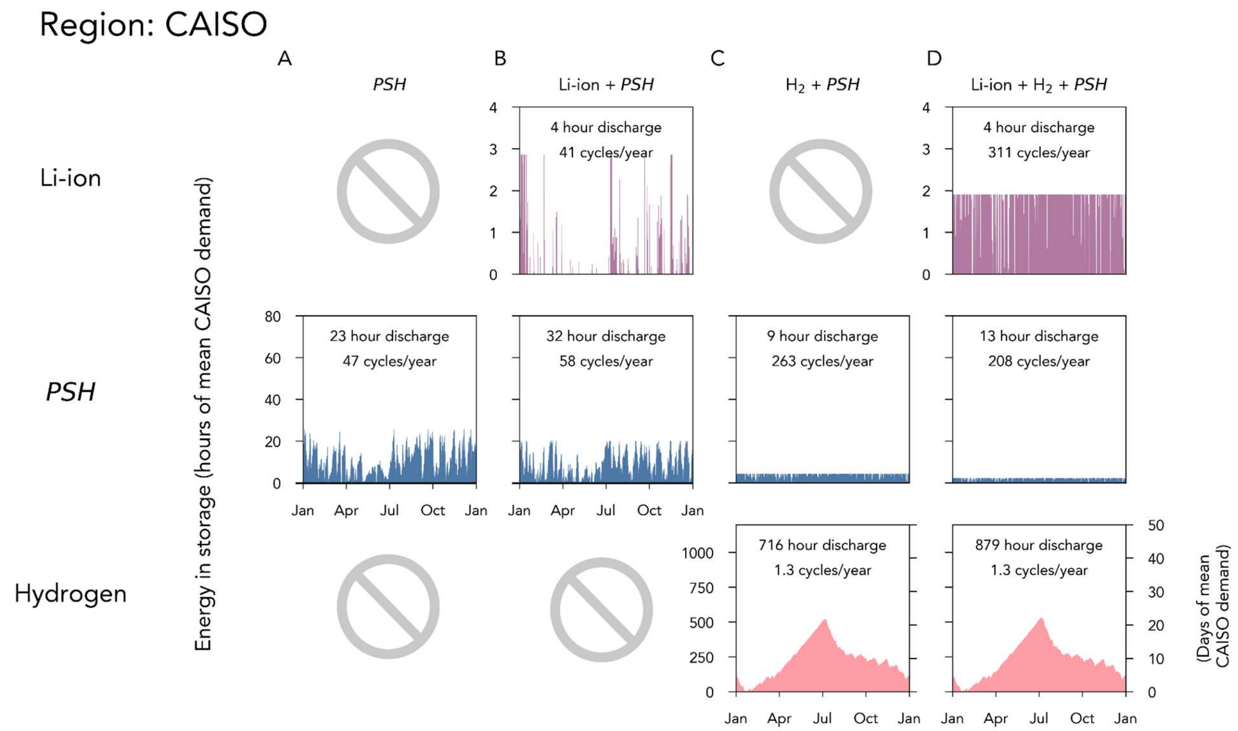

Figure S12. Role of pumped-storage hydropower (PSH) energy storage in CAISO systems with different combinations of short-, mid-, and long-duration storage.

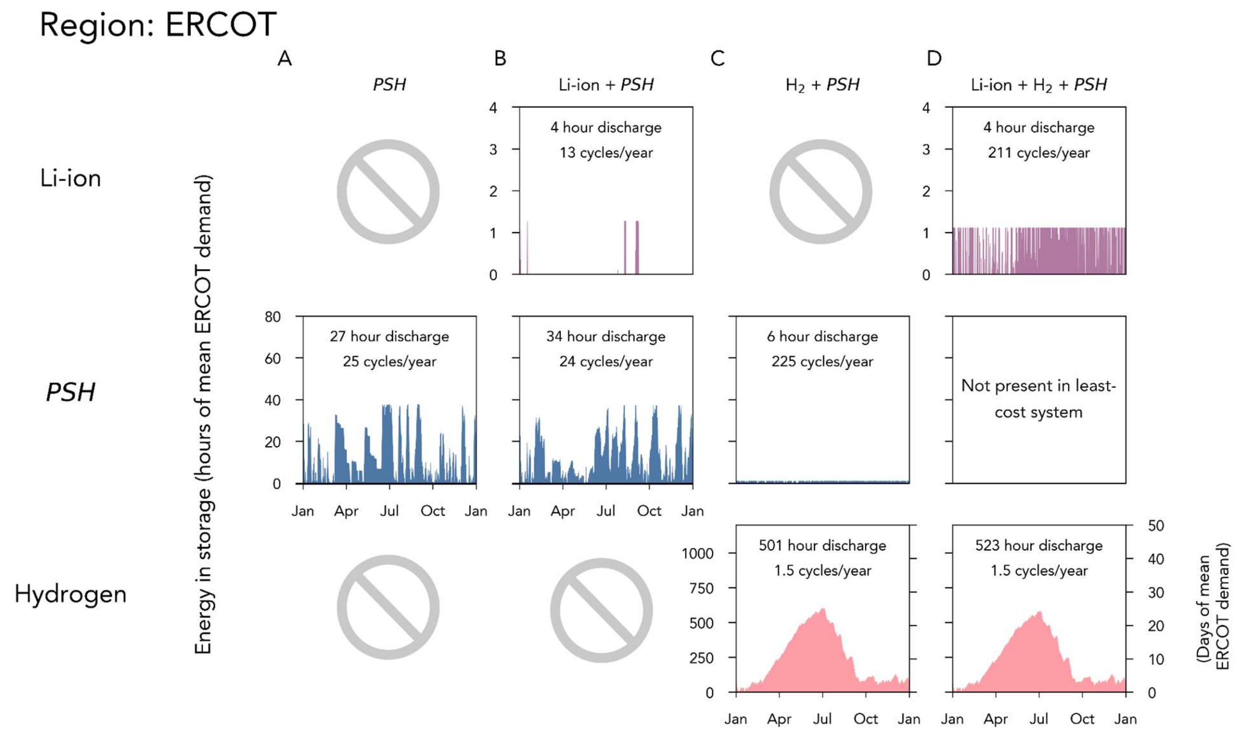

Figure S13. Role of pumped-storage hydropower (PSH) energy storage in ERCOT systems with different combinations of short-, mid-, and long-duration storage.

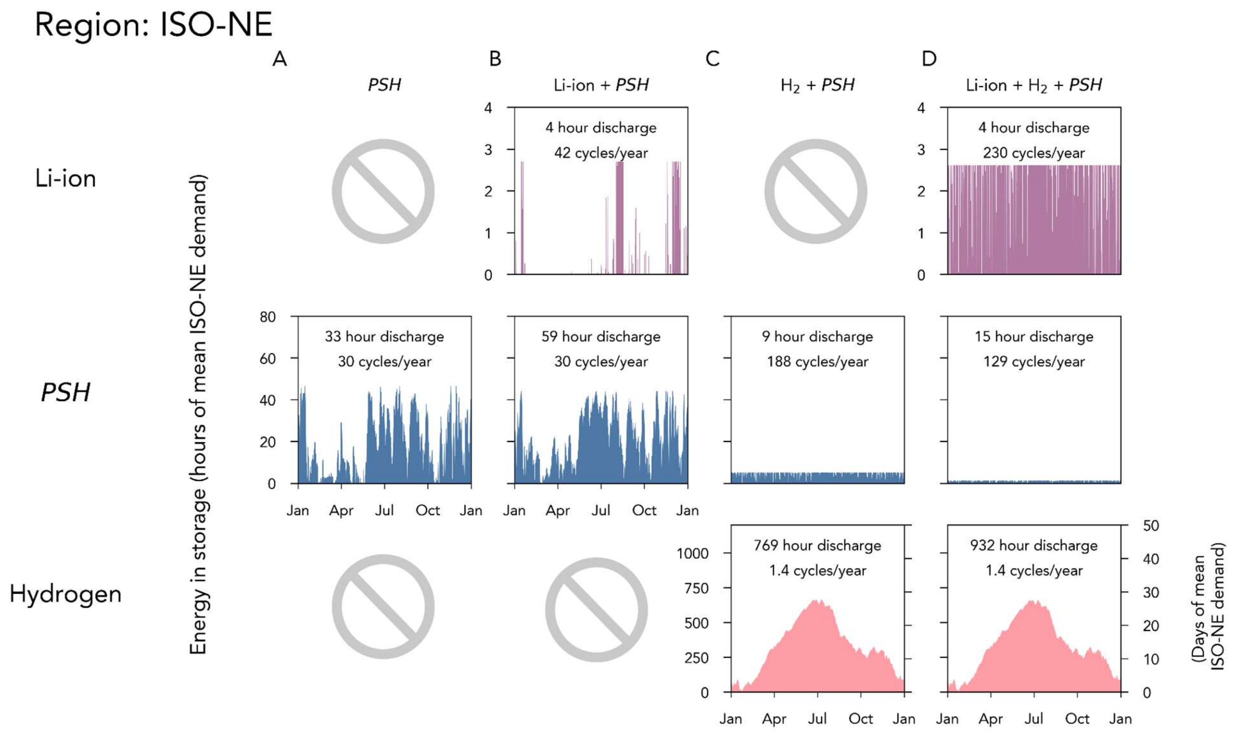

Figure S14. Role of pumped-storage hydropower (PSH) energy storage in ISO-NE systems with different combinations of short-, mid-, and long-duration storage.

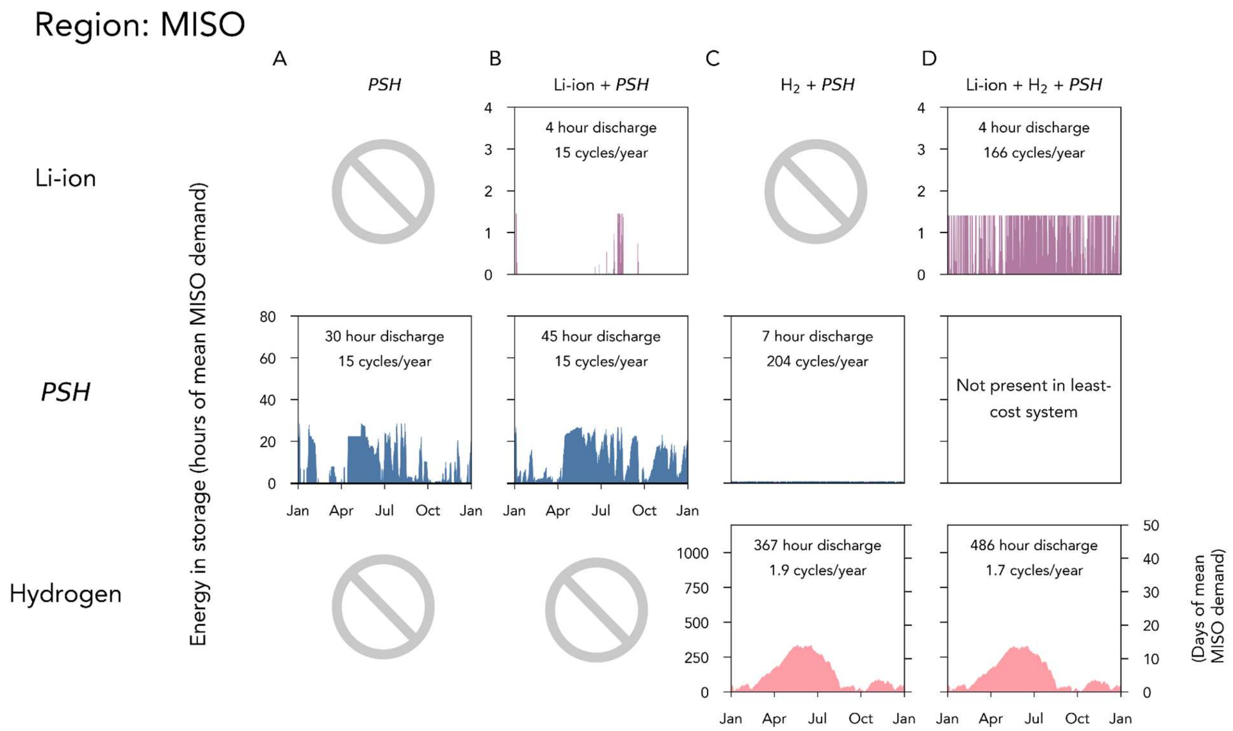

Figure S15. Role of pumped-storage hydropower (PSH) energy storage in MISO systems with different combinations of short-, mid-, and long-duration storage.

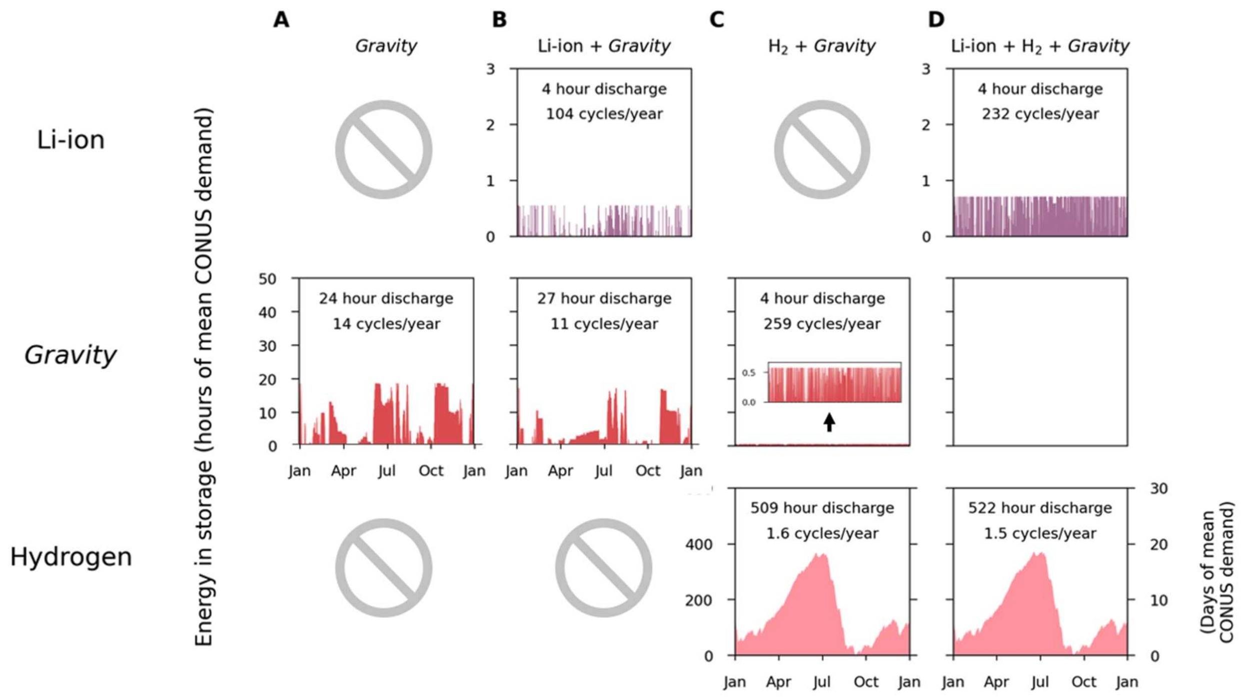

Figure S16. Role of gravity energy storage in CONUS systems with different combinations of short-, mid-, and long-duration storage.

The role (optimized discharge time) of mid-duration storage technologies (here represented by gravity energy storage) depended on the availability of short- and long-duration storage. Energy in storage over one year when:

(A) Gravity energy storage was the only storage technology.
(B) Gravity energy storage competed with short-duration storage (Li-ion batteries).
(C) Gravity energy storage competed with long-duration storage (electrolytic hydrogen).
(D) Gravity energy storage was not present in the least-cost system when both short- and long-duration storage were available.

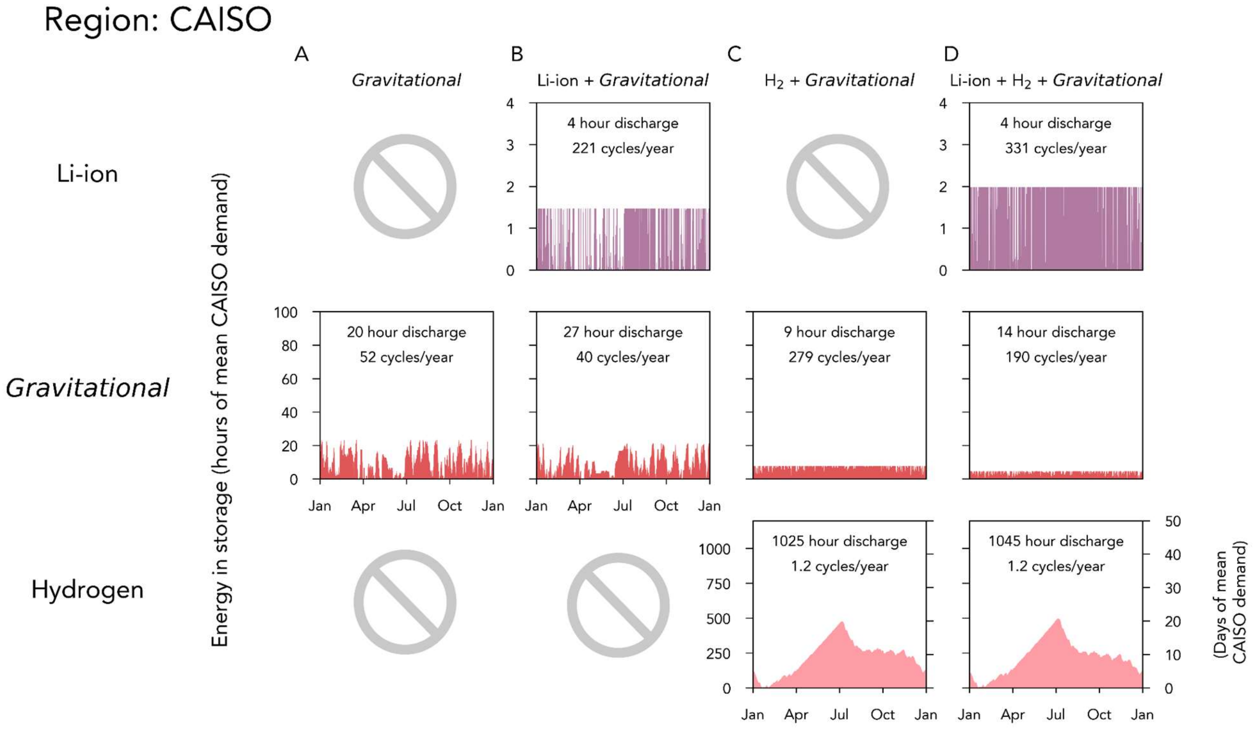

Figure S17. Role of gravity energy storage in CAISO systems with different combinations of short-, mid-, and long-duration storage.

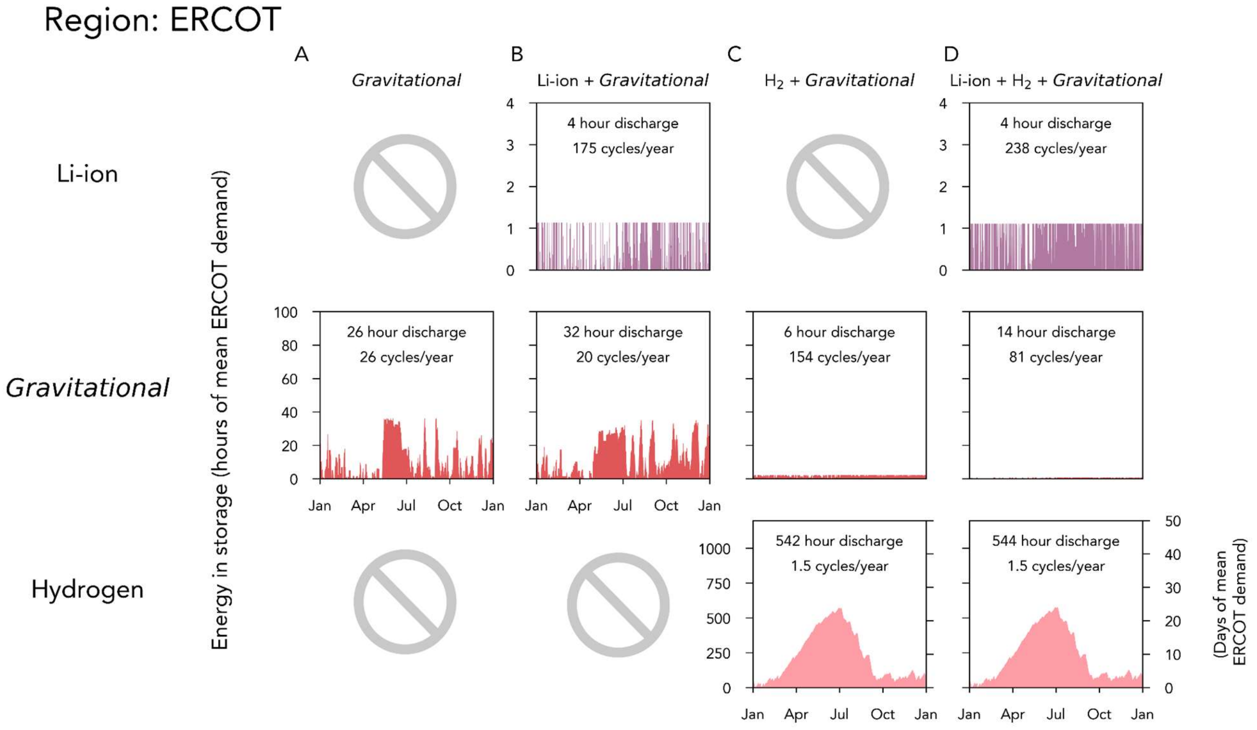

Figure S18. Role of gravity energy storage in ERCOT systems with different combinations of short-, mid-, and long-duration storage.

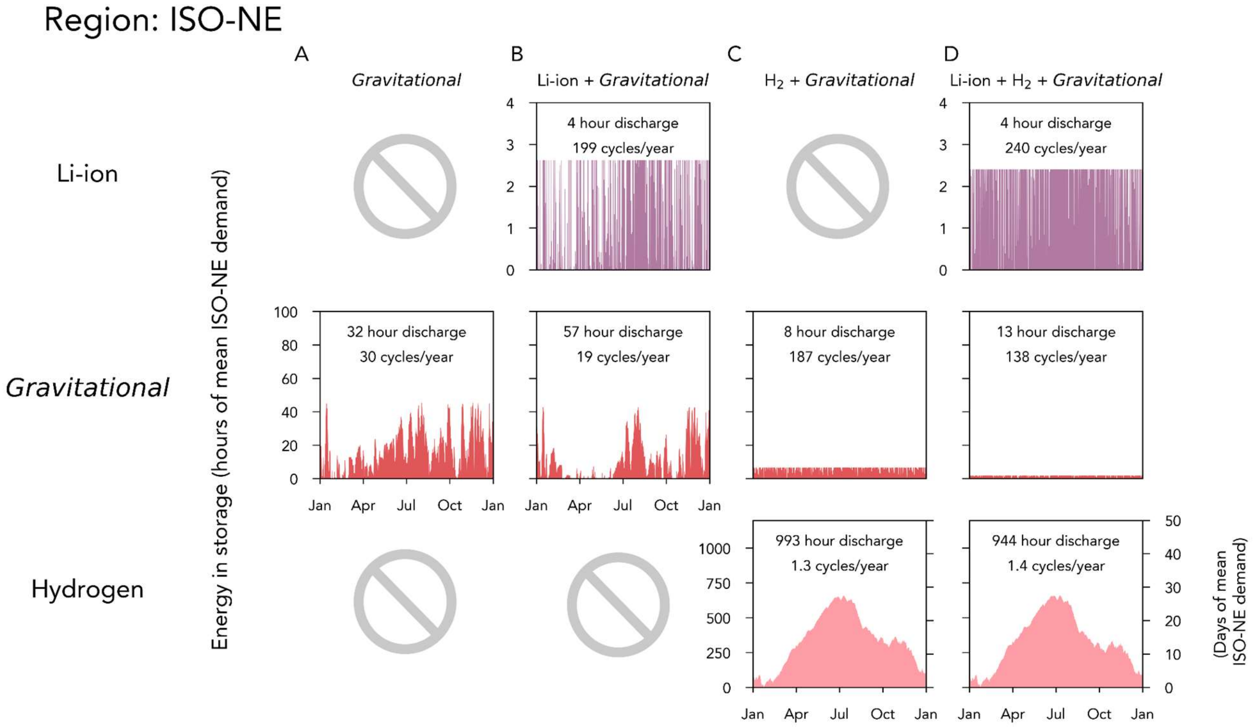

Figure S19. Role of gravity energy storage in ISO-NE systems with different combinations of short-, mid-, and long-duration storage.

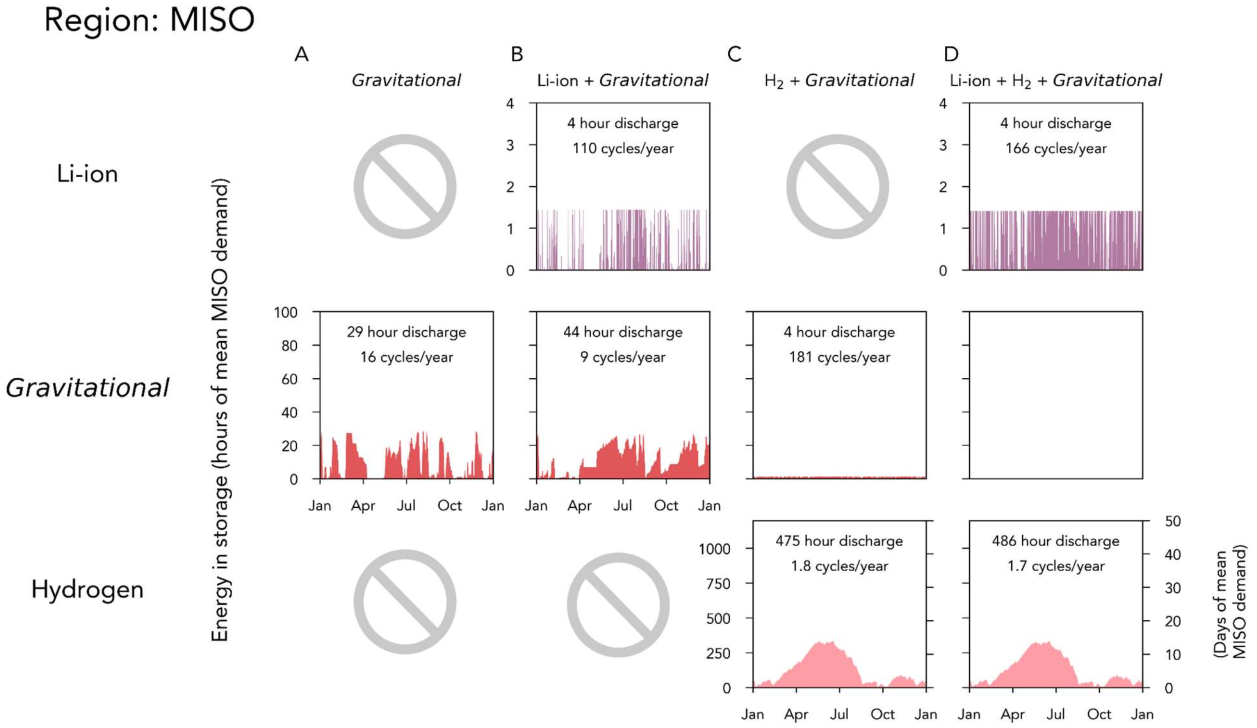

Figure S20. Role of gravity energy storage in MISO systems with different combinations of short-, mid-, and long-duration storage.

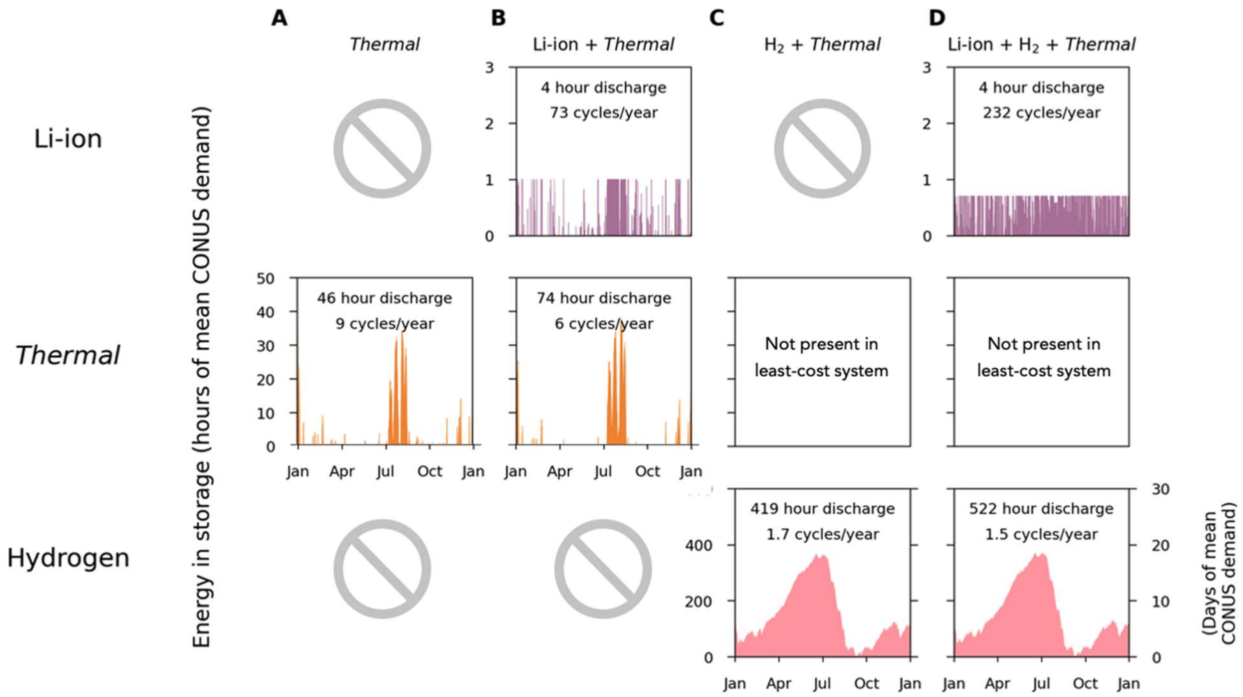

Figure S21. Role of thermal energy storage in CONUS systems with different combinations of short-, mid-, and long-duration storage.

The role (optimized discharge time) of mid-duration storage technologies (here represented by thermal energy storage) depended on the availability of short- and long-duration storage. Energy in storage over one year when:

(A) Thermal energy storage was the only storage technology.
(B) Thermal energy storage competed with short-duration storage (Li-ion batteries).
(C) Thermal energy storage was not present in the least-cost system when long-duration storage (hydrogen) was available.
(D) Thermal energy storage was not present in the least-cost system when both short- and long-duration storage were available.

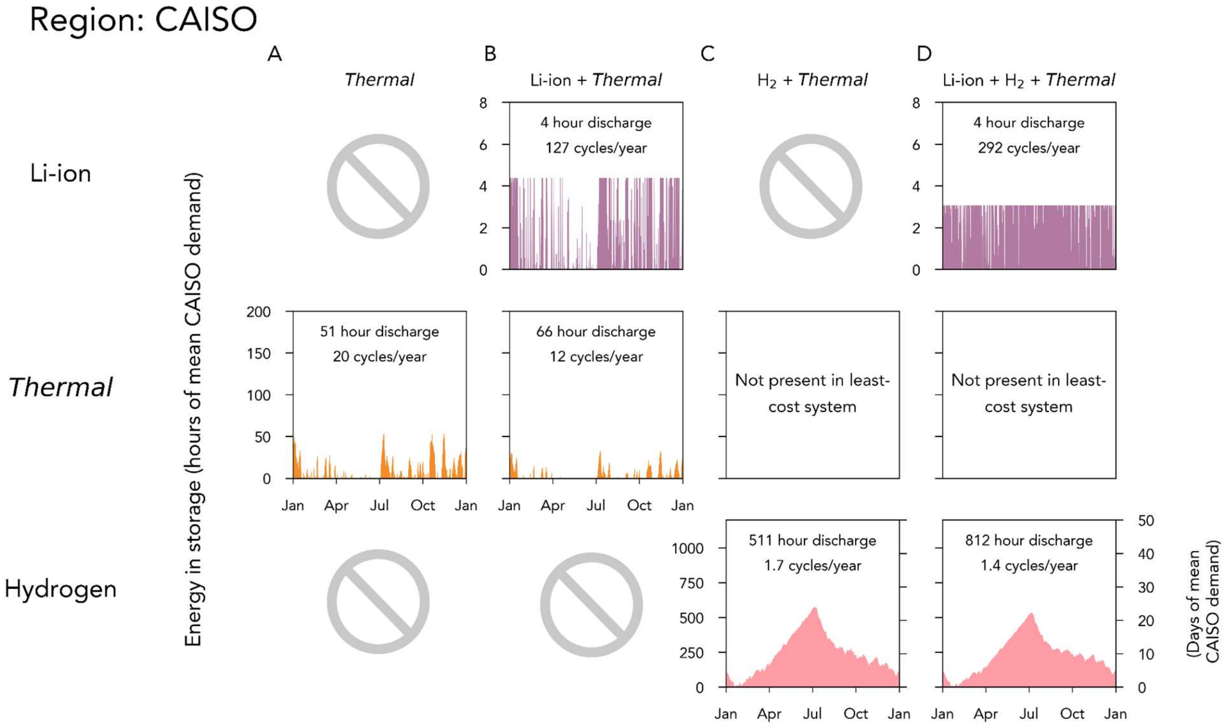

Figure S22. Role of thermal energy storage in CAISO systems with different combinations of short-, mid-, and long-duration storage.

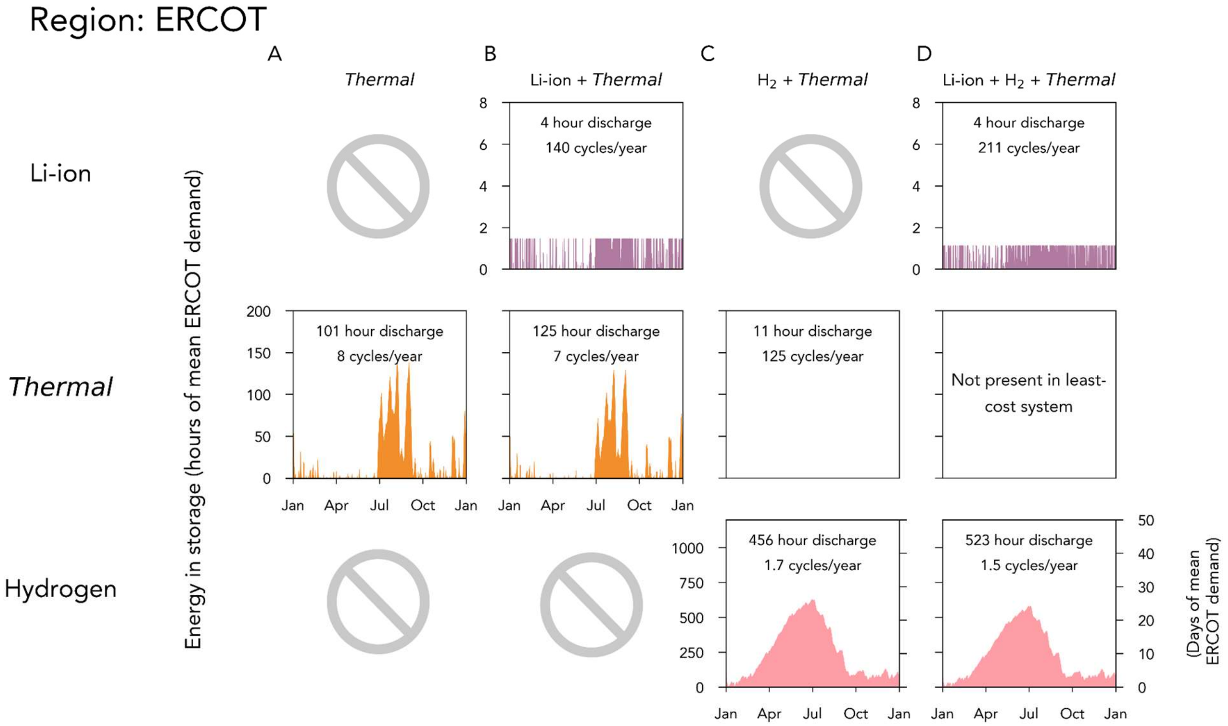

Figure S23. Role of thermal energy storage in ERCOT systems with different combinations of short-, mid-, and long-duration storage.

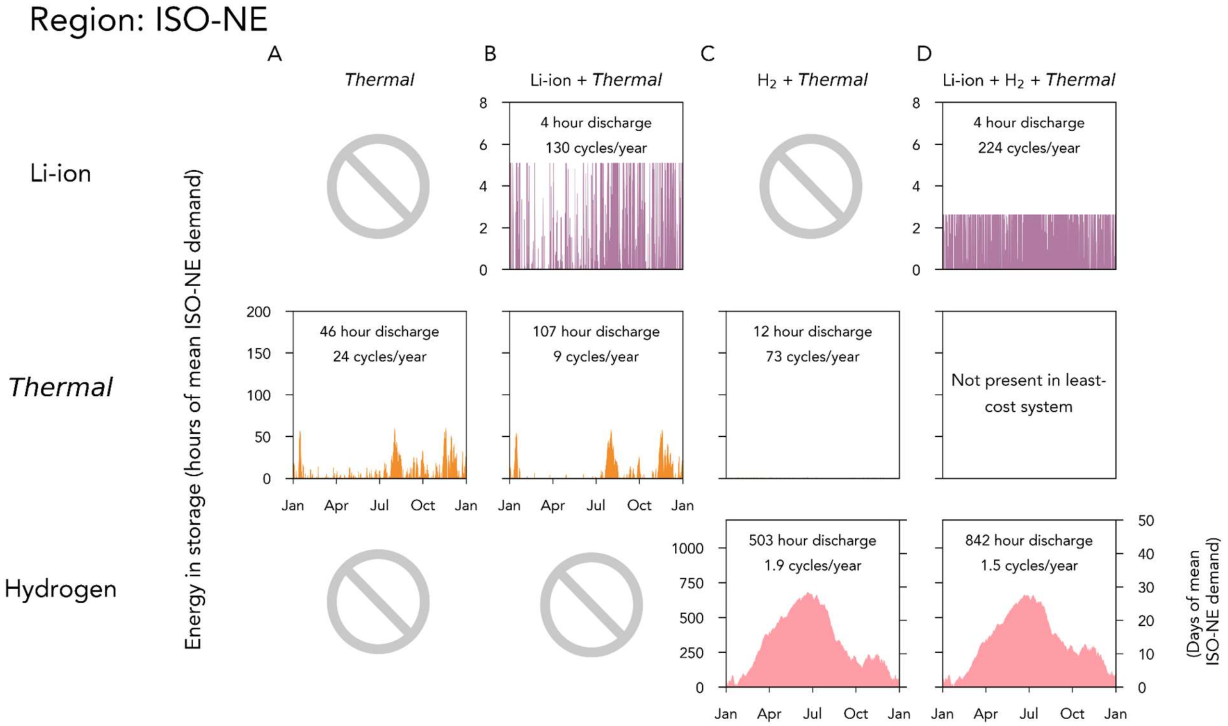

Figure S24. Role of thermal energy storage in ISO-NE systems with different combinations of short-, mid-, and long-duration storage.

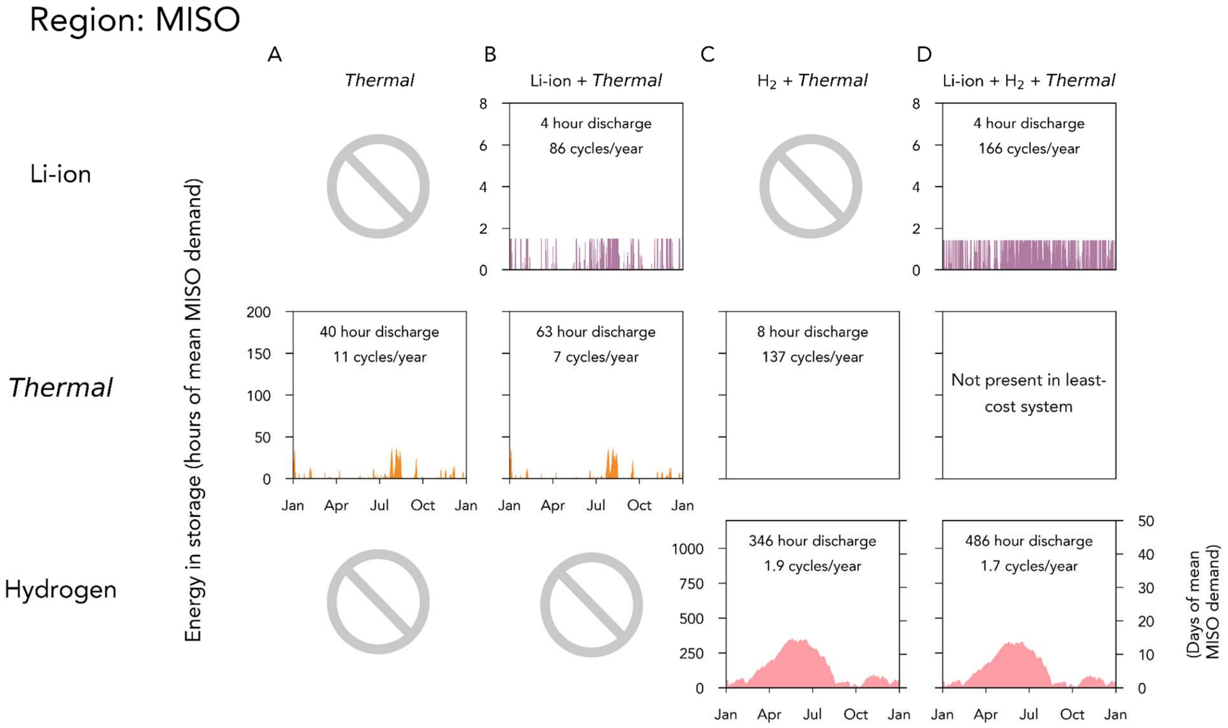

Figure S25. Role of thermal energy storage in MISO systems with different combinations of short-, mid-, and long-duration storage.

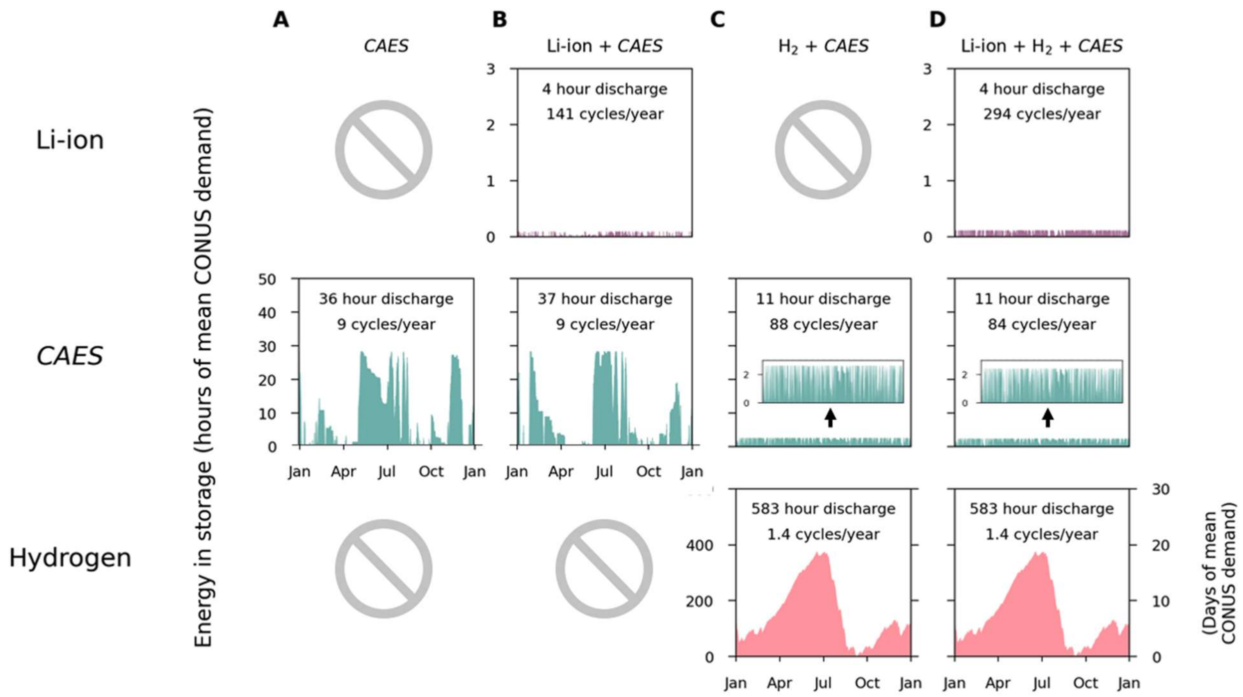

Figure S26. Role of compressed-air energy storage (CAES) in CONUS systems with different combinations of short-, mid-, and long-duration storage.

The role (optimized discharge time) of mid-duration storage technologies (here represented by compressed-air energy storage, CAES) depended on the availability of short- and long-duration storage. Energy in storage over one year when:

(A) CAES was the only storage technology.
(B) CAES competed with short-duration storage (Li-ion batteries).
(C) CAES competed with long-duration storage (electrolytic hydrogen).
(D) CAES competed with both short- and long-duration storage (electrolytic hydrogen).

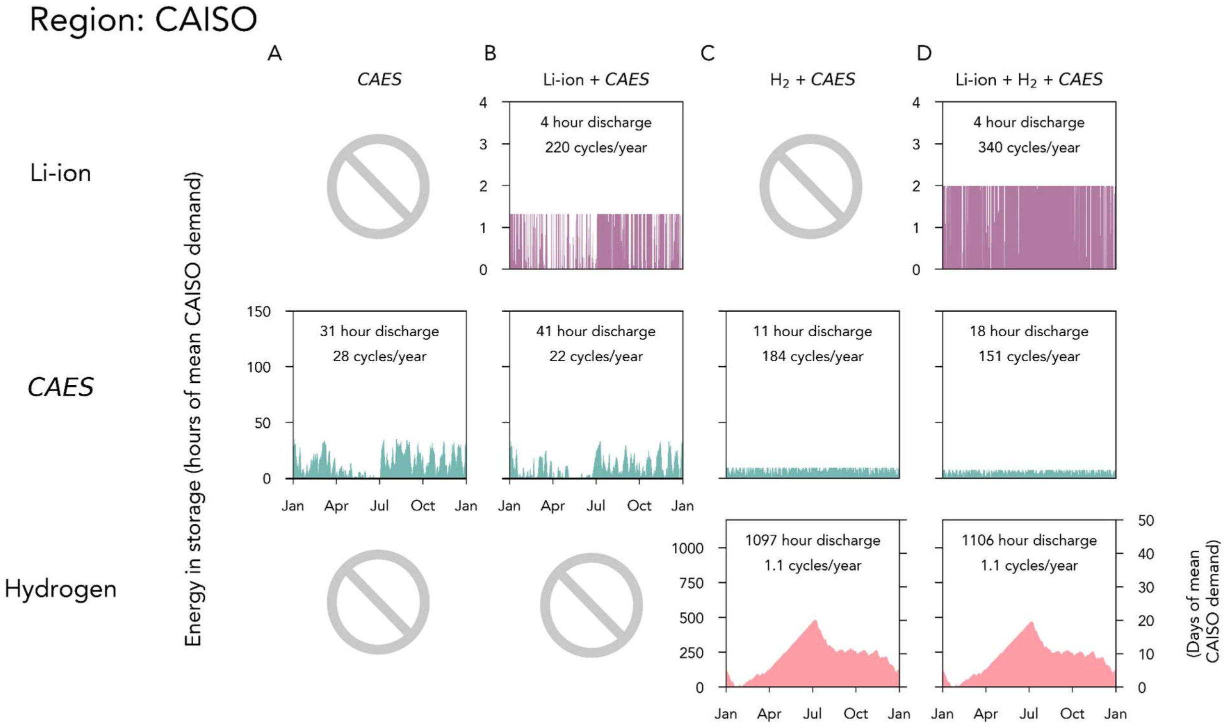

Figure S27. Role of compressed-air energy storage (CAES) in CAISO systems with different combinations of short-, mid-, and long-duration storage.

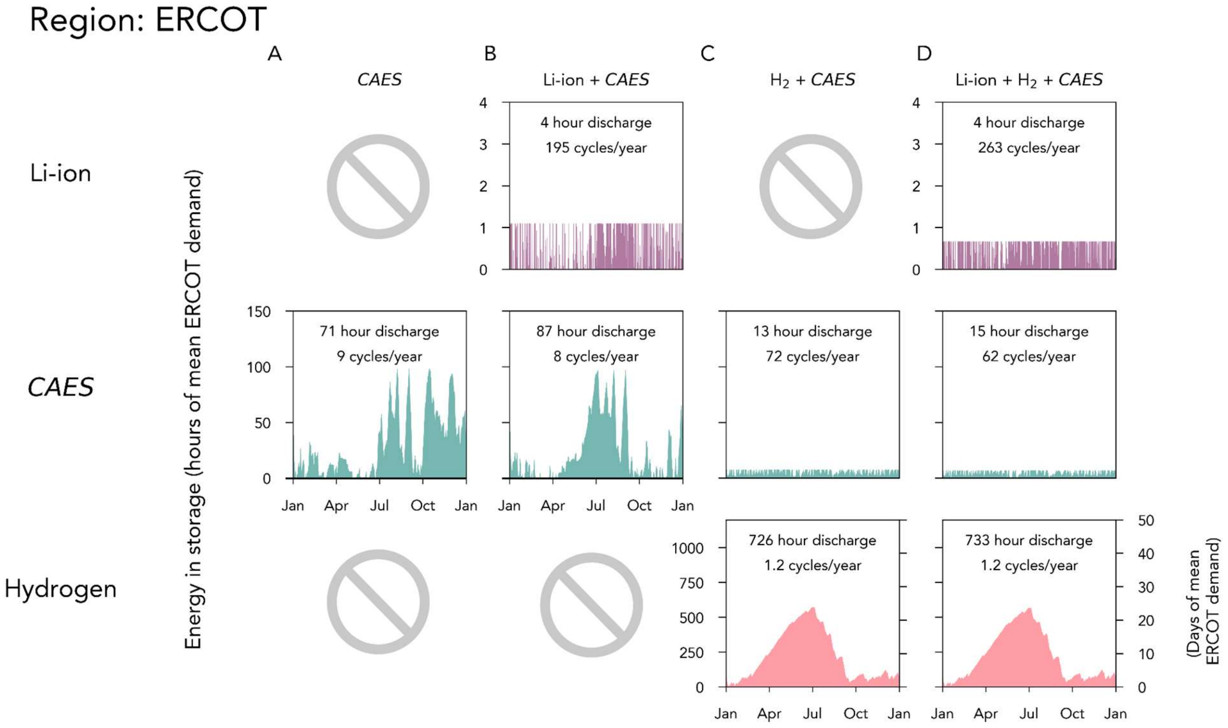

Figure S28. Role of compressed-air energy storage (CAES) in ERCOT systems with different combinations of short-, mid-, and long-duration storage.

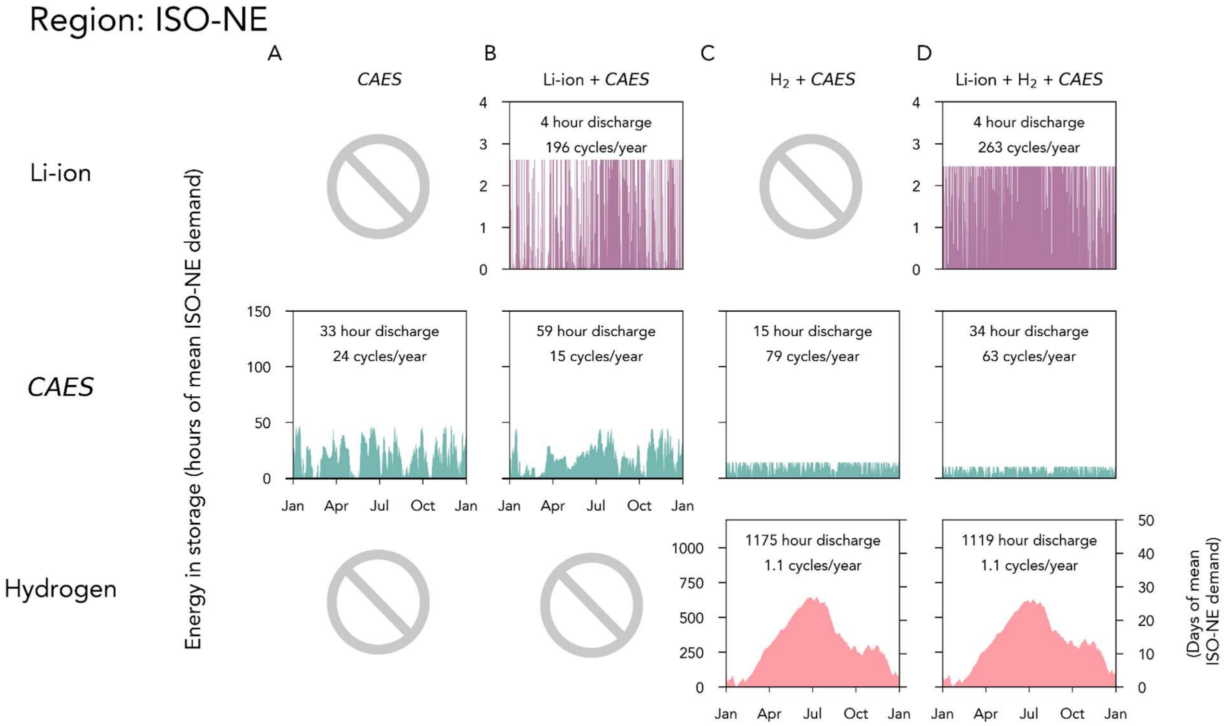

Figure S29. Role of compressed-air energy storage (CAES) in ISO-NE systems with different combinations of short-, mid-, and long-duration storage.

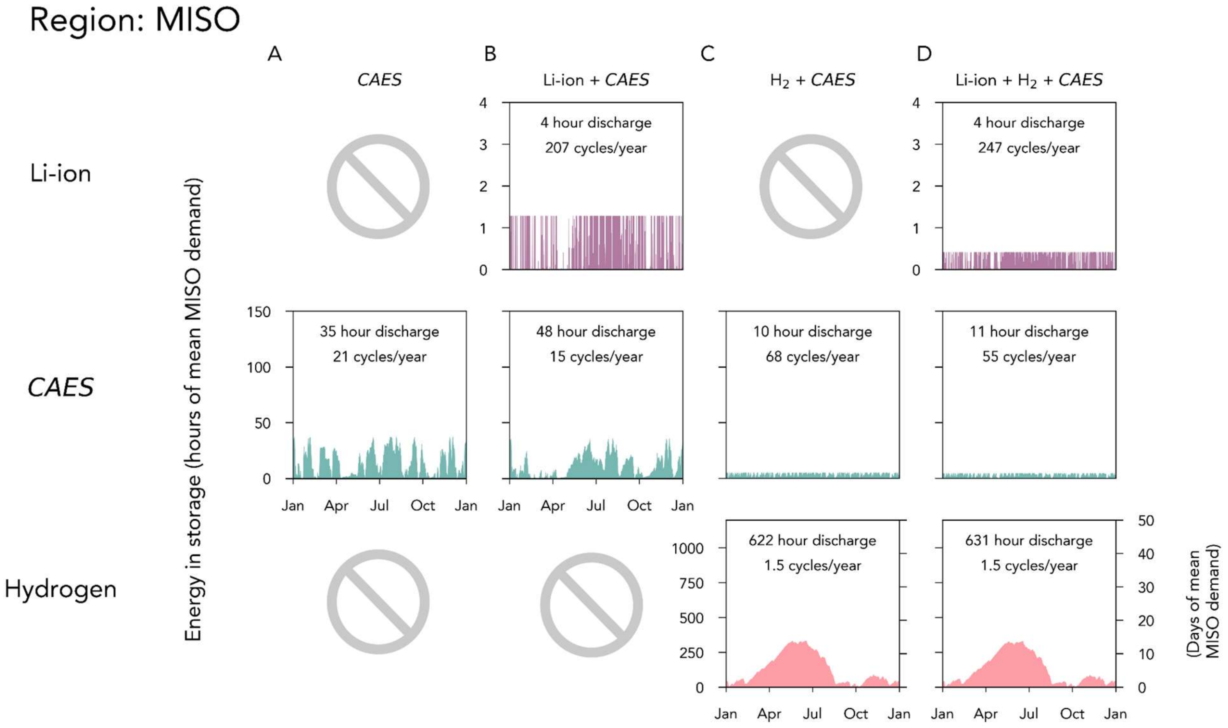

Figure S30. Role of compressed-air energy storage (CAES) in MISO systems with different combinations of short-, mid-, and long-duration storage.

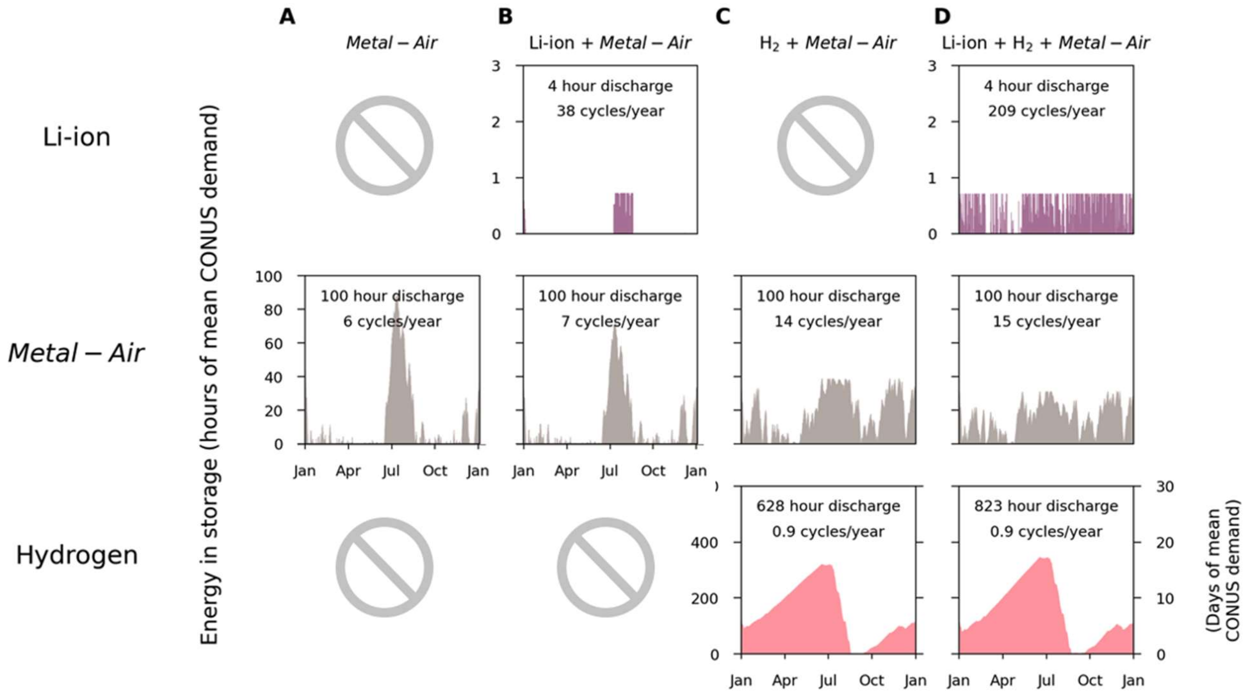

Figure S31. Role of metal-air battery energy storage in CONUS systems with different combinations of short-, mid-, and long-duration storage.

The role (optimized discharge time) of mid-duration storage technologies (here represented by metal-air batteries) depended on the availability of short- and long-duration storage. Energy in storage over one year when:

(A) Metal-air batteries were the only storage technology.
(B) Metal-air batteries competed with short-duration storage (Li-ion batteries).
(C) Metal-air batteries competed with long-duration storage (electrolytic hydrogen)
(D) Metal-air batteries competed with both short- and long-duration storage (electrolytic hydrogen).

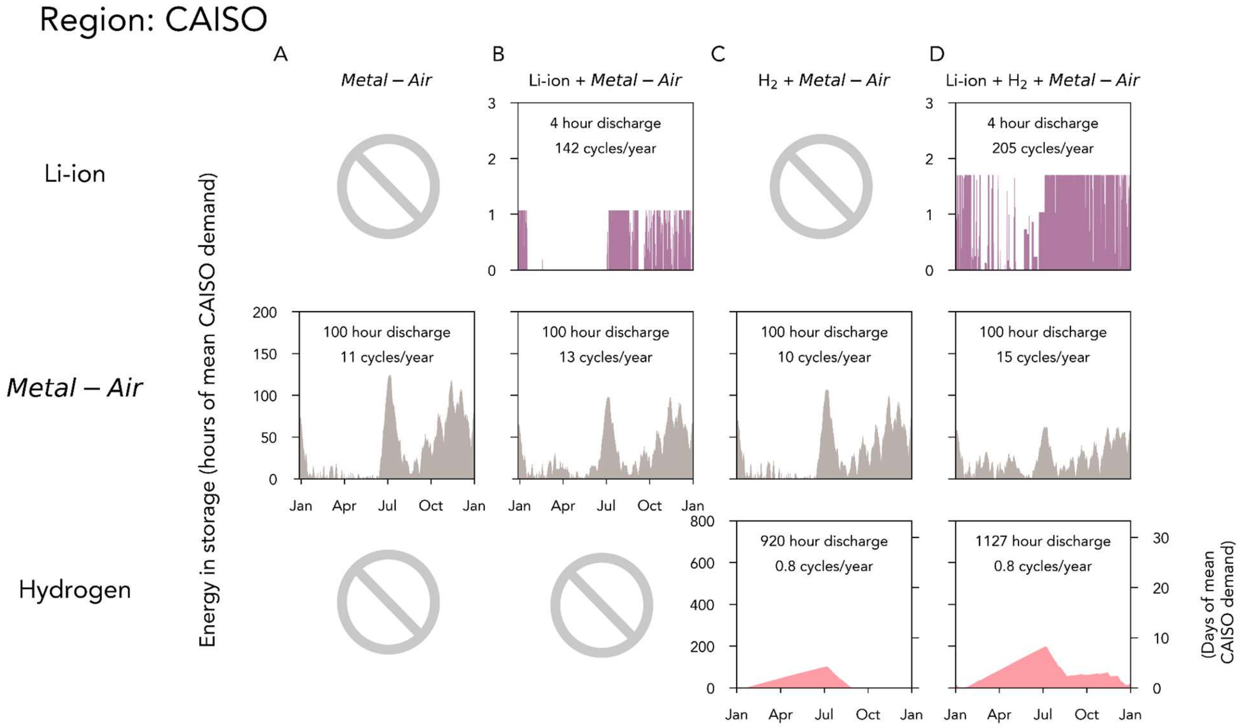

Figure S32. Role of metal-air battery energy storage in CAISO systems with different combinations of short-, mid-, and long-duration storage.

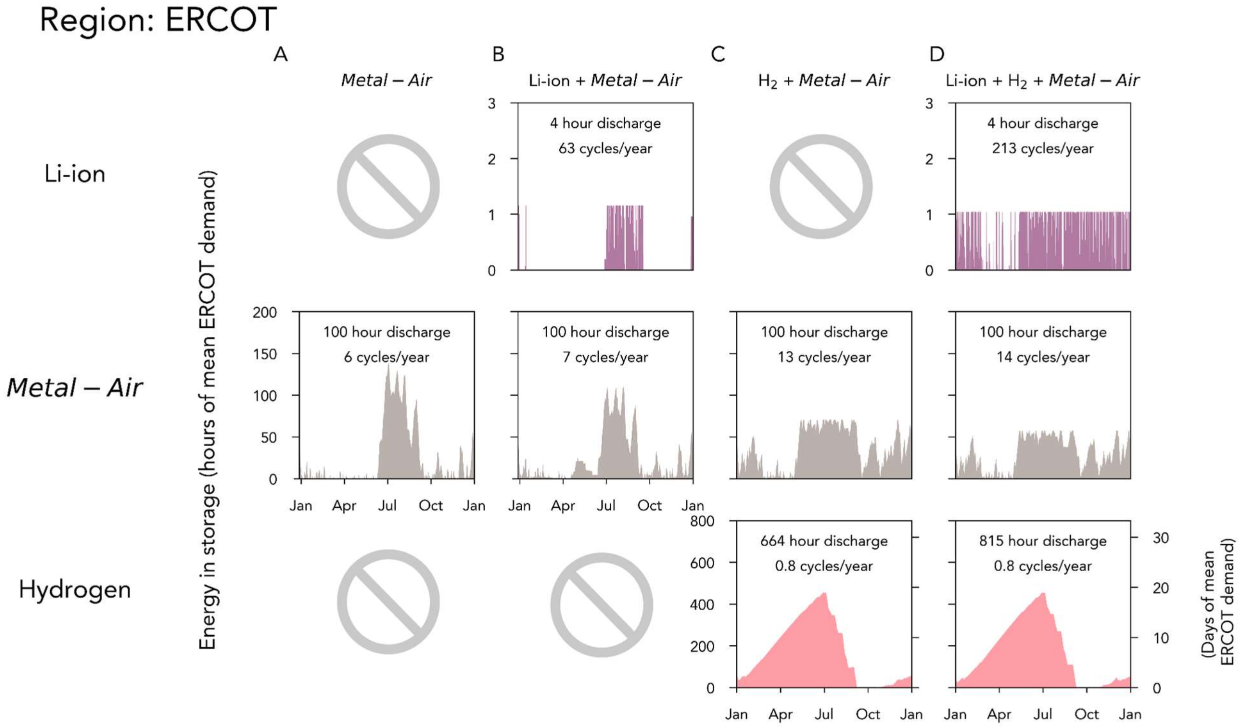

Figure S33. Role of metal-air battery energy storage in ERCOT systems with different combinations of short-, mid-, and long-duration storage.

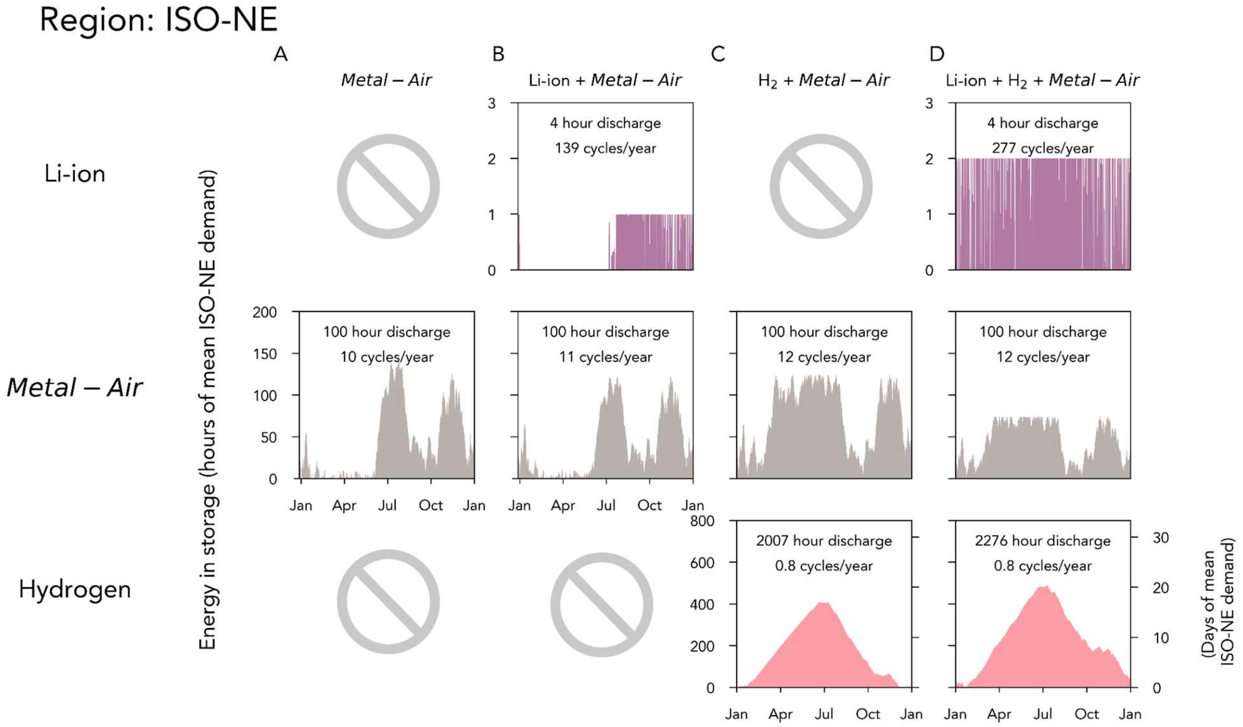

Figure S34. Role of metal-air battery energy storage in ISO-NE systems with different combinations of short-, mid-, and long-duration storage.

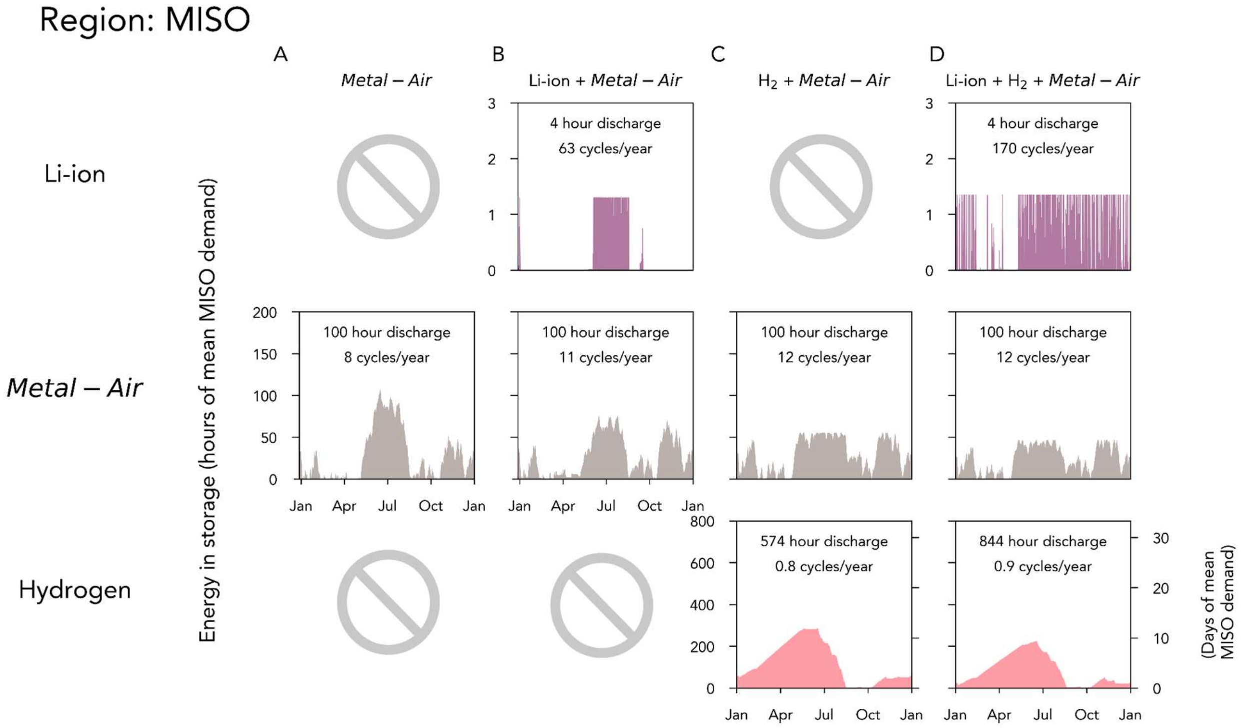

Figure S35. Role of metal-air battery energy storage in MISO systems with different combinations of short-, mid-, and long-duration storage.

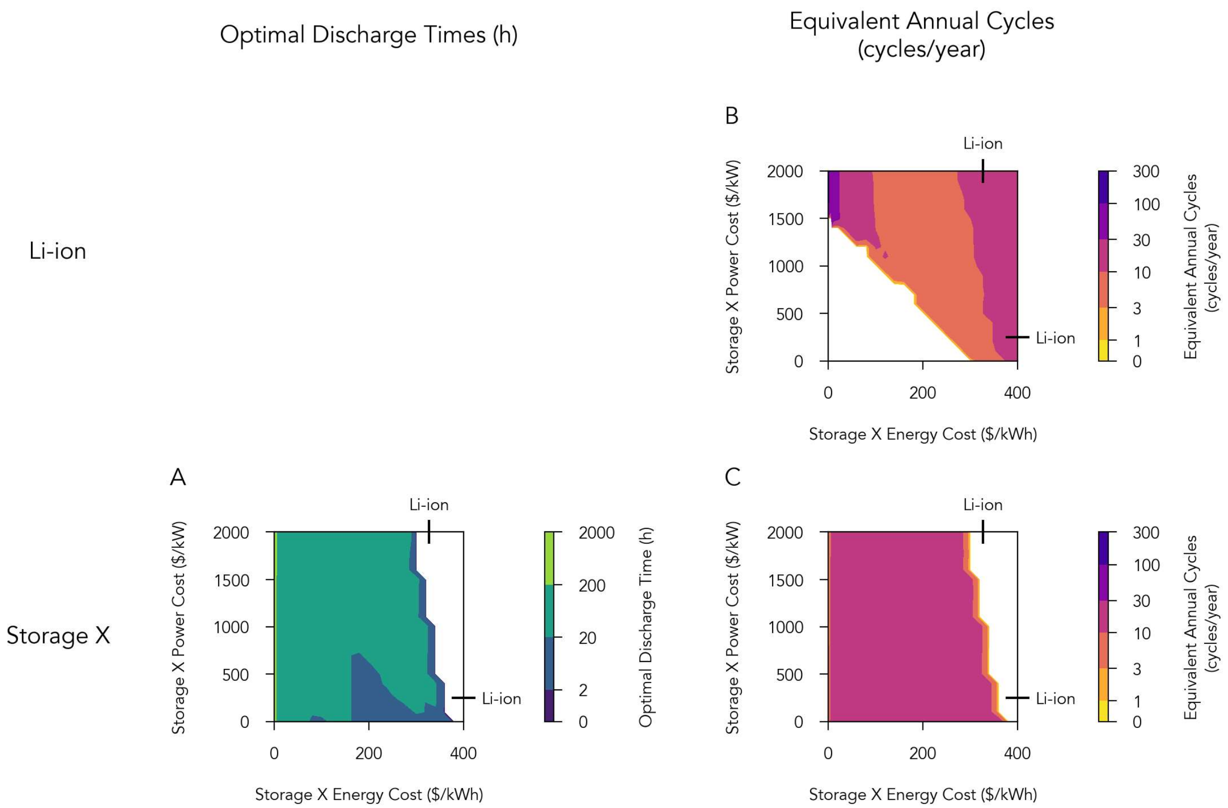

Figure S36. Roles of storage technologies in least-cost CONUS systems with up to two storage options available: short-duration storage (Li-ion) and a hypothetical *Storage X* technology with energy- and power-capacity costs parameterized across wide ranges.

Optimal discharge times in hours of (A) *Storage* X and equivalent annual cycles in cycles per year of (B) Li-ion battery storage and (C) *Storage X*. The optimal discharge time of Li-ion batteries is not shown because they are constrained to a 4-hour duration in the model.

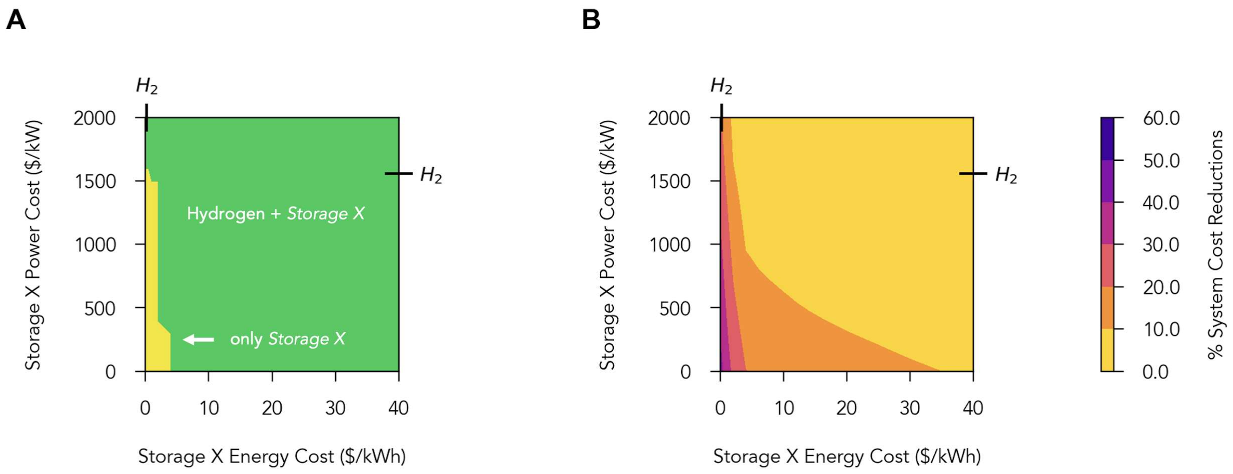

Figure S37. Figure 6 with the x-axes zoomed in to energy-capacity costs from 0 to 40 $/kWh. Storage technologies present in least-cost CONUS systems and system cost reductions in systems with up to two storage options available: long-duration storage (hydrogen) and a hypothetical *Storage X* technology with energy- and power-capacity costs parameterized across wide ranges.

(A) Types of storage technologies used in least-cost systems where Storage X energy- and power-capacity costs are parameterized across wide ranges. The technologies that were present in each parameter range are written in black and white fonts.

(B) Percent reductions in total system cost as compared to a least-cost system with only hydrogen storage at base case costs.

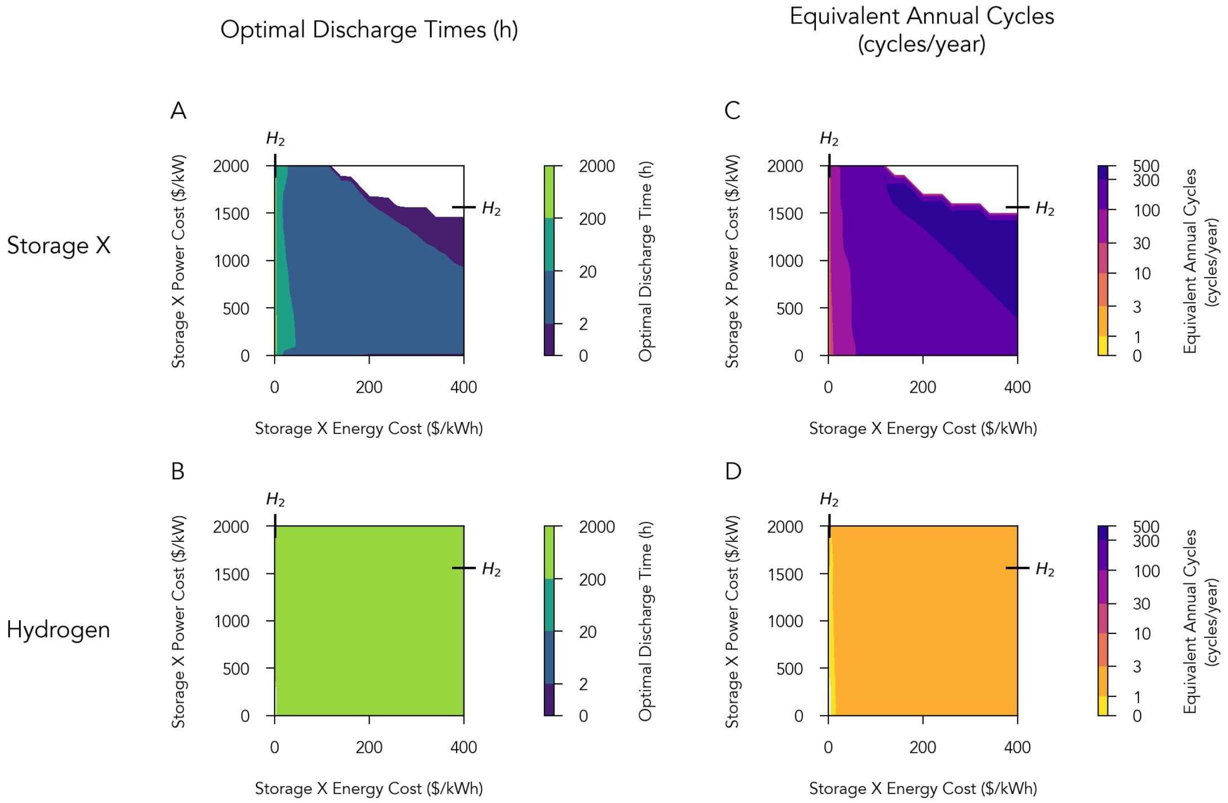

Figure S38. Roles of storage technologies in least-cost CONUS systems with up to two storage options available: long-duration storage (hydrogen) and a hypothetical *Storage X* technology with energy- and power-capacity costs parameterized across wide ranges.

Optimal discharge times in hours of (A) *Storage* X and (B) hydrogen, and equivalent annual cycles in cycles per year of (C) *Storage X* and (D) hydrogen.

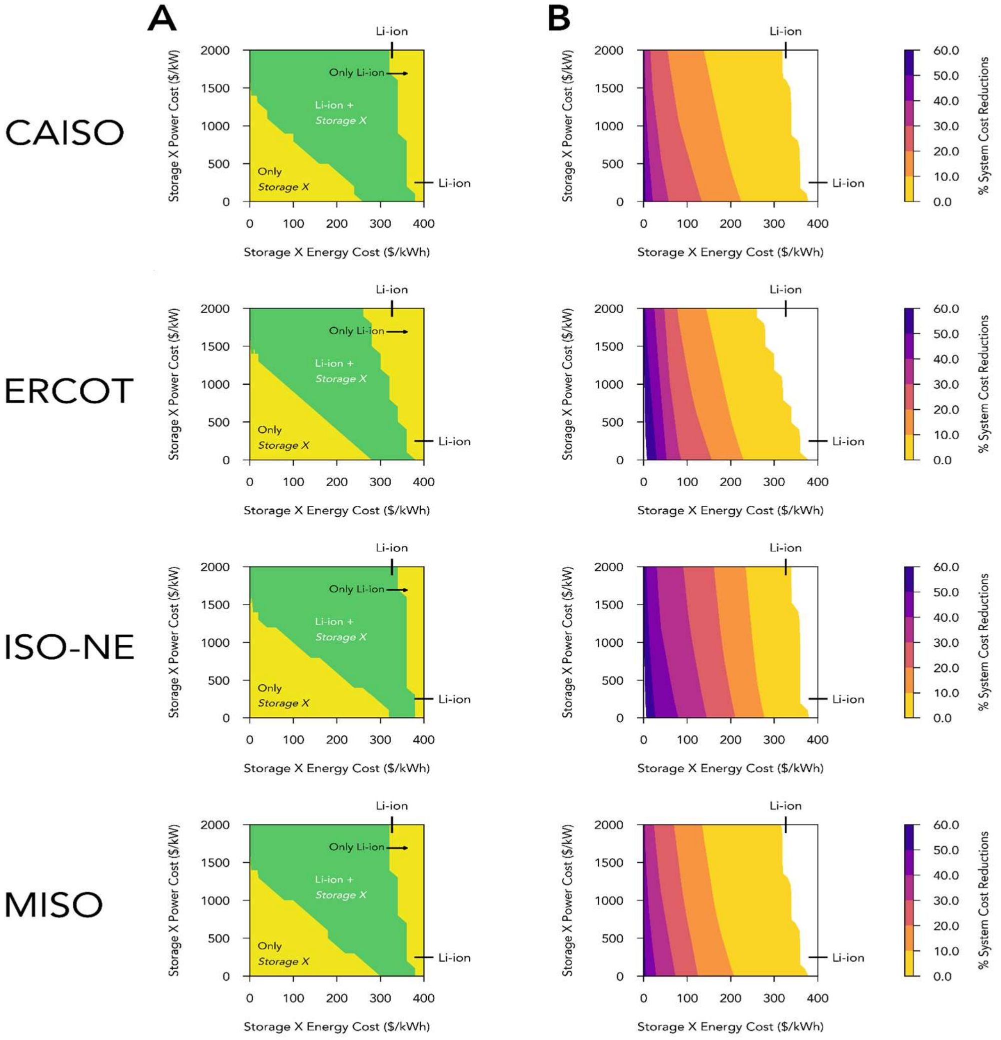

Figure S39. Storage technologies present in least-cost regional ISO systems and system cost reductions in systems with up to two storage options available: short-duration storage (Li-ion) and a hypothetical *Storage X* technology with energy- and power-capacity costs parameterized across wide ranges.

Modeling parameters for Li-ion batteries were kept constant at base-case values, with Li-ion battery energy- and power-capacity costs marked on the top and right sides of the plot and numerical values in Table S4. Note that the energy- and power-capacity ratio (duration) of Li-ion batteries was fixed at 4 h. The round-trip efficiency of *Storage X* was fixed at 86%, to match the round-trip efficiency of Li-ion batteries.

(A) Types of storage technologies used in 100% reliable least-cost systems in which *Storage X* energy- and power-capacity costs were varied across wide ranges. The technologies that were present in each parameter range are written in black and white fonts.

(B) Percent reductions in total system cost as compared to a least-cost system with only Li-ion battery storage at base case costs.

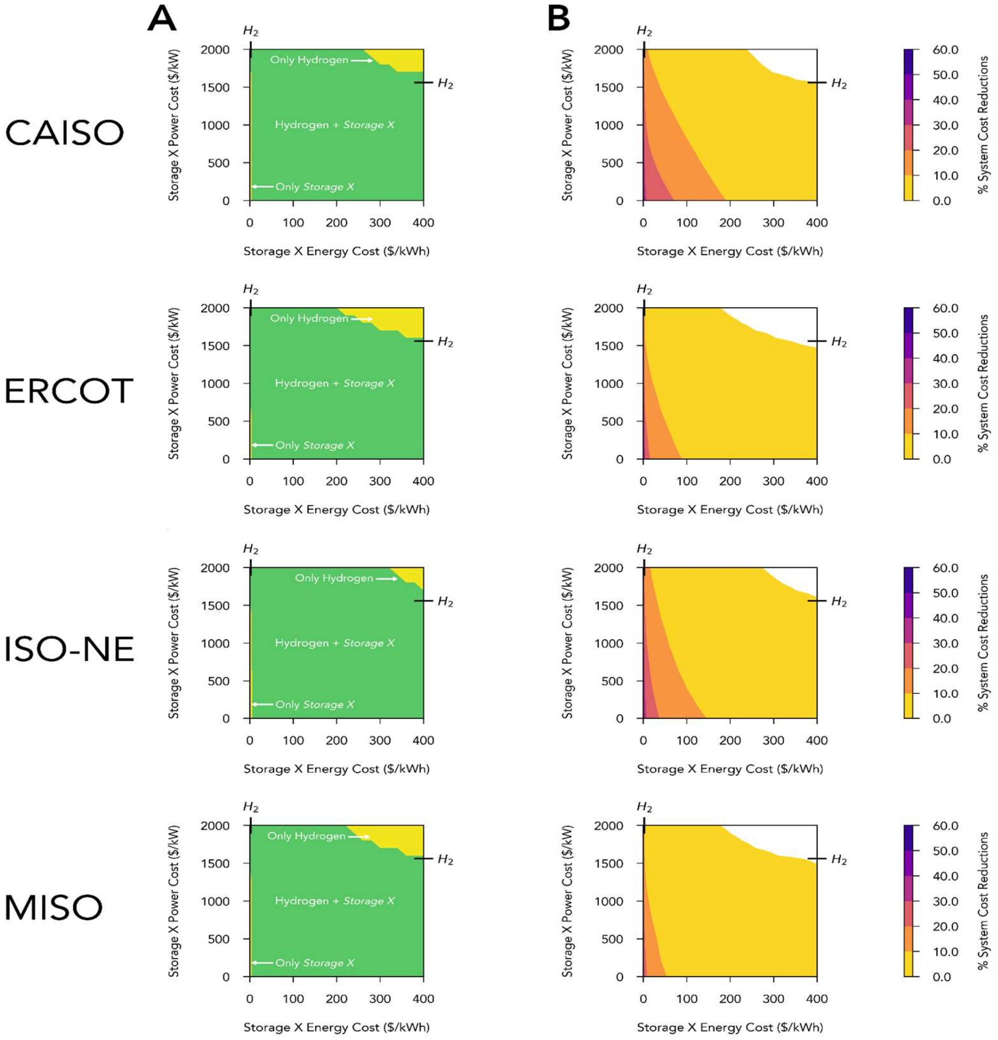

Figure S40. Storage technologies present in least-cost regional ISO systems and system cost reductions in systems with up to two storage options available: long-duration storage (hydrogen) and a hypothetical *Storage X* technology with energy- and power-capacity costs parameterized across wide ranges.

Modeling parameters for hydrogen storage were fixed at base-case values, with hydrogen storage energy- and power-capacity costs marked on the top and right sides of the plot, with exact numerical values presented in Table S4. The round-trip efficiency of *Storage X* was fixed at 86%, to match the round-trip efficiency of Li-ion batteries.

(A) Types of storage technologies used in least-cost 100% reliable systems in which Storage X energy- and power-capacity costs are parameterized across wide ranges. The technologies that were present in each parameter range are written in black and white fonts.

(B) Percent reductions in total system cost as compared to a least-cost system with only hydrogen storage at base case costs.

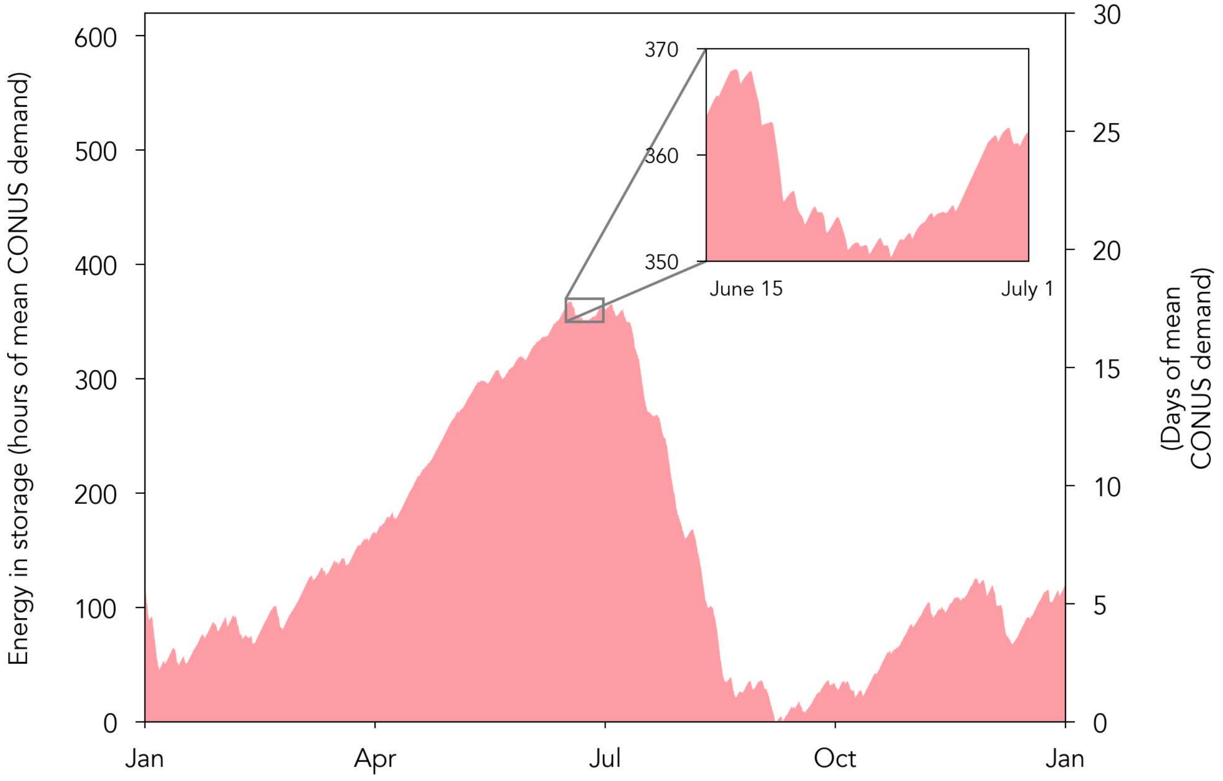

Figure S41. Energy in storage over one year when hydrogen energy storage is the only storage technology available in a least-cost CONUS electricity system that relies on wind and solar generation.

The inset plot shows hydrogen charging and discharging on an hourly/daily basis during the time period from June 15 to July 1 in the model, serving a short-term storage role in addition to long-term storage.

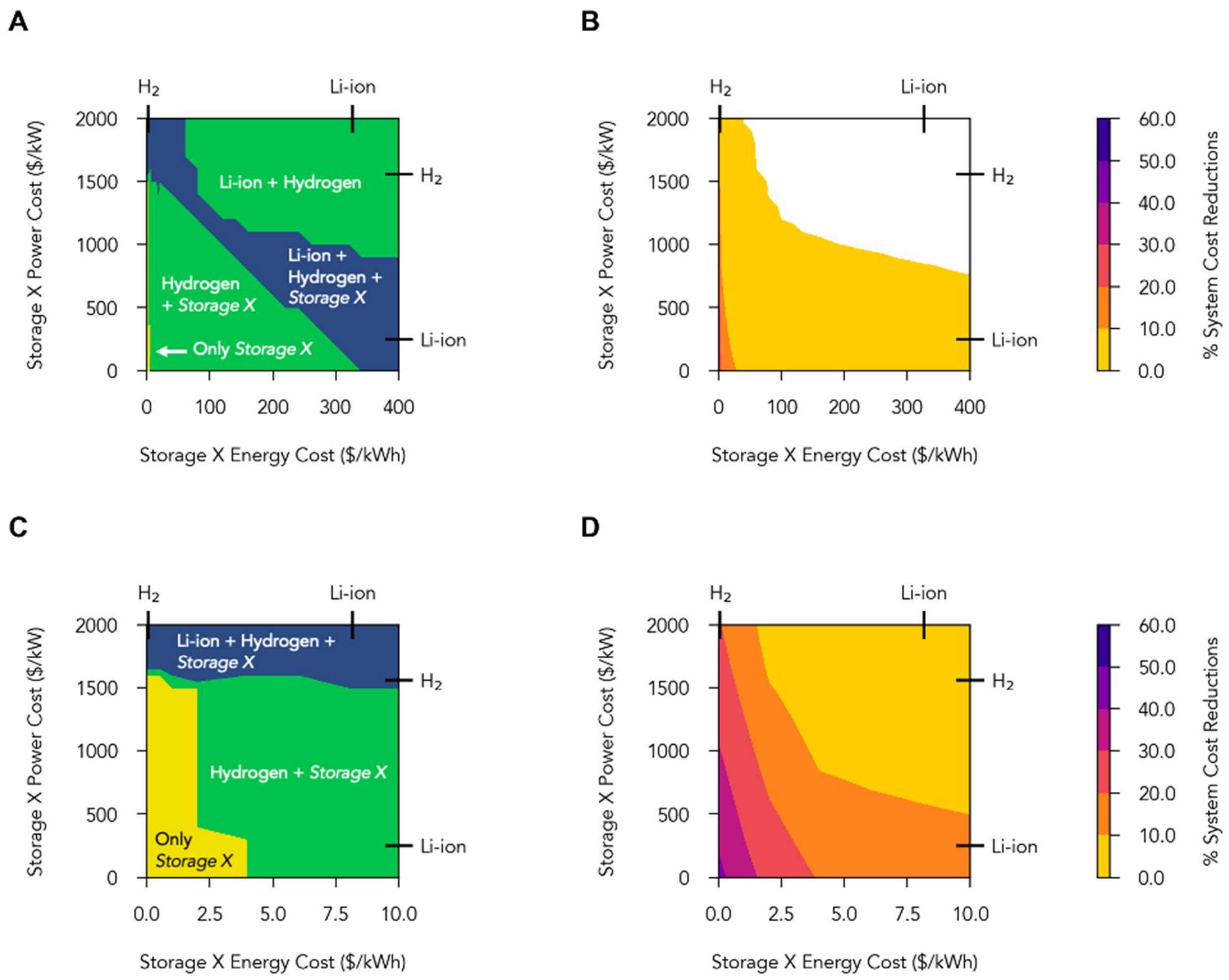

Figure S42. Storage technologies present in least-cost CONUS systems and system cost reductions in systems with up to three storage options available: short-duration storage (Li-ion), long-duration storage (hydrogen), and a hypothetical *Storage X* technology with energy- and power-capacity costs parameterized across wide ranges.

Modeling parameters for Li-ion and hydrogen storage were kept constant at base-case values, with their energy- and power-capacity costs marked on the top and right sides of the plot and numerical values in Table 2. Note that the energy- to power-capacity ratio of Li-ion batteries are kept at a ratio of 4 hours. The round-trip efficiency of *Storage X* is kept at 86%, to match the round-trip efficiency of Li-ion batteries.

(A) Types of storage technologies used in least-cost systems where *Storage X* energy- and power-capacity costs are parameterized across wide ranges. The technologies that were present in each parameter range are written in black and white fonts.

(B) Percent reductions in total system cost as compared to a least-cost system with only Li-ion and hydrogen storage at base case costs.

(C) Panel A, but with the x-axis zoomed in to energy-capacity costs from 0 to 10 $/kWh.

(D) Panel B, but with the x-axis zoomed in to energy-capacity costs from 0 to 10 $/kWh.

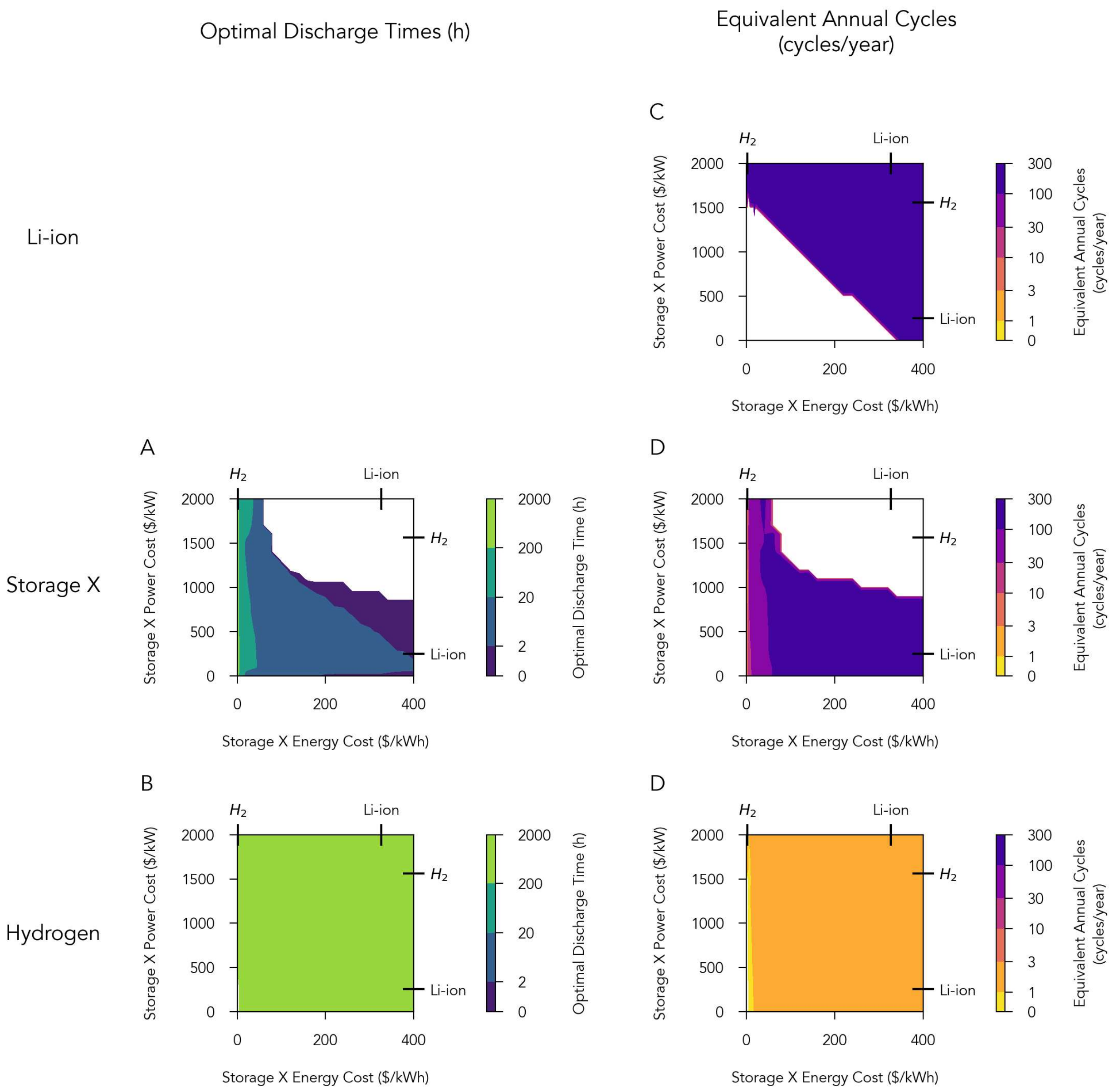

Figure S43. Roles of storage technologies in least-cost CONUS systems with up to three storage options available: short-duration storage (Li-ion), long-duration storage (hydrogen), and a hypothetical *Storage X* technology with energy- and power-capacity costs parameterized across wide ranges.

Optimal discharge times in hours of (A) *Storage* X and (B) hydrogen storage, and equivalent annual cycles in cycles per year of (C) Li-ion battery storage, (D) *Storage X*, and (E) hydrogen storage.

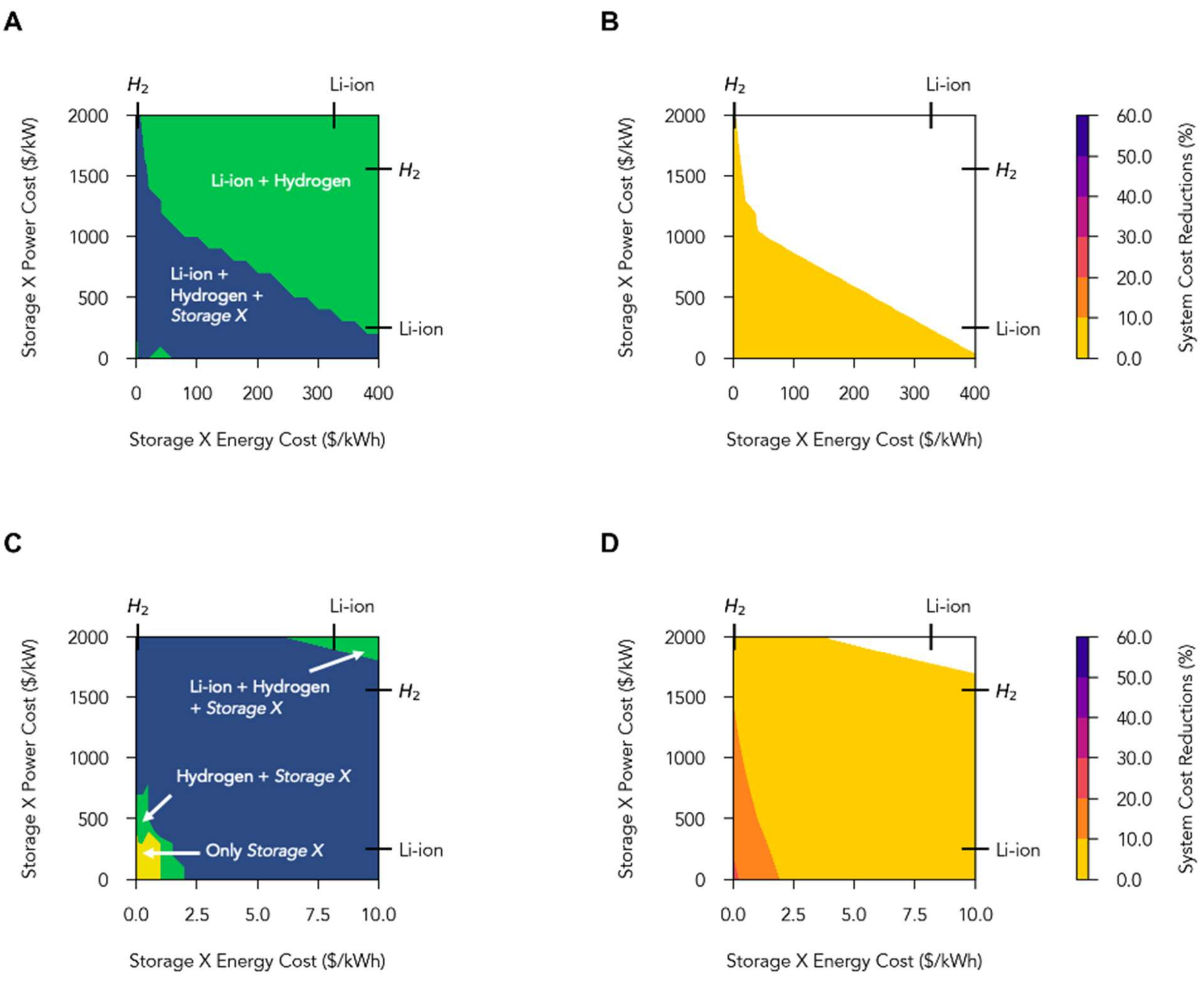

Figure S44. Analogous plot to Figure S42, but with a lower *Storage X* round-trip efficiency. *Storage* technologies present in least-cost CONUS systems and system cost reductions in systems with up to three storage options available: short-duration storage (Li-ion), long-duration storage (hydrogen), and a hypothetical *Storage X* technology with energy- and power-capacity costs parameterized across wide ranges.

Modeling parameters for Li-ion and hydrogen storage were kept constant at base-case values, with their energy- and power-capacity costs marked on the top and right sides of the plot and numerical values in Table S1. Note that the energy- to power-capacity ratio of Li-ion batteries were kept at a ratio of 4 hours. The round-trip efficiency of *Storage X* was kept at 36%, to match the round-trip efficiency of hydrogen storage.

(A) Types of storage technologies used in least-cost systems where *Storage X* energy- and power-capacity costs are parameterized across wide ranges. The technologies that were present in each parameter range are written in white font.
(B) Percent reductions in total system cost as compared to a least-cost system with only Li-ion and hydrogen storage at base case costs.
(C) Panel A with the x-axis zoomed in to energy-capacity costs from 0 to 10 $/kWh.
(D) Panel B with the x-axis zoomed in to energy-capacity costs from 0 to 10 $/kWh.

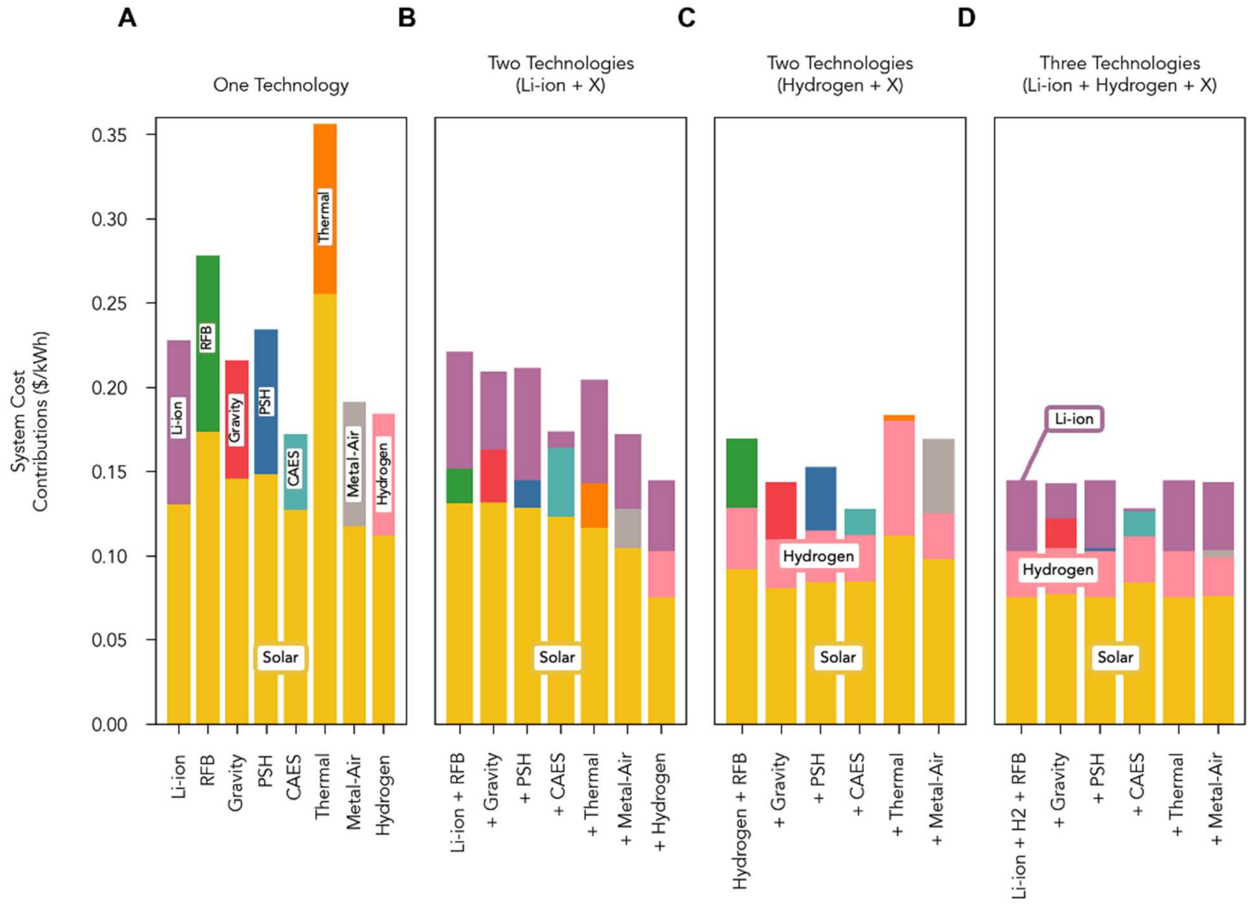

Figure S45. The value of short-duration storage (Li-ion batteries), and other technologies with low power capacity cost, is emphasized in a CONUS system with only solar generation.

Cost contributions of technologies in systems with only solar generation, with one, two, and three storage technologies. System costs when:

(A) Only one storage technology was available: Li-ion batteries, *RFB* (redox-flow batteries), *Gravity* energy storage, *PSH* (Pumped-Storage Hydropower), *CAES* (Compressed Air Energy Storage), *Thermal* energy storage, *Metal-Air* battery storage, or hydrogen energy storage.

(B) Two storage technologies were available: Li-ion batteries, with the second storage technology being a mid-duration storage technology or hydrogen energy storage.

(C) Two storage technologies were available: Hydrogen energy storage, with the second storage technology being a mid-duration storage technology.

(D) Three storage technologies were available: Li-ion batteries and hydrogen energy storage, with the third storage technology being a mid-duration storage technology.

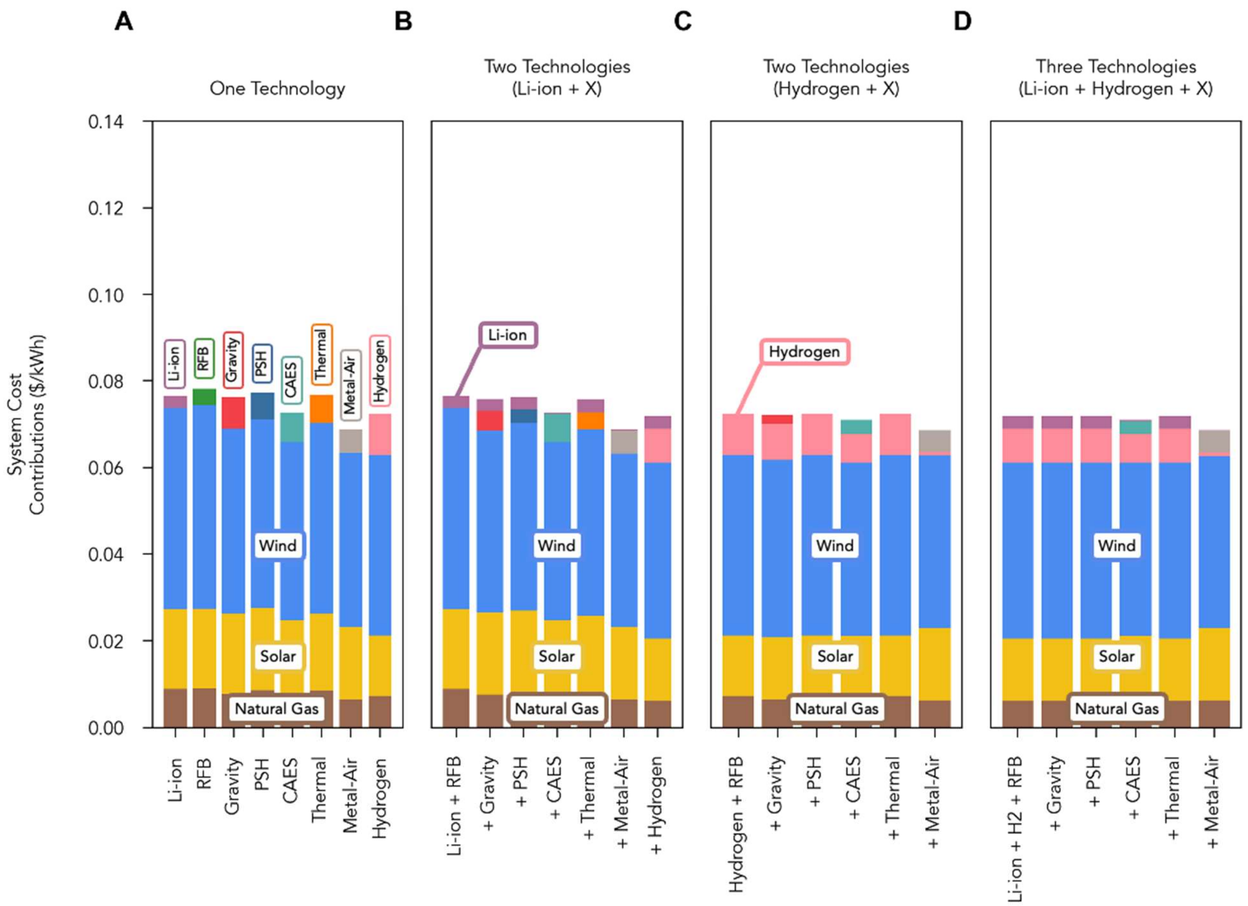

Figure S46. When firm generators were available, the storage capacity required in least-cost systems decreased substantially.

System costs for combinations of short-, mid-, and long-duration storage when 5% constrained natural gas dispatch is included in least-cost systems (see the model formulation section of this document). Natural gas dispatch was constrained to 5% of total dispatch from all generation technologies. Figure S46 displays natural gas cost and efficiency assumptions. System costs when:

(A) Only one storage technology was available: Li-ion batteries, *RFB* (redox-flow batteries), *Gravity* energy storage, *PSH* (Pumped-Storage Hydropower), *CAES* (Compressed Air Energy Storage), *Thermal* energy storage, *Metal-Air* battery storage, or hydrogen energy storage.

(B) Two storage technologies were available: Li-ion batteries, with the second storage technology being a mid-duration storage technology or hydrogen energy storage.

(C) Two storage technologies were available: Hydrogen energy storage, with the second storage technology being a mid-duration storage technology.

(D) Three storage technologies were available: Li-ion batteries and hydrogen energy storage, with the third storage technology being a mid-duration storage technology.

Table S1. Energy-capacity costs and power-capacity costs of energy storage technologies. Ranges of total installed energy- and power-capacity costs of different storage technologies. This table supports Figure 1. Energy-capacity and power-capacity costs were combined to obtain the total cost of Li-ion battery and metal-air battery storage.

Storage Technology		Energy-related Total Overnight Cost ($/kWh)	Power-related Total Overnight Cost ($/kW)		Typical Round-Trip Efficiency (%)	Typical Lifetime (years)	Source
			Charging	Discharging			
**Li-ion Batteries**		326	251	-	86	30	z10
		275 - 373	1220 - 1490	-	83	16	21
		72 - 181	33 - 157	-	85	-	16
**Redox-Flow Batteries (Vanadium)**		200	1412	-	75	30	10
		183 - 306	571 - 808	-	65	12	21
		16 - 104	303 - 541	-	80	-	16
**Pumped-Storage Hydropower**		105	1644	-	81	30	10
		45 - 54	974 - 1644	-	80	60	21
		0	2005	-	80	-	16
**Gravitational Energy Storage**		50 - 194	1181 - 1428	-	80 - 90	35 - 60	21
**Thermal Energy Storage**	Pumped thermal energy storage	38	1703	-	52	30	10

Storage Technology		Energy-related Total Overnight Cost ($/kWh)	Power-related Total Overnight Cost ($/kW)		Typical Round-Trip Efficiency (%)	Typical Lifetime (years)	Source
			Charging	Discharging			
	Different types of thermal energy systems considered: pumped thermal energy storage, sensible heat-based storage, liquid air energy storage	0 - 196	1325 - 1896	-	41 - 60	30 - 35	[21]
	Crushed rock thermal energy storage with a CO_2 power block	3 - 9	3.4	504 - 1251	46	-	[16]
**Compressed Air Energy Storage**	Adiabatic	51	517	774	65	30	[10]
		54	461	629	59	-	[16]
	Diabatic	18	517	774	55	30	[10]
		2 - 15	894 - 1091	-	52	60	[21]
**Metal-air Batteries**		0.1 – 3.7	607 - 969	-	41 - 43	-	[16]
**H_2 power-to-gas-to-power with cavern storage**		2	1706	1415	36	30	[10]
		1.5 - 15	2591 - 3166	-	31	30	[21]
		1.1	489	1214	50	-	[16]

Table S2. Regional average wind and solar capacity factors for 2018.

Region	Region abbreviation	Wind average capacity factor	Solar average capacity factor	Short-hand notation
Contiguous U.S.	CONUS	0.41	0.28	high-wind, high-solar
California Independent System Operator	CAISO	0.22	0.29	low-wind, high-solar
Electric Reliability Council of Texas	ERCOT	0.43	0.26	high-wind, high-solar
Independent System Operator of New England	ISO-NE	0.23	0.20	low-wind, low-solar
Midcontinent Independent System Operator	MISO	0.31	0.21	high-wind, low-solar

Table S3. Costs and assumptions for generation technologies. Assumptions from National Renewable Energy Laboratory's Annual Technology Baseline, 2022. Costs are in 2019 USD and the CO_2 intensity of natural gas was assumed to be 0.461 $kgCO_2/kWh$. In sensitivity studies, natural generation was restricted to various fractions of total generation (natural gas capacity was not restricted).

Generation Technology	Technology Description	Total overnight cost ($/kW)	Fixed O&M ($/kW-yr)	Lifetime (years)	Capital Recovery Factor (%/yr)	Fixed Hourly Cost ($/kW/h)	Variable Cost ($/kWh)
**Solar**	Utility photovoltaics	1391	23	30	8.06	0.015	0
**Wind**	Land-based wind turbines	1436	43	30	8.06	0.018	0
**Solar, ATB 2050 Prediction (Figure S8)**	Utility photovoltaics	521	23	30	8.06	0.007	0
**Wind, ATB 2050 Prediction (Figure S8)**	Land-based wind turbines	850	43	30	8.06	0.013	0
**Natural Gas (Figure S3)**	Natural gas, combined-cycle	1054	27	30	8.06	0.013	0.01

Table S4. Base case costs and assumptions for storage technologies. Unless otherwise noted, values in the same row are taken from the same source. Base case characteristics for Li-ion batteries, redox-flow batteries (RFB), pumped-storage hydropower (PSH), thermal energy storage, compressed air energy storage (CAES), and renewable hydrogen-based power-to-gas-to-power (hydrogen storage) are taken from a peer-reviewed techno-economic review of storage technologies affiliated with NREL and the U.S. Department of Energy (DOE).

Storage Technology		Total overnight cost	Fixed O&M	Fixed property tax, insurance, licensing, permitting	Lifetime	Capital recovery factor	Fixed Hourly Costs	Variable O&M	Decay rate	Round-Trip Efficiency
**Units**		$/kWh for energy<br>$/kW for power	% of capital cost, $/kWh/yr (in the case of energy), or $/kW/yr (in the case of power)	% of capital cost	yrs	%/yr	$/kWh/h for energy<br>$/kW/h for power	$/MWh	-	%
**Li-ion Battery Storage**[26]	Energy	326	4.2%	-	30	8.06	3.957×10^{-3}	0.0031	1% per month	86%
	Power	251	8.5	1.5%						
**RFB (Redox-Flow Batteries)**[26]	Energy	200	1.5%	-	30	8.06	1.867×10^{-3}	-	-	75%
	Power	1412	8.4	1.5%			0.01414	0.0269	-	
**PSH (Pumped-Storage Hydropower)**[26]	Energy	105	1.5%	-	30	8.06	9.820×10^{-4}	-	-	81%
	Power	1644	12.8	1.5%			0.01681	0.0003	-	
**Gravity**[14]	Energy	117	0.58	-	49	7.26	1.033×10^{-3}	-	-	84%
	Power	1416	11.8	-			0.01309	0.5125	-	

Storage Technology		Total overnight cost	Fixed O&M	Fixed property tax, insurance, licensing, permitting	Lifetime	Capital recovery factor	Fixed Hourly Costs	Variable O&M	Decay rate	Round-Trip Efficiency
**Units**		$/kWh for energy<br>$/kW for power	% of capital cost, $/kWh/yr (in the case of energy), or $/kW/yr (in the case of power)	% of capital cost	yrs	%/yr	$/kWh/h for energy<br>$/kW/h for power	$/MWh	-	%
**Thermal**[26]	Energy	38	1.5%	-	30	8.06	3.530×10^{-4}	-	1.5% per day	52%
	Power	1703	13.9	1.5%			0.01749	0.0033		
**A-CAES (Adiabatic Compressed Air Energy Storage)**[26]	Charge Power	517	13.8	1.5%	30	8.06	0.006405	0	-	65%
	Energy	51	1.5%	-			4.770×10^{-4}	-	-	
	Discharge Power	774	13.8	1.5%			0.008805	0.0033	-	
**Metal-Air Battery Storage**[17,32]*	Energy	4.95	0.102	-	30	8.06	0.000214378	-	-	46%
	Power	1505	16.4	-				0	-	
**Hydrogen storage (Hydrogen-based Power-to-gas-to-power)**[26]	Charge Power	1706	13.1	1.5%	30	8.06	0.01742	0.0013	0.1% per day	36%
	Energy	2.0	1.5%	-			1.870×10^{-5}	-		

Storage Technology		Total overnight cost	Fixed O&M	Fixed property tax, insurance, licensing, permitting	Lifetime	Capital recovery factor	Fixed Hourly Costs	Variable O&M	Decay rate	Round-Trip Efficiency
**Units**		$/kWh for energy<br>$/kW for power	% of capital cost, $/kWh/yr (in the case of energy), or $/kW/yr (in the case of power)	% of capital cost	yrs	%/yr	$/kWh/h for energy<br>$/kW/h for power	$/MWh	-	%
	Discharge Power	1415	13.1	1.5%			0.01470	0.0028		
Storage X	Energy	0.01 - 400	1.5%	-	30	8.06	9.339 x 10^{-8} - 0.003736	0	-	86%
	Power	0.01 - 2000	0	1.5%			9.339 x 10^{-8} - 0.01868	0	-	

* The energy- and power-capacity costs provided for metal-air batteries are calculated using a total overnight cost of 20 $/kWh and an assumed 100 h duration, as reported in Form Energy press releases. 4.95 $/kWh was attributed to energy-capacity and 1505 $/kW to power-capacity, based on the ratio of estimated 2020 energy-capacity cost (3.7 $/kWh) to 2020 mid power-capacity cost (1123.53 $/kW) provided in the 2022 MIT Future of Energy Storage report in Table 2.1. **However, it should be noted that with a defined total overnight cost and duration, the total fixed hourly cost calculated remains the same regardless of what costs are attributed to energy-capacity vs. power-capacity.** This is because 1) the calculations for hourly fixed cost are linear operations on overnight fixed cost, and 2) the final fixed hourly cost is a total cost combining energy-capacity and power-capacity fixed hourly cost values. Remaining variables used to calculate the fixed hourly costs are taken from the MIT 2022 Future of Energy Storage report.

Table S5. Optimized discharge times (hours) *Storage X* technologies in the least-cost systems in Figure 2. The optimized discharge time of each storage technology was quantified by its ratio of energy- to power-capacity in the least-cost system.

Table S5a. Region: CONUS				
**Mid-duration Storage Option**	**Storage Technology Portfolio**			
	**Only Mid-duration Storage**	**Short- + Mid-duration Storage**	**Long- + Mid-duration Storage**	**Short- + Mid- + Long-duration Storage**
**Redox-Flow Batteries (RFB)**	20 h	29 h	2 h	Not present
**Pumped-Storage Hydropower (PSH)**	24 h	29 h	4 h	Not present
**Gravity Energy Storage**	24 h	27 h	4 h	Not present
**Thermal Energy Storage**	46 h	74 h	Not present	Not present
**Compressed Air Energy Storage (CAES)**	36 h	37 h	11 h	11 h
**Metal-Air Batteries**	Constrained to a 100 h duration in all simulations due to technological constraints			

Table S5b. Region: CAISO				
**Mid-duration Storage Option**	**Storage Technology Portfolio**			
	**Only Mid-duration Storage**	**Short- + Mid-duration Storage**	**Long- + Mid-duration Storage**	**Short- + Mid- + Long-duration Storage**
**Redox-Flow Batteries (RFB)**	21 h	40 h	4 h	Not present
**Pumped-Storage Hydropower (PSH)**	23 h	32 h	9 h	13 h
**Gravity Energy Storage**	20 h	27 h	9 h	14 h
**Thermal Energy Storage**	51 h	66 h	Not present	Not present
**Compressed Air Energy Storage (CAES)**	31 h	41 h	11 h	19 h
**Metal-Air Batteries**	Constrained to a 100 h duration in all simulations due to technological constraints			

Table S5c. Region: ERCOT				
**Mid-duration Storage Option**	**Storage Technology Portfolio**			
	**Only Mid-duration Storage**	**Short- + Mid-duration Storage**	**Long- + Mid-duration Storage**	**Short- + Mid- + Long-duration Storage**
**Redox-Flow Batteries (RFB)**	24 h	21 h	3 h	Not present
**Pumped-Storage Hydropower (PSH)**	27 h	34 h	6 h	Not present
**Gravity Energy Storage**	26 h	32 h	6 h	14 h
**Thermal Energy Storage**	101 h	125 h	11 h	Not present
**Compressed Air Energy Storage (CAES)**	71 h	87 h	13 h	15 h
**Metal-Air Batteries**	Constrained to a 100 h duration in all simulations due to technological constraints			

Table S5d. Region: ISO-NE				
**Mid-duration Storage Option**	**Storage Technology Portfolio**			
	**Only Mid-duration Storage**	**Short- + Mid-duration Storage**	**Long- + Mid-duration Storage**	**Short- + Mid- + Long-duration Storage**
**Redox-Flow Batteries (RFB)**	34 h	74 h	3 h	Not present
**Pumped-Storage Hydropower (PSH)**	33 h	59 h	9 h	15 h
**Gravity Energy Storage**	32 h	57 h	8 h	13 h
**Thermal Energy Storage**	46 h	107 h	12 h	Not present
**Compressed Air Energy Storage (CAES)**	33 h	59 h	15 h	34 h
**Metal-Air Batteries**	Constrained to a 100 h duration in all simulations due to technological constraints			

Table S5e. Region: MISO				
**Mid-duration Storage Option**	**Storage Technology Portfolio**			
	**Only Mid-duration Storage**	**Short- + Mid-duration Storage**	**Long- + Mid-duration Storage**	**Short- + Mid- + Long-duration Storage**
**Redox-Flow Batteries (RFB)**	18 h	27 h	1 h	Not present
**Pumped-Storage Hydropower (PSH)**	30 h	45 h	7 h	Not present
**Gravity Energy Storage**	29 h	44 h	4 h	Not present
**Thermal Energy Storage**	40 h	63 h	8 h	Not present
**Compressed Air Energy Storage (CAES)**	35 h	48 h	10 h	11 h
**Metal-Air Batteries**	Constrained to a 100 h duration in all simulations due to technological constraints			

Table S6. Equivalent annual discharge cycles (cycles/year) of *Storage X* technologies in the least-cost systems in Figure 3. The equivalent annual discharge cycles of each storage technology was quantified by its total annual storage discharge divided by its deployed energy-capacity in the least-cost system.

Table S6a. Region: CONUS				
**Mid-duration Storage Option**	**Storage Technology Portfolio**			
	**Only Mid-duration Storage**	**Short- + Mid-duration Storage**	**Long- + Mid-duration Storage**	**Short- + Mid- + Long-duration Storage**
**Redox-Flow Batteries (RFB)**	13 cycles/yr	8 cycles/yr	365 cycles/yr	Not present
**Pumped-Storage Hydropower (PSH)**	14 cycles/yr	14 cycles/yr	294 cycles/yr	Not present
**Gravity Energy Storage**	14 cycles/yr	11 cycles/yr	259 cycles/yr	Not present
**Thermal Energy Storage**	9 cycles/yr	6 cycles/yr	Not present	Not present
**Compressed Air Energy Storage (CAES)**	9 cycles/yr	9 cycles/yr	88 cycles/yr	84 cycles/yr
**Metal-Air Batteries**	6 cycles/yr	7 cycles/yr	14 cycles/yr	15 cycles/yr

Table S6b. Region: CAISO				
**Mid-duration Storage Option**	**Storage Technology Portfolio**			
	**Only Mid-duration Storage**	**Short- + Mid-duration Storage**	**Long- + Mid-duration Storage**	**Short- + Mid- + Long-duration Storage**
**Redox-Flow Batteries (RFB)**	55 cycles/yr	9 cycles/yr	279 cycles/yr	Not present
**Pumped-Storage Hydropower (PSH)**	47 cycles/yr	58 cycles/yr	263 cycles/yr	208 cycles/yr
**Gravity Energy Storage**	52 cycles/yr	40 cycles/yr	279 cycles/yr	190 cycles/yr
**Thermal Energy Storage**	51 cycles/yr	66 cycles/yr	Not present	Not present
**Compressed Air Energy Storage (CAES)**	28 cycles/yr	22 cycles/yr	184 cycles/yr	151 cycles/yr
**Metal-Air Batteries**	11 cycles/yr	13 cycles/yr	10 cycles/yr	15 cycles/yr

Table S6c. Region: ERCOT				
**Mid-duration Storage Option**	**Storage Technology Portfolio**			
	**Only Mid-duration Storage**	**Short- + Mid-duration Storage**	**Long- + Mid-duration Storage**	**Short- + Mid- + Long-duration Storage**
**Redox-Flow Batteries (RFB)**	34 cycles/yr	7 cycles/yr	334 cycles/yr	Not present
**Pumped-Storage Hydropower (PSH)**	25 cycles/yr	24 cycles/yr	225 cycles/yr	Not present
**Gravity Energy Storage**	26 cycles/yr	20 cycles/yr	154 cycles/yr	81 cycles/yr
**Thermal Energy Storage**	8 cycles/yr	7 cycles/yr	125 cycles/yr	Not present
**Compressed Air Energy Storage (CAES)**	9 cycles/yr	8 cycles/yr	72 cycles/yr	62 cycles/yr
**Metal-Air Batteries**	6 cycles/yr	7 cycles/yr	13 cycles/yr	14 cycles/yr

Table S6d. Region: ISO-NE				
**Mid-duration Storage Option**	**Storage Technology Portfolio**			
	**Only Mid-duration Storage**	**Short- + Mid-duration Storage**	**Long- + Mid-duration Storage**	**Short- + Mid- + Long-duration Storage**
**Redox-Flow Batteries (RFB)**	27 cycles/yr	12 cycles/yr	245 cycles/yr	Not present
**Pumped-Storage Hydropower (PSH)**	30 cycles/yr	30 cycles/yr	188 cycles/yr	129 cycles/yr
**Gravity Energy Storage**	32 cycles/yr	57 cycles/yr	187 cycles/yr	138 cycles/yr
**Thermal Energy Storage**	24 cycles/yr	9 cycles/yr	73 cycles/yr	Not present
**Compressed Air Energy Storage (CAES)**	24 cycles/yr	15 cycles/yr	79 cycles/yr	63 cycles/yr
**Metal-Air Batteries**	10 cycles/yr	11 cycles/yr	12 cycles/yr	12 cycles/yr

Table S6e. Region: MISO				
**Mid-duration Storage Option**	**Storage Technology Portfolio**			
	**Only Mid-duration Storage**	**Short- + Mid-duration Storage**	**Long- + Mid-duration Storage**	**Short- + Mid- + Long-duration Storage**
**Redox-Flow Batteries (RFB)**	12 cycles/yr	6 cycles/yr	291 cycles/yr	Not present
**Pumped-Storage Hydropower (PSH)**	15 cycles/yr	15 cycles/yr	204 cycles/yr	Not present
**Gravity Energy Storage**	16 cycles/yr	9 cycles/yr	181 cycles/yr	Not present
**Thermal Energy Storage**	11 cycles/yr	7 cycles/yr	137 cycles/yr	Not present
**Compressed Air Energy Storage (CAES)**	21 cycles/yr	15 cycles/yr	68 cycles/yr	55 cycles/yr
**Metal-Air Batteries**	8 cycles/yr	11 cycles/yr	12 cycles/yr	12 cycles/yr

Table S7. Least-cost system results when various single storage technologies are available. Results correspond to the cases shown in Figure 2b and Figure 3b.

	Table S7a. Region: CONUS								
	Units	Single Storage Technologies							
		Li- ion	RFB	Gravity	PSH	CAES	Thermal	Metal -Air	Hydrog en
**Total System Cost**	$/kWh	0.133	0.131	0.113	0.116	0.103	0.114	0.089	0.084
**Percent Reduction in Total System cost (compared to a least-cost system with no storage capacity relying on wind- and solar generation)**	%	55.4	56.0	62.1	61.1	65.4	61.7	70.1	71.8
**To Storage Power Capacity**	kW	-	0.74	0.784	0.779	0.554	0.749	-	0.198
**Storage Energy Capacity**	kWh	4.38	14.98	18.76	18.58	28.38	34.34	89.56	368.04
**From Storage Power Capacity**	kW	-	-	-	-	0.791	-	-	0.879
**Storage Optimal Discharge Time**	h	4	20.24	23.92	23.84	35.88	45.82	100	418.77
**Storage Equivalent Annual Cycles**	cycles/yr	11.74	12.70	14.44	14.26	9.26	9.37	5.89	1.74

	Units	Single Storage Technologies							
		Li-ion	RFB	Gravity	PSH	CAES	Thermal	Metal-Air	Hydrogen
**Total System Cost**	$/kWh	0.178	0.187	0.155	0.161	0.143	0.169	0.123	0.129
**Percent Reduction in Total System cost (compared to a least-cost system with no storage capacity relying on wind- and solar generation)**	%	95.43	95.20	96.02	95.86	96.33	95.66	96.84	96.69
**To Storage Power Capacity**	kW	-	1.139	1.139	1.132	0.827	1.055	-	0.374
**Storage Energy Capacity**	kWh	13.91	24.31	23.34	25.63	34.93	53.61	124.63	574.13
**From Storage Power Capacity**	kW	-	-	-	-	1.132	-	-	1.124
**Storage Optimal Discharge Time**	h	4	21.35	20.50	22.63	30.86	50.81	100	510.91
**Storage Equivalent Annual Cycles**	cycles/yr		55.46	52.14	46.82	28.04	19.94	11.43	1.73

Table S7b. Region: CAISO

	Table S7c. Region: ERCOT								
	Units	Single Storage Technologies							
		Li-ion	RFB	Gravity	PSH	CAES	Thermal	Metal-Air	Hydrogen
**Total System Cost**	$/kWh	0.202	0.216	0.171	0.178	0.142	0.166	0.106	0.105
**Percent Reduction in Total System cost (compared to a least-cost system with no storage capacity relying on wind- and solar generation)**	%	86.69	85.77	88.73	88.27	90.64	89.06	93.02	93.08
**To Storage Power Capacity**	kW	-	1.392	1.389	1.388	0.576	1.368	-	0.379
**Storage Energy Capacity**	kWh	16.60	33.69	36.14	37.74	98.19	137.96	138.61	630.35
**From Storage Power Capacity**	kW	-	-	-	-	1.388	-	-	1.388
**Storage Optimal Discharge Time**	h	4	24.20	26.01	27.18	70.74	100.88	100	454.07
**Storage Equivalent Annual Cycles**	cycles/yr	38.43	33.61	26.36	25.46	8.86	8.17	6.26	1.69

	Table S7d. Region: ISO-NE								
	Units	Single Storage Technologies							
		Li-ion	RFB	Gravity	PSH	CAES	Thermal	Metal-Air	Hydrogen
**Total System Cost**	$/kWh	0.275	0.253	0.2	0.206	0.18	0.22	0.149	0.158
**Percent Reduction in Total System cost (compared to a least-cost system with no storage capacity relying on wind- and solar generation)**	%	97.48	97.68	98.17	98.11	98.35	97.98	98.63	98.55
**To Storage Power Capacity**	kW	-	1.365	1.402	1.399	1.239	1.329	-	0.714
**Storage Energy Capacity**	kWh	24.48	46.51	45.33	46.58	46.66	60.65	143.55	677.78
**From Storage Power Capacity**	kW	-	-	-	-	1.398	-	-	1.416
**Storage Optimal Discharge Time**	h	4	34.07	32.33	33.23	33.36	45.62	100	478.66
**Storage Equivalent Annual Cycles**	cycles/yr	21.7	26.68	30.26	29.97	24.14	24.33	9.82	1.959

		Table S7e. Region: MISO							
	Units	Single Storage Technologies							
		Li-ion	RFB	Gravity	PSH	CAES	Thermal	Metal-Air	Hydrogen
**Total System Cost**	$/kWh	0.162	0.163	0.14	0.143	0.124	0.14	0.106	0.105
**Percent Reduction in Total System cost (compared to a least-cost system with no storage capacity relying on wind- and solar generation)**	%	55.89	55.62	61.88	61.07	66.24	61.88	71.14	71.41
**To Storage Power Capacity**	kW	-	0.826	0.969	0.962	0.729	0.899	-	0.241
**Storage Energy Capacity**	kWh	8.55	14.64	28.36	28.66	37.09	35.55	107.62	354.17
**From Storage Power Capacity**	kW	-	-	-	-	1.063	-	-	1.025
**Storage Optimal Discharge Time**	h	4	17.73	29.27	29.78	34.91	39.54	100	345.68
**Storage Equivalent Annual Cycles**	cycles/yr	11.70	11.70	15.50	15.06	20.73	11.42	7.98	1.94

Table S8. Least-cost system results when two storage technologies were available: Li-ion batteries, and the second storage technology being a mid-duration storage technology or hydrogen energy storage. Results correspond to the cases shown in Figure 2c and Figure 3c.

	Table S8a. Region: CONUS							
	Units	Second Storage Technology Available Alongside Li-ion Battery Storage						
		RFB	Gravity	PSH	CAES	Thermal	Metal-Air	Hydrogen
Total System Cost	$/kWh	0.127	0.113	0.115	0.103	0.111	0.089	0.083
Percent Reduction in Total System cost (compared to a least-cost system with no storage capacity relying on wind- and solar generation)	%	57.4	62.1	61.4	65.4	62.8	70.1	72.1
Li-ion Battery Capacity	kW	1.14	0.558	1.01	0.096	1.01	0.703	0.714
To 2nd Storage Power Capacity	kW	0.454	0.641	0.51	0.546	0.511	-	0.179
2nd Storage Energy Capacity	kWh	12.99	17.38	14.65	28.40	37.95	72.59	371.95
From 2nd Storage Power Capacity	kW	-	-	-	0.768	-	-	0.712
2nd Storage Optimal Discharge Time	h	28.61	27.12	28.75	37.00	74.28	100.00	522.20
Li-ion Battery Equivalent Annual Cycles	cycles/yr	59.34	104.24	8.78	141.34	73.47	37.54	232.38
2nd Storage Equivalent Annual Cycles	cycles/yr	7.84	10.96	14.23	8.83	6.25	7.38	1.55

	Table S8b. Region: CAISO							
	**Units**	**Second Storage Technology Available Alongside Li-ion Battery Storage**						
		**RFB**	**Gravity**	**PSH**	**CAES**	**Thermal**	**Metal-Air**	**Hydrogen**
**Total System Cost**	$/kWh	0.173	0.153	0.156	0.141	0.155	0.123	0.124
**Percent Reduction in Total System cost (compared to a least-cost system with no storage capacity relying on wind- and solar generation)**	%	95.56	96.07	95.99	96.38	96.02	96.84	96.81
**Li-ion Battery Capacity**	kW	7.92	1.47	2.86	1.32	4.38	1.07	3.07
**To 2nd Storage Power Capacity**	kW	0.277	0.788	0.637	0.641	0.494	-	0.283
**2nd Storage Energy Capacity**	kWh	11.01	21.18	20.14	32.99	32.73	97.86	533.69
**From 2nd Storage Power Capacity**	kW	-	-	-	0.807	-	-	0.658
**2nd Storage Optimal Discharge Time**	h	39.75	26.89	31.62	40.90	66.31	100	811.63
**Li-ion Battery Equivalent Annual Cycles**	cycles/yr	125.70	221.42	41.05	220.17	126.62	141.90	292.41
**2nd Storage Equivalent Annual Cycles**	cycles/yr	9.16	40.24	57.75	22.08	12.32	13.03	1.40

		Table S8c. Region: ERCOT						
	Units	Second Storage Technology Available Alongside Li-ion Battery Storage						
		RFB	Gravity	PSH	CAES	Thermal	Metal-Air	Hydrogen
**Total System Cost**	$/kWh	0.197	0.169	0.174	0.142	0.16	0.104	0.102
**Percent Reduction in Total System cost (compared to a least-cost system with no storage capacity relying on wind- and solar generation)**	%	87.02	88.86	88.53	90.64	89.46	93.15	93.28
**Li-ion Battery Capacity**	kW	8.38	1.13	1.28	1.10	1.46	1.16	1.12
**To 2nd Storage Power Capacity**	kW	0.549	1.106	1.086	0.532	1.03	-	0.312
**2nd Storage Energy Capacity**	kWh	11.32	35.08	37.37	96.72	129.21	109.85	581.26
**From 2nd Storage Power Capacity**	kW	-	-	-	1.117	-	-	1.11
**2nd Storage Optimal Discharge Time**	h	20.62	31.72	34.41	86.58	125.44	100	523.49
**Li-ion Battery Equivalent Annual Cycles**	cycles/yr	69.82	174.62	13.18	195.04	139.52	63.33	211.21
**2nd Storage Equivalent Annual Cycles**	cycles/yr	6.66	20.28	24.40	7.50	6.81	7.45	1.54

	Units	Second Storage Technology Available Alongside Li-ion Battery Storage						
**Table S8d. Region: ISO-NE**		RFB	Gravity	PSH	CAES	Thermal	Metal-Air	Hydrogen
**Total System Cost**	$/kWh	0.244	0.198	0.201	0.177	0.196	0.148	0.15
**Percent Reduction in Total System cost (compared to a least-cost system with no storage capacity relying on wind- and solar generation)**	%	97.76	98.18	98.16	98.38	98.20	98.64	98.62
**Li-ion Battery Capacity**	kW	5.03	2.63	2.70	2.61	5.10	0.99	2.63
**To 2nd Storage Power Capacity**	kW	0.596	0.752	0.743	0.899	0.547	-	0.564
**2nd Storage Energy Capacity**	kWh	44.35	42.79	44.10	44.30	58.75	121.86	662.38
**From 2nd Storage Power Capacity**	kW	-	-	-	0.752	-	-	0.787
**2nd Storage Optimal Discharge Time**	h	74.38	56.88	59.32	58.91	107.41	100	841.74
**Li-ion Battery Equivalent Annual Cycles**	cycles/yr	151.04	199.04	41.62	195.87	129.78	139.19	223.84
**2nd Storage Equivalent Annual Cycles**	cycles/yr	12.30	18.80	30.19	14.96	9.18	10.60	1.55

	Units	Table S8e. Region: MISO						
		Second Storage Technology Available Alongside Li-ion Battery Storage						
		RFB	Gravity	PSH	CAES	Thermal	Metal-Air	Hydrogen
**Total System Cost**	$/kWh	0.157	0.139	0.14	0.123	0.134	0.104	0.103
**Percent Reduction in Total System cost (compared to a least-cost system with no storage capacity relying on wind- and solar generation)**	%	57.25	62.16	61.88	66.51	63.52	71.68	71.96
**Li-ion Battery Capacity**	kW	1.68	1.45	1.45	1.29	1.48	1.31	1.41
**To 2nd Storage Power Capacity**	kW	0.426	0.605	0.595	0.516	0.558	-	0.207
**2nd Storage Energy Capacity**	kWh	11.44	26.56	26.80	35.53	35.23	75.32	332.05
**From 2nd Storage Power Capacity**	kW	-	-	-	0.747	-	-	0.684
**2nd Storage Optimal Discharge Time**	h	26.83	43.94	45.06	47.56	63.17	100	485.68
**Li-ion Battery Equivalent Annual Cycles**	cycles/yr	45.46	109.88	14.54	206.51	86.36	62.97	166.24
**2nd Storage Equivalent Annual Cycles**	cycles/yr	6.30	9.17	14.84	15.19	7.40	10.64	1.734

Table S9. Least-cost system results when two storage technologies were available: Hydrogen energy storage, and the second storage technology being a mid-duration storage technology. Results correspond to the cases shown in Figure 2d and Figure 3d.

	Table S9a. Region: CONUS						
	Units	Second Storage Technology Available Alongside Hydrogen Energy Storage					
		RFB	Gravity	PSH	CAES	Therma l	Metal-Air
**Total System Cost**	$/kWh	0.084	0.084	0.084	0.083	0.084	0.083
**Percent Reduction in Total System cost (compared to a least-cost system with no storage capacity relying on wind- and solar generation)**	%	71.8	71.8	71.8	72.1	71.8	72.1
**To 2nd Storage Power Capacity**	kW	0.029	0.163	0.043	0.095	0	-
**2nd Storage Energy Capacity**	kWh	0.052	0.583	0.191	2.641	0	38.922
**From 2nd Storage Power Capacity**	kW	-	-	-	0.25	-	-
**Electrolyzer Power Capacity**	kW	0.195	0.181	0.189	0.165	0.198	0.079
**Hydrogen Energy Storage Capacity**	kWh	367	368	363	375	368	321
**Fuel Cell Power Capacity**	kW	0.851	0.723	0.839	0.643	0.879	0.512
**2nd Storage Optimal Discharge Time**	h	1.807	3.567	4.424	10.577	0	100
**Hydrogen Optimal Discharge Time**	h	430.84	508.81	432.59	583.30	418.77	627.72
**2nd Storage Equivalent Annual Cycles**	cycles/yr	365.08	258.55	294.15	88.04	0	14.03
**Hydrogen Equivalent Annual Cycles**	cycles/yr	1.72	1.58	1.70	1.39	1.74	0.853

	Units	Second Storage Technology Available Alongside Hydrogen Energy Storage					
		**Table S9b. Region: CAISO**					
		RFB	Gravity	PSH	CAES	Therma l	Metal-Air
Total System Cost	$/kWh	0.129	0.124	0.127	0.122	0.129	0.122
Percent Reduction in Total System cost (compared to a least-cost system with no storage capacity relying on wind- and solar generation)	%	96.69	96.81	96.74	96.87	96.69	96.87
To 2nd Storage Power Capacity	kW	0.227	0.870	0.506	0.777	0	-
2nd Storage Energy Capacity	kWh	0.81	7.65	4.34	9.34	0	106.51
From 2nd Storage Power Capacity	kW	-	-	-	0.883	-	-
Electrolyzer Power Capacity	kW	0.344	0.199	0.251	0.189	0.374	0.027
Hydrogen Energy Storage Capacity	kWh	570.01	477.33	520.59	481.26	574.13	101.73
Fuel Cell Power Capacity	kW	0.911	0.466	0.727	0.439	1.124	0.111
2nd Storage Optimal Discharge Time	h	3.57	8.79	8.58	10.58	0	100
Hydrogen Optimal Discharge Time	h	625.38	1025.02	716.10	1097.3 8	510.91	920.44
2nd Storage Equivalent Annual Cycles	cycles/yr	279.25	278.82	263.06	183.63	0	10.35
Hydrogen Equivalent Annual Cycles	cycles/yr	1.57	1.17	1.30	1.10	1.73	0.76

	Units	Table S9c. Region: ERCOT					
		**Second Storage Technology Available Alongside Hydrogen Energy Storage**					
		**RFB**	**Gravity**	**PSH**	**CAES**	**Thermal**	**Metal-Air**
Total System Cost	$/kWh	0.105	0.103	0.104	0.101	0.105	0.1
Percent Reduction in Total System cost (compared to a least-cost system with no storage capacity relying on wind- and solar generation)	%	93.08	93.21	93.15	93.34	93.08	93.41
To 2nd Storage Power Capacity	kW	0.103	0.333	0.185	0.262	0.008	-
2nd Storage Energy Capacity	kWh	0.29	2.11	1.17	7.64	0.09	70.66
From 2nd Storage Power Capacity	kW	-	-	-	0.602	-	-
Electrolyzer Power Capacity	kW	0.363	0.306	0.328	0.243	0.376	0.125
Hydrogen Energy Storage Capacity	kWh	623.38	572.91	603.87	572.66	629.38	455.46
Fuel Cell Power Capacity	kW	1.286	1.057	1.205	0.789	1.38	0.686
2nd Storage Optimal Discharge Time	h	2.81	6.33	6.33	12.68	10.97	100
Hydrogen Optimal Discharge Time	h	484.83	541.83	501.15	725.91	456.00	663.96
2nd Storage Equivalent Annual Cycles	cycles/yr	333.82	153.88	224.52	72.27	124.72	13.39
Hydrogen Equivalent Annual Cycles	cycles/yr	1.63	1.49	1.54	1.23	1.69	0.78

	Units	Table S9d. Region: ISO-NE<br>Second Storage Technology Available Alongside Hydrogen Energy Storage					
		**RFB**	**Gravity**	**PSH**	**CAES**	**Therma l**	**Metal-Air**
Total System Cost	$/kWh	0.157	0.151	0.155	0.149	0.158	0.146
Percent Reduction in Total System cost (compared to a least-cost system with no storage capacity relying on wind- and solar generation)	%	98.56	98.61	98.58	98.63	98.55	98.66
To 2nd Storage Power Capacity	kW	0.426	0.8	0.586	0.631	0.064	-
2nd Storage Energy Capacity	kWh	1.27	6.68	5.11	14.09	0.77	123.82
From 2nd Storage Power Capacity	kW	-	-	-	0.911	-	-
Electrolyzer Power Capacity	kW	0.672	0.442	0.507	0.390	0.697	0.126
Hydrogen Energy Storage Capacity	kWh	682.32	658.76	664.54	646.20	681.25	407.23
Fuel Cell Power Capacity	kW	0.999	0.663	0.864	0.550	1.354	0.203
2nd Storage Optimal Discharge Time	h	2.97	8.35	8.72	15.46	12.06	100
Hydrogen Optimal Discharge Time	h	682.79	993.45	769.39	1175.4 9	503.32	2006.55
2nd Storage Equivalent Annual Cycles	cycles/yr	244.72	187.40	188.14	79.49	73.26	11.77
Hydrogen Equivalent Annual Cycles	cycles/yr	1.74	1.25	1.37	1.10	1.93	0.77

	Table S9e. Region: MISO						
	Units	Second Storage Technology Available Alongside Hydrogen Energy Storage					
		**RFB**	**Gravity**	**PSH**	**CAES**	**Thermal**	**Metal-Air**
Total System Cost	$/kWh	0.105	0.104	0.105	0.102	0.105	0.102
Percent Reduction in Total System cost (compared to a least-cost system with no storage capacity relying on wind- and solar generation)	%	71.41	71.68	71.41	72.23	71.41	72.23
To 2nd Storage Power Capacity	kW	0.155	0.331	0.120	0.160	0.005	-
2nd Storage Energy Capacity	kWh	0.21	1.28	0.79	4.88	0.004	55.53
From 2nd Storage Power Capacity	kW	-	-	-	0.507	-	-
Electrolyzer Power Capacity	kW	0.231	0.215	0.225	0.189	0.240	0.092
Hydrogen Energy Storage Capacity	kWh	343.59	334.43	336.19	334.12	352.96	285.58
Fuel Cell Power Capacity	kW	0.872	0.705	0.915	0.537	1.019	0.497
2nd Storage Optimal Discharge Time	h	1.36	3.87	6.64	9.62	7.98	100
Hydrogen Optimal Discharge Time	h	394.01	474.64	367.49	622.27	346.25	574.14
2nd Storage Equivalent Annual Cycles	cycles/yr	291.37	181.38	204.29	67.68	137.36	12.21
Hydrogen Equivalent Annual Cycles	cycles/yr	1.91	1.77	1.86	1.54	1.94	0.76

Table S10. Least-cost system results when three storage technologies were available: Li-ion batteries, hydrogen energy storage, and the third storage technology being a mid-duration storage technology. Results correspond to the cases shown in Figure 2e and Figure 3e.

	Table S10a. Region: CONUS						
	Units	Third Storage Technology Available Alongside Li-ion Batteries and Hydrogen Energy Storage					
		RFB	Gravity	PSH	CAES	Thermal	Metal-Air
**Total System Cost**	$/kWh	0.083	0.083	0.083	0.083	0.083	0.082
**% Reduction from least-cost system with no storage capacity**	%	72.1	72.1	72.1	72.1	72.1	72.5
**Li-ion Battery Capacity**	kWh	0.714	0.714	0.714	0.114	0.714	0.717
**To 3rd Storage Power Capacity**	kW	0	0	0	0.085	0	-
**3rd Storage Energy Capacity**	kWh	0	0	0	2.435	0	31.222
**From 3rd Storage Power Capacity**	kW	-	-	-	0.221	-	-
**Electrolyzer Power Capacity**	kW	0.179	0.179	0.179	0.165	0.179	0.091
**Hydrogen Energy Storage Capacity**	kWh	371.95	371.95	371.95	375.53	371.95	346.50
**Fuel Cell Power Capacity**	kW	0.712	0.712	0.712	0.644	0.712	0.421
**3rd Storage Optimal Discharge Time**	h	0	0	0	11.027	0	100
**Hydrogen Optimal Discharge Time**	h	522.20	522.20	522.20	582.81	522.20	823.48
**Li-ion Battery Equivalent Annual Cycles**	cycles/yr	232.38	232.38	232.38	293.76	232.38	208.67
**3rd Storage Equivalent Annual Cycles**	cycles/yr	0	0	0	83.906	0	14.983
**Hydrogen Equivalent Annual Cycles**	cycles/yr	1.55	1.55	1.55	1.39	1.55	0.87

	Units	Table S10b. Region: CAISO<br>Third Storage Technology Available Alongside Li-ion Batteries and Hydrogen Energy Storage					
		RFB	Gravity	PSH	CAES	Thermal	Metal-Air
Total System Cost	$/kWh	0.124	0.124	0.122	0.121	0.124	0.119
% Reduction from least-cost system with no storage capacity	%	96.81	96.81	96.87	96.89	96.81	96.94
Li-ion Battery Capacity	kWh	3.07	1.91	1.99	1.99	3.07	1.70
To 3rd Storage Power Capacity	kW	0	0.177	0.318	0.495	0	-
3rd Storage Energy Capacity	kWh	0	2.30	4.59	7.24	0	61.86
From 3rd Storage Power Capacity	kW	-	-	-	0.41	-	-
Electrolyzer Power Capacity	kW	0.283	0.262	0.215	0.187	0.283	0.055
Hydrogen Energy Storage Capacity	kWh	533.69	530.84	496.94	470.00	533.69	196.90
Fuel Cell Power Capacity	kW	0.658	0.604	0.475	0.425	0.658	0.175
3rd Storage Optimal Discharge Time	h	0	13.01	14.43	17.67	0	100
Hydrogen Optimal Discharge Time	h	811.63	879.45	1045.34	1106.43	811.63	1127.00
Li-ion Battery Equivalent Annual Cycles	cycles/yr	292.41	310.95	331.35	340.34	292.41	204.54
3rd Storage Equivalent Annual Cycles	cycles/yr	0	207.75	189.97	150.81	0	15.04
Hydrogen Equivalent Annual Cycles	cycles/yr	1.40	1.31	1.19	1.11	1.40	0.82

	Table S10c. Region: ERCOT						
	Units	Third Storage Technology Available Alongside Li-ion Batteries and Hydrogen Energy Storage					
		RFB	Gravity	PSH	CAES	Thermal	Metal-Air
Total System Cost	$/kWh	0.102	0.102	0.102	0.101	0.102	0.099
% Reduction from least-cost system with no storage capacity	%	93.28	93.28	93.28	93.34	93.28	93.48
Li-ion Battery Capacity	kWh	1.12	1.12	1.11	0.67	1.12	1.04
To 3rd Storage Power Capacity	kW	0	0	0.051	0.21	0	-
3rd Storage Energy Capacity	kWh	0	0	0.73	6.83	0	57.76
From 3rd Storage Power Capacity	kW	-	-	-	0.24	-	-
Electrolyzer Power Capacity	kW	0.312	0.312	0.301	0.240	0.312	0.130
Hydrogen Energy Storage Capacity	kWh	581.26	581.26	577.59	567.89	581.26	454.19
Fuel Cell Power Capacity	kW	1.11	1.11	1.062	0.775	1.11	0.557
3rd Storage Optimal Discharge Time	h	0	0	14.29	15.17	0	100
Hydrogen Optimal Discharge Time	h	523.49	523.49	543.90	733.12	523.49	815.42
Li-ion Battery Equivalent Annual Cycles	cycles/yr	211.21	211.21	237.56	263.07	211.21	213.38
3rd Storage Equivalent Annual Cycles	cycles/yr	0	0	80.75	61.73	0	13.60
Hydrogen Equivalent Annual Cycles	cycles/yr	1.54	1.54	1.50	1.23	1.54	0.82

	Table S10d. Region: ISO-NE						
	Units	Third Storage Technology Available Alongside Li-ion Batteries and Hydrogen Energy Storage					
		RFB	Gravity	PSH	CAES	Thermal	Metal-Air
Total System Cost	$/kWh	0.15	0.15	0.149	0.148	0.15	0.144
% Reduction from least-cost system with no storage capacity	%	98.62	98.62	98.63	98.64	98.62	98.68
Li-ion Battery Capacity	kWh	2.63	2.61	2.41	2.46	2.63	2.00
To 3rd Storage Power Capacity	kW	0	0.087	0.151	0.387	0	-
3rd Storage Energy Capacity	kWh	0	1.28	1.90	10.15	0	74.08
From 3rd Storage Power Capacity	kW	-	-	-	0.294	-	-
Electrolyzer Power Capacity	kW	0.564	0.526	0.514	0.377	0.564	0.192
Hydrogen Energy Storage Capacity	kWh	662.38	660.44	660.83	629.37	662.38	490.41
Fuel Cell Power Capacity	kW	0.787	0.709	0.7	0.563	0.787	0.215
3rd Storage Optimal Discharge Time	h	0	14.60	12.61	34.45	0	100
Hydrogen Optimal Discharge Time	h	841.74	932.10	943.51	1118.70	841.74	2276.43
Li-ion Battery Equivalent Annual Cycles	cycles/yr	223.84	230.49	239.59	263.35	223.84	277.14
3rd Storage Equivalent Annual Cycles	cycles/yr	0	129.24	137.56	62.63	0	12.46
Hydrogen Equivalent Annual Cycles	cycles/yr	1.55	1.44	1.40	1.13	1.55	0.84

	Table S10e. Region: MISO						
	Units	Third Storage Technology Available Alongside Li-ion Batteries and Hydrogen Energy Storage					
		RFB	Gravity	PSH	CAES	Thermal	Metal-Air
Total System Cost	$/kWh	0.103	0.103	0.103	0.102	0.103	0.101
% Reduction from least-cost system with no storage capacity	%	71.96	71.96	71.96	72.23	71.96	72.50
Li-ion Battery Capacity	kWh	1.41	1.41	1.41	0.41	1.41	1.36
To 3rd Storage Power Capacity	kW	0	0	0	0.122	0	-
3rd Storage Energy Capacity	kWh	0	0	0	4.62	0	46.53
From 3rd Storage Power Capacity	kW	-	-	-	0.416	-	-
Electrolyzer Power Capacity	kW	0.207	0.207	0.207	0.184	0.207	0.075
Hydrogen Energy Storage Capacity	kWh	332.05	332.05	332.05	331.20	332.05	224.96
Fuel Cell Power Capacity	kW	0.684	0.684	0.684	0.525	0.684	0.267
3rd Storage Optimal Discharge Time	h	0	0	0	11.12	0	100
Hydrogen Optimal Discharge Time	h	485.68	485.68	485.68	631.23	485.68	843.87
Li-ion Battery Equivalent Annual Cycles	cycles/yr	166.24	166.24	166.24	246.72	166.24	170.16
3rd Storage Equivalent Annual Cycles	cycles/yr	0	0	0	55.03	0	12.22
Hydrogen Equivalent Annual Cycles	cycles/yr	1.73	1.73	1.73	1.52	1.73	0.86

Model formulation

The model nomenclature referenced in this section is presented in Table S11.

Objective Function

$$minimize(system\ cost)$$

$$system\ cost = \sum_{g} c_{fixed}^{g} C^{g} + \sum_{g} \left(\frac{\Sigma_t\, c_{var}^{g}\, D_t^{g}}{T} \right) + \sum_{v} c_{fixed}^{v} C^{v}$$

$$+ \sum_{s} c_{fixed}^{s} C^{s} + \frac{\sum_{t} c_{var}^{to\ s}\, D_t^{s}}{T} + \frac{\sum_{t} c_{var}^{from\ s}\, D_t^{s}}{T}$$

.

$$c_{fixed}^{g,v} = \frac{\gamma c_{capital}^{g,v} + c_{fixed\ O\&M}^{g,v}}{h}$$

$$\gamma = \frac{i(1+i)^n}{(1+i)^n - 1}$$

Constraints

Capacity:

$$0 \le C^{g,v,s} \ \ \forall g, v, s$$

Dispatch:

$$0 \le D_t^{g} \le C^{g} f^{g} \quad \forall g, t$$

$$0 \le D_t^{v} \le C^{v} \quad \forall g, t$$

$$0 \le D_t^{to\ s} \le \frac{C^{s}}{\tau^{s}} \quad \forall g, t$$

$$0 \le D_t^{from\ s} \le \frac{C^{s}}{\tau^{s}} \quad \forall g, t$$

$$0 \le S_t^{s} \le C^{s} \quad \forall g, t$$

$$0 \le D^{from\ s_t} \le S_t^{s}(1 - \delta^{s}) \quad \forall g, t$$

Dispatch constraints for simulations (Figure S46 only) that include 5% constrained natural gas dispatch.

Natural gas capacity was not constrained:

$$\sum_{t} D_t^{g,natural\ gas} \leq 5\% \cdot \sum_{g,t} D_t^g$$

Storage energy balance:

$$S_1 = (1-\delta^s)S_t\Delta t + \eta^s D_T^{to\ s}\Delta t - D_T^{from\ s}\Delta t \ \ \forall s$$

$$S_{t+1} = (1-\delta^s)S_t\Delta t + \eta^s D_t^{to\ s}\Delta t - D_t^{from\ s}\Delta t \ \ \forall s, t \in 1, \ldots, (T-1)$$

System energy balance:

$$\sum_{g} D_t^g \Delta t + D_t^{from\ s}\Delta t = M_t + D_t^{to\ so}\Delta t \ \ \forall g, t$$

Table S11. Model nomenclature.

Symbol	Unit	Description
g	kW	Generation technology (wind, solar, natural gas)
v	kW	Energy conversion (electrolyzer, fuel cell)
s	kWh	Energy storage (hydrogen storage, battery storage)
$from\ s$	kW	Discharge from energy storage
$to\ s$	kW	Charge to energy storage
t	H	Time step, starting from 1 and ending at T
$c_{capital}$	\$/kW for generation or conversion<br>\$/kWh for storage	(Overnight) capital cost
c_{fixed}	\$/kW/h for generation or conversion<br>\$/kWh/h for storage	Fixed cost
$c_{fixed\ O\&M}$	\$/kW/yr	Fixed operating and maintenance (O&M) cost
c_{var}	\$/kWh	Variable cost
f	-	Capacity factor (generation technology)
h	h/year	Average number of hours per year
i	-	Discount rate
n	yrs	Project life
Δt	h	Time step size, i.e., 1 hour in the model
C	kW for generation or conversion<br>kWh for storage	Capacity
D_t	kW	Dispatch at time step t
M_t	kWh	Demand at time step t
S_t	kWh	Energy remaining in storage at time step t

Symbol	Unit	Description
γ	1/yr	Capital recovery factor
δ	1/h	Storage decay rate
η	-	Storage charging efficiency
τ	h	Storage charging duration
